# Genome-wide transcriptome profile of rice hybrids with and without *Oryza rufipogon* introgression reveals candidate genes for yield

**DOI:** 10.1038/s41598-020-60922-6

**Published:** 2020-03-17

**Authors:** Haritha Guttikonda, Shashi Rekha Thummala, Surekha Agarwal, Satendra K Mangrauthia, Rajeshwari Ramanan, Sarla Neelamraju

**Affiliations:** 1 ICAR-Indian Institute of Rice Research, Hyderabad, 500 030 India; 2 CSIR-Centre for Cellular and Molecular Biology, Hyderabad, 500 007 India

**Keywords:** Gene expression profiling, Plant breeding

## Abstract

In this study, we compared genome-wide transcriptome profile of two rice hybrids, one with (test hybrid IR79156A/IL50-13) and the other without (control hybrid IR79156A/KMR3) *O. rufipogon* introgressions to identify candidate genes related to grain yield in the test hybrid. IL50-13 (Chinsurah Nona2 IET21943) the male parent (restorer) used in the test hybrid, is an elite BC_4_F_8_ introgression line of KMR3 with *O. rufipogon* introgressions. We identified 2798 differentially expressed genes (DEGs) in flag leaf and 3706 DEGs in panicle. Overall, 78 DEGs were within the major yield QTL *qyld2.1* and 25 within minor QTL *qyld8.2*. The DEGs were significantly (*p* < 0.05) enriched in starch synthesis, phenyl propanoid pathway, ubiquitin degradation and phytohormone related pathways in test hybrid compared to control hybrid. Sequence analysis of 136 DEGs from KMR3 and IL50-13 revealed 19 DEGs with SNP/InDel variations. Of the 19 DEGs only 6 showed both SNP and InDel variations in exon regions. Of these, two DEGs within *qyld2.1*, *Phenylalanine ammonia- lyase* (PAL) (Os02t0626400-01, *OsPAL2*) showed 184 SNPs and 11 InDel variations and *Similar to phenylalanine ammonia- lyase* (Os02t0627100-01, *OsPAL4*) showed 205 SNPs and 13 InDel variations. Both PAL genes within *qyld2.1* and derived from *O. rufipogon* are high priority candidate genes for increasing grain yield in rice.

## Introduction

Rice (*Oryza sativa*) is an important food crop providing 20% of daily calories to more than 50 percent of global population. Nearly 90% of rice is produced and consumed in Asia. The continuous increase in human population, especially in Asia, poses a major challenge to food security. Therefore, enhancing grain yield is the primary thrust area of plant breeders. Hybrid rice can help increase productivity by 10–20% more than conventional varieties^1^. Currently, the highest-yielding rice hybrids are developed from inter-subspecific crosses between *indica* and *japonica*^2–5^. Wild species have been used to breed parental lines for yield improvement in derived rice hybrids^6–10^.

Flag leaf is the most essential functional organ to produce a large proportion of photo-assimilates that are stored in grains^11,12^. It is estimated that it contributes around 32.3% of total carbohydrates during grain filling^13^. The panicle morphology also directly affects the number and size of seeds and also determines grain yield in rice^14–16^. Flag leaf and panicles have been used previously at different developmental stages to unravel gene expression in pollen development and genetic networks that control panicle branching and architecture^17,18^. The spatial and temporal expression profiles of genes during 19 vegetative and reproductive stages of organ development were analysed to identify stage-preferential/stage-specific genes in IR64 variety and anther-specific genes in Pusa Basmati1 variety^19,20^.

A whole-genome oligonucleotide microarray of super hybrid LYP9 (Liangyoupeijiu) and its parents 9311 and PA64s in 7 different tissues showed that differentially expressed genes for energy metabolism in first 3 stages of flag leaf and genes for transportation in next 3 stages of flag leaf were enriched in between the hybrid and parents rather than in between the parents^21^. Likewise, serial analysis of gene expression (SAGE) in roots, leaves and panicles of LYP9 showed that genes related to enhancing carbon-nitrogen assimilation pathways in leaves, nitrogen uptake in roots, protein biosynthesis and peptide transport were up-regulated in panicle of hybrids compared to respective tissues in parents^22^. SAGE analysis in leaves at grain filling stage of another super hybrid rice Liangyou-2186 and its parental lines SE21s and MH86 (Minghui86) showed differentially up-regulated genes related to photosynthesis and carbon fixation pathways in hybrid^23^. The whole-genome oligonucleotide microarray of flag leaves in three super-hybrid rice combinations LY2163 (SE21s x MH63), LY2186 (SE21s x MH86) and LYP9 (PA64s x 93-11) and their respective parental lines at flowering and grain filling stages showed that DEGs in all three super-hybrid combinations were significantly enriched in carbon fixation pathway, starch and sucrose metabolic pathway and flavonoid biosynthesis pathway^24^. The analysis showed heterotic gene RH8/DTH8/Ghd8/LHD1 is one of the loci contributing to yield heterosis in hybrid rice LYP9^25^. Thus, gene expression in hybrids points to some relationship between DEGs in carbon fixation pathway and heterosis. However, these studies were carried out only in inter-subspecific hybrids and comparison was made between parents and hybrids. The higher yielding hybrids derived from improved restorer lines having introgressions from wild species have not been used for whole-genome expression profiling previously. In this study we compare transcriptome of two hybrids one with *O. rufipogon* introgressions in its restorer and the other without *O. rufipogon* introgressions in the restorer to know what effect the *O. rufipogon* introgressions in the restorer have on the derived hybrid.

In our previous work, we mapped two yield-enhancing QTLs (quantitative trait loci) *qyld2.1* and *qyld8.2* from BC2 testcross progeny from IR58025A/*O. rufipogon*//IR580325B///IR58025B////KMR3^26^. IR58025A is a popular CMS line and KMR3 is restorer for the well adapted popular hybrid KRH2. The major yield enhancing QTL *qyld2.1* from *O. rufipogon* was dissected into 8 subQTL regions^27,28^. A total of 67 hybrids were developed using 27 of KMR3/*O. rufipogon* elite introgression lines (ILs) as restorers and 6 CMS lines as recipients. The highest yielding top 11 of 67 hybrids which had introgressions from *qyld2.1* showed 40.0–48.06 g yield per plant compared with respective control hybrids with 30.2–35.5.9 g^9^. An elite hybrid IR79156A/IL50-13 showed significantly high combining ability and standard heterosis for yield over popular hybrid DRRH2^9,10^. The test hybrid IR79156A/IL50-13 (with *O. rufipogon* introgression) showed yield advantage of 32% over the control hybrid IR79156A/KMR3 (without *O. rufipogon* introgression) during kharif season 2012 at IIRR (Indian Institute of Rice Research) farm, Rajendranagar, Hyderabad.

In addition the test hybrid IR79156A × IL50-13 [IET 24441 (DRRH 102)] was tested in multilocation trials of All India Coordinated Rice Improvement Program (AICRIP) in 2014. It gave mean grain yield of 2.2 t/ha and showed 14% yield advantage over the best salinity check variety CSR10 and 24% over the yield check variety Jaya and was therefore promoted to advanced variety trial 1 - Coastal Saline Tolerant Variety Trial (AVT 1 -CSTVT)^29^. In moderate salinity, the hybrid ranked first (5796 kg/ha) in overall mean yield and showed 57.9% yield advantage over CSR10. However, in severe coastal saline stress it gave mean grain yield of only 2.1t/ha but still showed 59.4% yield advantage over CSR10^30^. So the test hybrid is demonstrated to be high yielding and moderately tolerant to coastal salinity stress in field conditions. The control hybrid was not tested in AICRIP trials.

We selected this elite hybrid as test hybrid for further functional validation of *qyld2.1* and other heterotic loci derived from *O. rufipogon* associated with high yield and compared it with the respective control hybrid IR79156A/KMR3. This study was aimed to uncover the genes, molecular mechanisms and metabolic pathways which are involved in higher yield in test hybrid IR79156A/IL50-13 (with *O. rufipogon* introgressions) compared to control hybrid IR79156A/KMR3 (without *O. rufipogon* introgressions). Thus the difference is assumed to be due to heterotic loci from *O. rufipogon* introgressions.

## Results

### Transcriptome analysis

**Figure 1:**
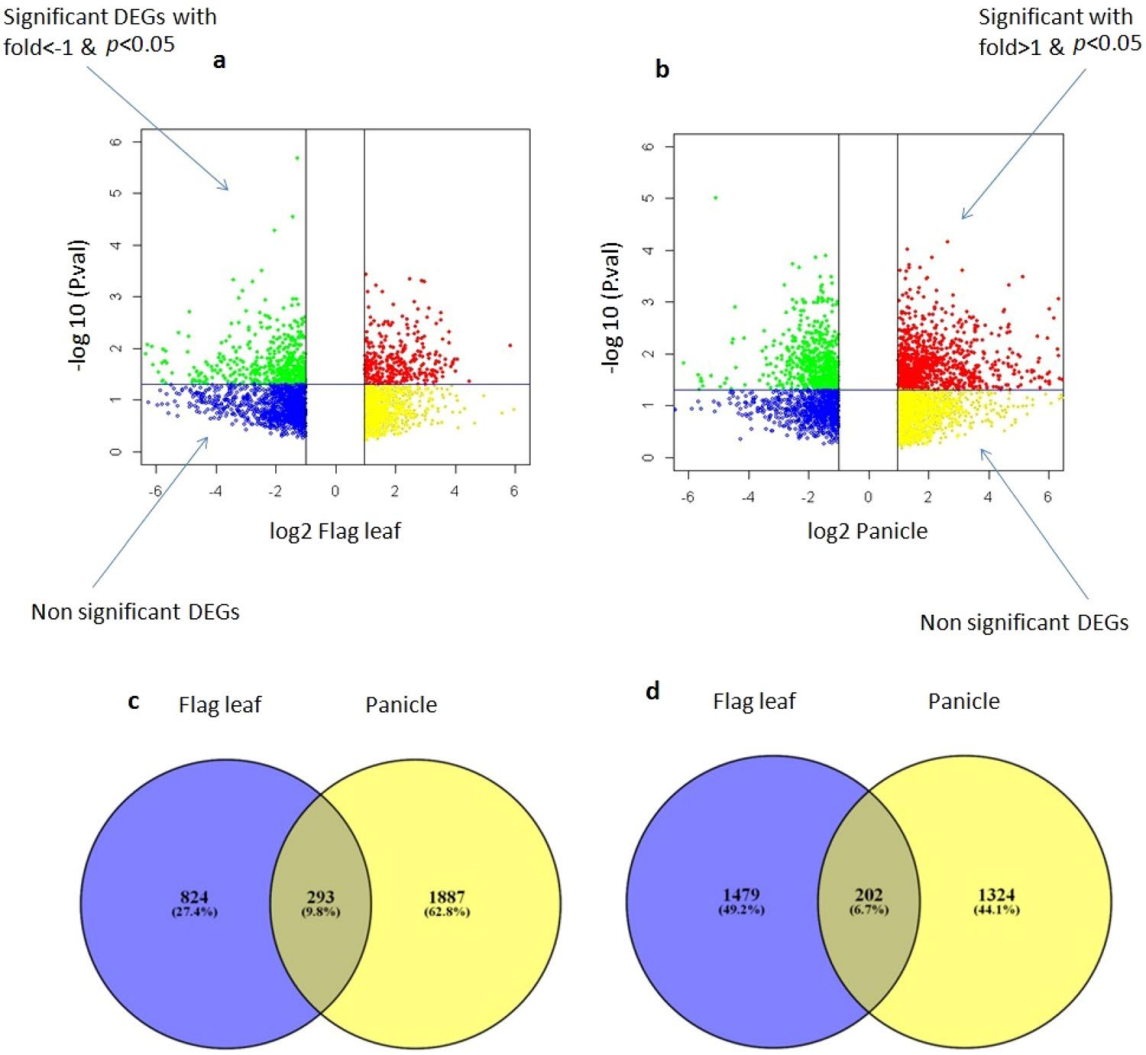
Volcano plot showing gene expression differences in (**a**) flag leaf and (**b**) panicles of test hybrid IR79156A/IL50-13 with *P*-values and intensity ratios as log-scaled axes. Significant differences at *p* < 0.05 with >1-fold and <1 fold intensity ratios are shown. Blue: Fold <= −1 & *p*-value >0.05, Yellow: Fold >= 1 & *p*-value > 0.05, Green: Fold <= −1 & *p*-value <0.05, Red: Fold >= 1 & *p*-value <0.05. (**c**) Venn diagrams of up-regulated genes (**d**) down-regulated genes in flag leaf and panicle of test hybrid in comparison with control hybrid.

The transcriptome of flag leaf of test hybrid was compared with that of control hybrid to know the effect of *O. rufipogon* introgressions in the hybrid. Likewise the panicle of test hybrid was compared with that of control hybrid. In all, 1117 up-regulated and 1681 down-regulated differentially expressed genes (DEGs) were identified in flag leaf of test hybrid (IR79156A/IL50-13) compared to control hybrid (IR79156A/KMR3). Of these, 363 genes were significantly up-regulated and 458 were significantly down-regulated (*p* < 0.05). Similarly, 2180 up-regulated and 1526 down-regulated DEGs were identified in panicle of test hybrid compared to control hybrid. In panicle, 931 genes were significantly up-regulated and 647 were significantly down-regulated (Supplementary Table S1). The number of DEGs showing an expression change of >2 fold with a significance of *p* < 0.05 were lower in flag leaf compared to panicle (Fig. 1a). However, more genes showed up-regulation in panicle than in flag leaf of test hybrid (Fig. 1b). The maximum up-regulation was 5.96 fold (Os02t0616100 - *similar to protein binding protein*) in flag leaf and 8.21 fold (Os08t0474000 - *similar to AP2 domain containing protein* RAP2.6) in panicle. The maximum down- regulation was −8.7 fold (Os01t0538000 - *conserved hypothetical protein*) in flag leaf and −10.02 fold (Os01t0579000 - *conserved hypothetical protein*) in panicle of test hybrid. Since our focus was on the yield enhancing QTLs mapped previously, we first analyzed DEGs within *qyld2.1* on chromosome 2 and *qyld8.2* on chromosome 8 in more detail.

A Venn diagram was constructed to demonstrate common and exclusively expressed DEGs in flag leaf and panicle using total number of DEGs. Panicle had highest number (1887 = 86.5%) of exclusively up-regulated DEGs (Fig. 1c). On the other hand, flag leaf had highest number (1479 = 87.9%) of exclusively down-regulated DEGs (Fig. 1d). There were 293 up-regulated and 202 down-regulated DEGs common in flag leaf and panicle of test hybrid when compared to control hybrid. The details of DEGs with fold change are given in Supplementary Table S2.

### DEGs within yield QTLs *qyld2.1* and *qyld8.2*

Comparing the test hybrid with control hybrid, we found 78 DEGs within the major yield QTL *qyld2.1* (5 Mb) and 25 DEGs within the minor yield QTL *qyld8.2* (2 Mb). Of the 78 DEGs within *qyld2.1*, 12 DEGs were up-regulated and 21 were down-regulated in flag leaf while 29 DEGs were up-regulated and 16 were down-regulated in panicle (Supplementary Table S3). It is important to note that 3 DEGs within *qyld2.1 -* Os02t0596300-01 (*Cytochrome P450 family protein*), Os02t0618700 (*Transmembrane receptor, eukaryota domain containing protein*) and Os02t0571800-00 (*Terpene synthase-like domain containing protein*) were down-regulated in both flag leaf and panicle of test hybrid, whereas four DEGs Os02t0559800-01 (*E3 ubiquitin ligase EL5*), Os02t0624300-01 (*Similar to Y19 protein*), Os02t0629000-01 (*Protein of unknown function DUF584 family protein*) and Os02t0627100-01 (*Similar to phenylalanine ammonia- lyase*) were down-regulated in flag leaf but up-regulated in panicle. Only two DEGs Os02t0552700-01 (*Zinc finger, CCHC-type domain containing protein*) and Os02t0596200-01 (*Glycoside hydrolase family 5 protein*) showed up- regulation in both flag leaf and panicle of test hybrid. Similarly, analysis of 25 DEGs within *qyld8.2* showed 5 DEGs were up-regulated and 4 DEGs were down-regulated in leaf, whereas 9 DEGs were up-regulated and 7 DEGs were down-regulated in panicle of test hybrid compared to control hybrid respectively. It is significant that 3 DEGs Os08t0468100-01, Os08t0468100-02 and Os08t0468100-03, all ‘*Similar to nitrate reductase*’ were up-regulated in both flag leaf and panicle of test hybrid.

### Expression of DEGs associated with grain yield and abiotic stress

There were 52 transcription factors in flag leaf and 59 in panicle which showed more than 2- fold differential expression in test hybrid (Supplementary Table S4). The maximum up-regulation in flag leaf (3.8 fold) was shown by *squamosa promoter binding protein-like transcription factor* (Os08t0509600-01) and maximum up-regulation in panicle (8.2 fold) was shown by *similar to AP2 domain containing protein* RAP2.6 (Os08t0474000-01) within the minor yield QTL *qyld8.2*. It is interesting to note that genes known to be associated with abiotic stress tolerance eg. encoding *salt-induced protein* (Os01g0348900), *similar to OsNAC6 protein* (Os03g0815100), *WRKY transcription factor 74* (Os09g0334500), *late embryogenesis abundant protein 3 family protein* (Os01g0314800) and *similar to KUP related potassium transporter* (Os08g0466200) were significantly up-regulated in panicle of test hybrid even though the plants were not exposed to any abiotic stress (Supplementary Table S5). It is likely that the higher expression of these genes in panicle contributes to its salinity tolerance and high yield in multilocation trials in coastal saline areas.

In addition, two DEGs Os06t0248300-03 (LOC_Os06g13850.1, Conserved hypothetical protein, F-box protein), Os01t0842500-01 (LOC_Os01g62490.1, Similar to laccase) were down-regulated in panicle and Os03t0150800-01 (LOC_Os03g05640.1, inorganic phosphate (Pi) transporter, Pi homeostasis, Selenite uptake) was up-regulated in panicle and down-regulated in flag leaf. These 3 genes known to be associated with plant architecture and grain yield were identified as microRNA target genes in test hybrid.

### Functional annotation and classification of DEGs based on gene ontology (GO)

Gene ontology analysis was performed to classify the functional categories of DEGs in both tissues of test hybrid. There were 109 terms for up-regulated DEGs and 91 terms for down-regulated DEGs enriched in flag leaf (Supplementary Table S6). Similarly 143 terms for up-regulated DEGs and 111 for down-regulated DEGs were enriched in panicle of test hybrid (Supplementary Table S7). Out of these, 53 terms were expressed only in flag leaf and 93 terms were expressed only in panicle. There were 104 terms that were common to both flag leaf and panicle of test hybrid (Supplementary Table S8).

**Figure 2:**
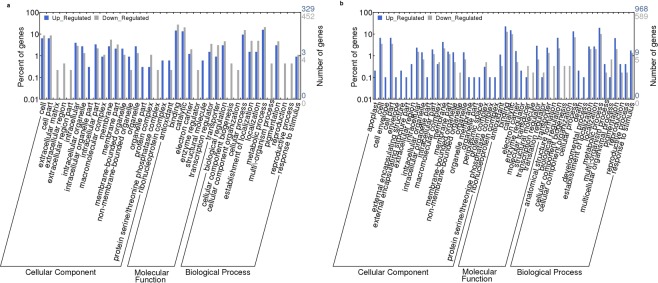
Gene Ontology enrichment analysis of differentially expressed genes by WEGO (Web Gene Ontology Annotation Plot -http://wego.genomics.org.cn/cgi-bin/wego/index.pl). The *P*-value is below the significance level of *p* < 0.05. Percentage of enrichment is also shown (**a**) flag leaf (**b**) panicle.

Further, the top GO terms that were significantly (*p* < 0.05) down-regulated in flag leaf were membrane-bounded vesicle (GO: 0031988, P = 0.0486) and cytoplasmic membrane-bounded vesicle (GO: 0016023, P = 0.0486) in cellular component category, while for molecular function category, catalytic activity (GO: 0003824, P = 0.0221) was most predominant term (Fig. 2a). Likewise, in panicle hemi cellulose metabolic process (GO: 0010410, P = 0.0202), xylan catabolic process (GO: 0045493, P = 0.0202), cell wall polysaccharide metabolic process (GO: 0010383, P = 0.0202), xylan metabolic process (GO: 0045491, P = 0.0202) and response to stress (GO: 0006950, P = 0.0584) were most significantly up-regulated terms in biological process category (Fig. 2b). This indicates that significantly over represented genes in this category play an extensive role in xylan and cell wall metabolic processes that may be involved in grain yield in test hybrid.

### KEGG (kyoto encyclopedia of genes and genomes) enrichment analysis

Pathway analysis was performed using KEGG database to understand the biological functions and enriched metabolic pathways of DEGs in test hybrid. The annotations revealed that 100 metabolic pathways (182 DEGs) were up-regulated and 196 pathways (473 DEGs) down-regulated in flag leaf (Supplementary Table S9). Likewise, 166 pathways (342 DEGs) were up-regulated and 121 pathways (229 DEGs) were down-regulated in panicle of test hybrid (Supplementary Table S10). There were 232 pathways common between leaf and panicle. Four major pathways - starch synthesis, ionic stress related calmodulin pathway, transcription factor related and proteasome related (*E3 Ubiquitin ligase*) showed high fold up-regulation in panicle but down-regulation in flag leaf of test hybrid compared to control hybrid.

Mapman analysis allowed the exploration of metabolic pathways which were activated specifically in test hybrid. The analysis revealed that the DEGs involved in major metabolic pathways such as carbohydrate metabolism, phenyl propanoid pathway, ubiquitin - dependent degradation and jasmonic acid pathways play a key role in contributing to grain yield in test hybrid. Since our interest was on grain yield in test hybrid as compared to control hybrid, we performed the Mapman analysis for DEGs within *qyld2.1* (Supplementary Fig. S1a,b) and *qyld8.2* (Supplementary Fig. S2a,b).

**Figure 3:**
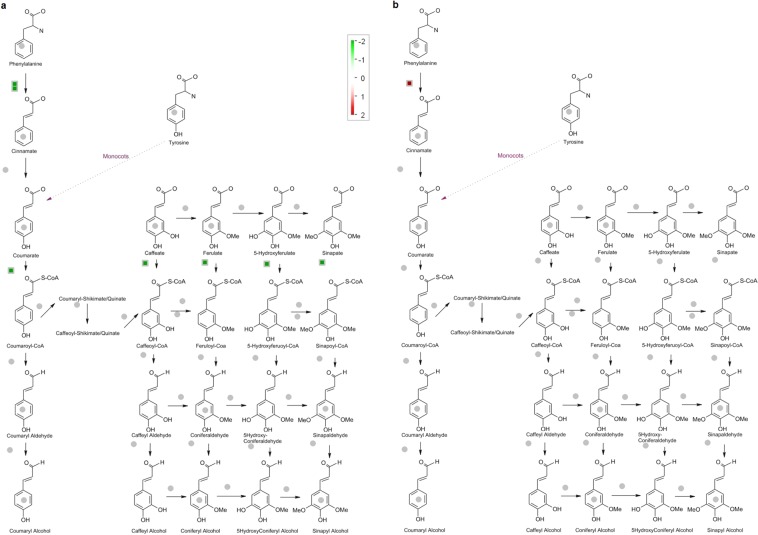
Phenyl propanoid biosynthesis pathway in rice. The green squares represent down-regulation and red squares represent up-regulation in test hybrid (**a**) flag leaf (**b**) panicle.

**Figure 4:**
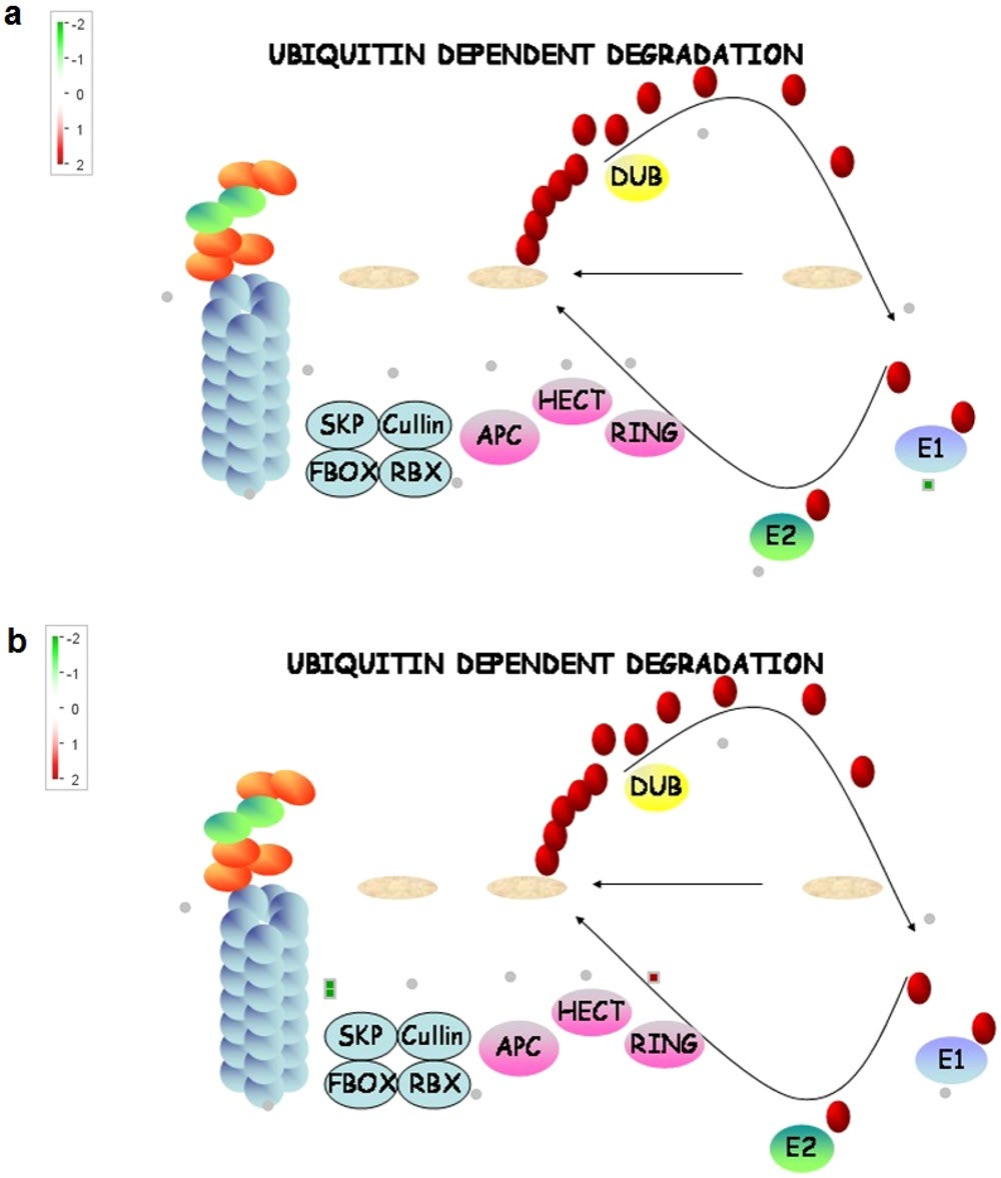
Schematic representation of the ubiquitination reaction involving a HECT-type E3. The green squares represent down-regulation and red squares represent up-regulation in test hybrid (**a**) flag leaf (**b**) panicle.

Carbohydrate metabolism overview revealed involvement of the down-regulated gene Os03t0401300-01 (*Sucrose synthase 2*) in sucrose-starch metabolism (Supplementary Fig. 3a), plant glycolysis, cell wall precursors and raffinose metabolism in flag leaf while in panicle, the up-regulated gene Os10t0465700-01 (*Similar to beta amylase PCT-BMYI*) which acts on α-1, 4 glycosidic bonds showed involvement in plant glycolysis and sucrose-starch metabolism (Supplementary Fig. 3b). Phenyl propanoid metabolism overview revealed that down-regulated DEGs Os02t0626400-01 (*Phenyl alanine ammonia-lyase*), Os02t0626100-01 (*Similar to phenyl alanine ammonia-lyase*), Os08t0498100-01 (*Similar to caffeoyl coA methyl transferase 2*) in flag leaf and up-regulated DEG Os08t0448000-01 (*Similar to coumarate coA ligase 1*) in panicle were involved in secondary metabolism such as biosynthesis of lignin, suberin, salicylate, piperidine and pyridine alkaloid, ubiquinone and other terpenoid-quinone and threonine (Fig. 3a,b). The ubiquitin metabolism overview clearly revealed the involvement of Os02t0559800-01 (*E3 ubiquitin ligase*) in protein ubiquitination. This is one of the important pathways for improving grain yield in rice and also regulates cell cycle. Notably this gene was down-regulated in flag leaf (Fig. 4a) and up-regulated in panicle (Fig. 4b). Jasmonic acid pathway analysis showed that Os12t0559200-01 (*lipoxygenase-LOX*) is down-regulated in flag leaf. It is involved in the conversion of linolenic acid to 13(S) hydroperoxylinolenic acid a precursor for jasmonic acid biosynthesis. The expression of LOX gene also depends on the functional activity of ubiquitin ligase which was down-regulated in flag leaf of test hybrid (Supplementary Fig. S4).

### qRT-PCR validation of differentially expressed genes

A set of 22 DEGs were selected to validate the microarray results with quantitative reverse transcription PCR (qRT-PCR) including 5 genes within *qyld2.1*, five genes within *qyld8.2*, and 12 DEGs associated with yield but located on other chromosomes (Table 1). All 22 DEGs showed reproducibility and were significantly correlated with the microarray data. In these 22 genes, 8 were differentially down-regulated and one gene was up-regulated only in flag leaf (Supplementary Fig. S5a), 9 genes were differentially up-regulated in panicle (Supplementary Fig. 5b) and 4 genes showed differential expression in both flag leaf and panicle (Supplementary Fig. 5c). The degree of expression was higher in panicle than in flag leaf of test hybrid. The expression patterns of these 22 yield related genes were in good agreement with the quantification of transcripts done through microarray, though the degree of expression varied.

**Table 1:** Primer sequences of 22 selected genes used for qRT-PCR.

S.No	Locus Id	Gene Name	Primer sequence (5′-3′)
1	**Os02t0559800-01** chr02:21250083..21251343	**E3 ubiquitin ligase EL5**	F: GTGATCGAGATCCCCGAAT R: CCAGAGCCTTCTCAGTGACC
2	**Os02t0624300-01** chr02:24878777..24879932	**Similar to Y19 protein**	F: TCCGGTGGATCAACTACCTC R: GTTGCCGAGAAGGTCGTG
3	**Os08t0482600-01** chr08:23853339..23854314	**Cupredoxin domain containing protein**.	F: TCTGATCGCAATGCTCCTC R: CGTTAGCCTCGAAGGTTTTG
4	**Os08t0473900-03** chr08:23341357..23343240	**Similar to Alpha-amylase isozyme 3D**	F: AGCTTGTGTTTGCTGCTCCT R: CTGGAAGAGGACCTGTGCTT
5	**Os08t0473900-01** chr08:23341289..23343273	**Alpha-amylase isozyme 3D precursor (EC 3.2.1.1) (1,4-alpha-D-glucan glucanohydrolase)**	F: GTGAAGATCGGGACGAGGTA R: CCTTCTCCCAGACGCTGTAG
6	**Os08t0466200-01** chr08:22904756..22908287	**Similar to KUP-related potassium transporter**	F: CTTCAGCAAGAGGTCCATCC R: GTTGATCAGGTACGCCGTCT
7	**Os02t0569900-01** chr02:21740304..21741960	**Cytochrome P450 family protein-1500bp**	F: ACACGTTCGAGTGGAAGCTC R: CAAGCACATTGGCAGACTTG
8	**Os02t0626400-01** chr02:24985294..24989388	**Phenylalanine ammonia-lyase (EC 4.3.1.5)**	F: ACCACCTGACACACAAGCTG R: TACGAGCTGCCTTCCAAGAT
9	Os03t0753100-01 chr03:31048351..31055017	MADS-box transcription factor, Inflorescence and spikelet development	F: GATCGACGTAGAGGCAGCTC R: GGAGGCTCACTGGAAAACAC
10	Os10t0531400-01 chr10:20645931..20646880	Glutathione S-transferase GST 30 (EC 2.5.1.18)	F: GAAGCTACTGGGGATGTGGA R: CTCGTACGGCAGTGACTTGA
11	Os03t0401300-01 chr03:16301279..16306089	Sucrose synthase 2 (EC 2.4.1.13) (Sucrose-UDP glucosyltransferase 2)	F: GCTGAAGGACAGGAACAAGC R: CAGCTCAACCAGACCAGTCA
12	Os01t0348900-01 chr01:13903285..13904626	SalT gene product (Salt-induced protein)	F: CTGGAGTCCCAAATGGAAAG R: CGTTCCAGACCTTCCAAAGA
13	Os01t0314800-01 chr01:11863682..11864412	Late embryogenesis abundant protein 3 family protein.	F: GGCGGACGAGAAGAAGGT R: GCCGGTAGTACCCGGTCA
14	Os03t0815100-01 chr03:34166100..34167521	Similar to OsNAC6 protein	F: GATGATGGTGCCCAAGAAAG R: AACAGGCTGCTGTTGTTCCT
15	Os04t0180400-01 chr04:5484865..5486703	Similar to Cytochrome P450 99A2	F: GCTCCTACCCAAAGCTGATG R: CATTATCCGGGGACAAACAT
16	Os09t0334500-01 chr09:10128837..10131086	WRKY transcription factor 74	F: ACGGTGTTCGACGTGATCTA R: CGTGTCCGTCTCCGTCTC
17	Os12t0559200-01 chr12:22854749..22860198	Lipoxygenase (EC 1.13.11.12)	F: CTCCATCAAGGAGTGGGTGT R: CTGGAGCTCCTTGTCCATGT
18	Os08t0498100-01 chr08:24586613..24587908	Similar to Caffeoyl-CoA O-methyltransferase 2 (EC 2.1.1.104)	F: ATCGAGGTGGGTGTCTTCAC R: TCTCGTAGCTCTCCCTGTCC
19	Os02t0626100-01 chr02:24973450..24977287	Similar to Phenylalanine ammonia-lyase	F: AGCGAGTGGATCCTCAACTG R: CTGAGGAGCTCGACTTGGA
20	Os10t0465700-01 chr10:17180759..17183189	Similar to Beta-amylase PCT-BMYI (EC 3.2.1.2)	F: GTGGTGGAGGAGATGGAGAA R: CCGAGGGAGATGTACTCGAA
21	Os08t0448000-01 chr08:21873076..21875497	Similar to 4 coumarate coA ligase1	F: TCCACGTCTACTCCCTCCAC R: GCAGCATCTTGACGGTGTC
22	Os02t0738200-01 chr02:30805988..30807610	Zinc finger, RING/FYVE/PHD-type domain containing protein	F: AGCTGCTCAGGCTTCTCAAC R: GTCTCTTCAGCCCCTTGAAA
23	OsACT1 Lee *et al*. (2011)	actin	F: TGGAAGCTGCGGGTATCCAT R: TACTCAGCCTTGGCAATCsCACA

Genes underlying yield QTLs *qyld2.1* and *qyld8.2* are shown in bold.

### Annotation of 22 validated DEGs

The KEGG annotation of 22 DEGs used for qRT-PCR validation showed 11 DEGs were involved in various metabolic pathways in test hybrid (Table 2). These included, 4 DEGs (Os08t0473900-03, Os08t0473900-01, Os03t0401300-01, Os10t0465700-01) related to sucrose and starch metabolism, 4 DEGs (Os02t0626400-01, Os02t0626100-01, Os08t0498100-01, Os08t0498100-01) related to phenyl propanoid biosynthesis, JA biosynthesis pathway gene (Os12t0559200-01), momilactone biosynthesis gene (Os04t0180400-01), and glutathione mediated detoxification pathway gene (Os10t0531400-01). In these, Os02t0626400-01 (*Phenyl alanine ammonia- lyase*) within *qyld2.1* is involved in phenyl propanoid biosynthesis, suberin synthesis and salicylate biosynthesis pathways. Two DEGs Os08t0473900-03 and Os08t0473900-01 (*Similar to alpha-amylase isozyme 3D* and *alpha-amylase isozyme 3D precursor* (*1,4-alpha-D-glucan glucanohydrolase*) within *qyld8.2* are involved in starch degradation.

**Table 2:** The expression pattern of 22 DEGs validated through qRT-PCR and KEGG pathway analysis results in test hybrid, ↓ denotes down-regulation and ↑ denotes up-regulation.

S. No	Gene name	Description	Microarray		qRT-PCR		KEGG pathway analysis
			Leaf	Panicle	Leaf	Panicle	
1	Os02t0559800-01	E3 ubiquitin ligase EL5	↓	↑	↓	↑	—
2	Os02t0624300-01	Similar to Y19 protein	↓	↑	↓	↑	—
3	Os08t0482600-01	Cupredoxin domain containing protein.		↑		↑	
4	Os08t0473900-03	Similar to Alpha-amylase isozyme 3D		↑		↑	Starch degradation
5	Os08t0473900-01	Alpha-amylase isozyme 3D precursor		↑		↑	Starch degradation
6	Os08t0466200-01	Similar to KUP-related potassium transporter.		↑		↑	
7	Os02t0569900-01	Cytochrome P450 family protein-1500bp	↓		↓		
8	Os02t0626400-01	Phenylalanine ammonia-lyase	↓		↓		Suberin biosynthesis, phenyl propanoid bio synthesis and initial reactions, salycilate biosynthesis
9	Os03t0753100-01	MADS-box transcription factor, Inflorescence and spikelet development	↑		↑		
10	Os10t0531400-01	Glutathione S-transferase GST 30	↓		↓		Glutathion mediated detoxification
11	Os03t0401300-01	Sucrose synthase 2	↓		↓		Sucrose degradation to ethanol and lactate (anaerobic), galactose degradation II, starch and sucrose metabolism, sucrose degradation III, sucrose biosynthesis
12	Os01t0348900-01	SalT gene product (Salt-induced protein).		↑		↑	
13	Os01t0314800-01	Late embryogenesis abundant protein 3 family protein		↑		↑	
14	Os03t0815100-01	Similar to OsNAC6 protein		↑		↑	
15	Os04t0180400-01	Similar to Cytochrome P450 99A2	↓	↑	↓	↑	Momilactone biosynthesis, nicotine degradation II, nicotine degradation III, bupropion degradation, acetone degradation to methyl glyoxal
16	Os09t0334500-01	WRKY transcription factor 74.		↑		↑	
17	Os12t0559200-01	Lipoxygenase	↓		↓		Biosynthesis of plant harmones, JA biosynthesis, alpha-lenolenic acid metabolism, 13-LOX and 13 HPL pathway, divinyl ether biosynthesis II, lenoleic acid metabolism
18	Os08t0498100-01	Similar to Caffeoyl-CoA O-methyltransferase 2	↓		↓		Phenyl propanoid biosynthesis, suberin biosynthesis
19	Os02t0626100-01	Similar to Phenylalanine ammonia-lyase.	↓		↓		Phenyl propanoid biosynthesis, suberin biosynthesis, phenyl alanine metabolism, phenyl propanoid biosynthesis initial reactions, salicylate biosynthesis, Tropane, piperidine and pyridine alkaloid biosynthesis, nitrogen metabolism
20	Os08t0448000-01	Similar to 4-coumarate-CoA ligase 1		↑		↑	Biosynthesis of phenyl propanoids, Biosynthesis of alkaloids derived from shikimate pathway, phenyl propanoid biosynthesis, metheonine biosynthesis II, Ubiquinone and other terpenoid-quinone biosynthesis, threonine biosynthesis from homoserine
21	Os10t0465700-01	Similar to Beta-amylase PCT-BMYI	↓	↑	↓	↑	starch degradation
22	Os02t0738200-01	Zinc finger, RING/FYVE/PHD-type domain containing protein.	↓		↓		

In panicle, two DEGs within *qyld8.2* were up-regulated. Os08t0473900-03 (*Similar to alpha-amylase isozyme 3D*) was classified in metabolic process, carbohydrate metabolic process and primary metabolic process in biological process terms, and hydrolase activity, hydrolyizing O-glycosyl compounds, acting on glycosyl bonds, binding, ion binding, metal ion binding, cation binding, calcium ion binding, catalytic activity, amylase activity, alpha-amylase activity were enriched terms in molecular function category. Another DEG Os08t0482600-01 (*Cupredoxin domain containing protein*) was classified in cation binding activity, transition metal ion binding, electron carrier activity and copper ion binding activity which were enriched terms in molecular function category.

### Sequence polymorphism of DEGs and other reported yield-related genes (not DEGs) between the restorer lines KMR3 and IL50-13 used for producing hybrids

Since the genome sequences of restorer lines KMR3 and IL50-13 were available (unpublished), they were compared only for DEGs within *qyld2.1* (78 DEGs), *qyld8.2* (25 DEGs) and 12 other DEGs based on significance (*p* < 0.05) and fold change value above 2. In addition, we also investigated the sequence differences of 21 reported yield-related genes between KMR3 and IL50-13 on different chromosomes. Out of 136 genes analysed, 117 sequences were identical between the two genomes (KMR3 and IL50-13) and only 19 genes (16 DEGs and 3 yield-related genes) showed differences between KMR3 and IL50-13 in terms of SNPs (single nucleotide polymorphism) and InDels (insertion and deletion polymorphism) (Supplementary Table 11). Of the 19 genes, one DEG Os08t0468100-03 (*Similar to nitrate reductase*) had unique InDels and 9 DEGs had unique SNPs and the other 9 genes showed both SNPs and InDel variations (Table 3). We obtained a total of 1073 variants in the 19 genes, of which 959 were SNPs and 114 were InDels (Table 4). The maximum number of variants were observed for the gene Os02t0592000-00, which is a *similar to OSIGBa0106G07.8 protein* and there were seven genes that had only one SNP/InDel variation. We compared the SNPs and InDels present in different regions (coding DNA sequence CDS (exons), untranslated regions UTRs (5′ and 3′UTRs) and introns) of these 19 genes. Of the 959 SNPs in 19 genes, CDS had the maximum number of SNPs (541) followed by introns (413) and the least number was found in UTRs (5). But considering 114 InDels found in these 19 genes, introns had the maximum number of SNPs (77) followed by CDS (36) and UTRs had the least number (1) of SNPs. Six DEGs Os02t0626400-03 (84), Os02t0569800-00 (100), Os02t0616600-01 (108), Os02t0592000-00 (195), Os02t0626400-01 (184) and Os02t0627100-01 (205) within *qyld2.1* showed high number of SNP variations in IL50-13 compared to KMR3 (Table 4). The study of SNPs in coding region and 2 kb upstream/downstream of 19 genes (16 DEGs and 3 yield reported genes) identified only one gene Os02t0831500-01 (*Similar to sucrose synthase*) which showed differences in terms of SNPs. We obtained three unique SNPs in the upstream region of Os02t0831500-01 in IL50-13 that showed a modifying effect on the gene (Supplementary Table S12).

**Table 3:** DEGs and reported yield related genes showing difference between the sequences of KMR3 and IL50-13.

S. No	Category	Gene Name	Description	Gene length	KMR3 scaffold	Start position of scaffold	End position of scaffold	50-13 scaffold	Start position of scaffold	End position of scaffold	Differences between KMR3 and 50-13 scaffolds
1	A28	Os02t0589000-01	Lecithin: cholesterol acyltransferase family protein	10391	scaffold9446_size10065	984	3580	scaffold14856_size6220	1	2790	SNPs + InDels
2	A61	Os02t0626400-03	Similar to Phenylalanine ammonia-lyase	1589	scaffold13385_size7025	3655	4920	scaffold12144_size7524	3315	2050	SNPs + InDels
3	A11	Os02t0569800-00	Hypothetical genes	4024	scaffold2303_size25555	19748	21711	scaffold17631_size5156	2282	3988	SNPs + InDels
4	A51	Os02t0616600-01	Conserved hypothetical protein	3728	scaffold15492_size5752	3500	5752	scaffold26405_size2842	474	2842	SNPs + InDels
5	A32	Os02t0592000-00	Similar to OSIGBa0106G07.8 protein	9817	scaffold8341_size11245	7965	10401	scaffold17585_size5169	1236	2636	SNPs + InDels
6	A16	Os02t0574800-01	Ethylene insensitive 3 domain containing protein	739	scaffold14052_size6604	4	276	scaffold21804_size3915	3915	3667	SNPs
7	A1	Os02t0552500-00		1185	scaffold23485_size2774	2774	1848	scaffold30709_size2103	415	1	SNPs
8	A12	Os02t0569900-01	Cytochrome P450 family protein	1657	scaffold7129_size12786	9197	10493	scaffold24827_size3169	3169	2828	SNPs
9	A62	Os02t0626400-01	Phenylalanine ammonia-lyase (EC 4.3.1.5)	2593	scaffold13385_size7025	2826	4920	scaffold12144_size7524	4141	2050	SNPs + InDels
10	A63	Os02t0627100-01	Similar to Phenylalanine ammonia-lyase (EC 4.3.1.5)	2405	scaffold7515_size12279	1251	3210	scaffold12144_size7524	4200	2051	SNPs + InDels
11	B8	Os08t0468100-03	Similar to Nitrate reductase.	2053	scaffold32698_size2633	1589	1	scaffold5531_size12871	3714	1775	InDels
12	B21	Os08t0487301-00	-	485	scaffold243_size48649	4120	3822	scaffold6702_size11543	11094	11491	SNPs + InDels
13	B23	Os08t0490100-01	Similar to PBF protein	1261	scaffold8993_size10535	8750	9323	scaffold3687_size21453	15734	15885	SNPs + InDels
14	C1	Os01t0314800-01	Late embryogenesis abundant protein 3 family protein.	731	scaffold2182_size25019	20065	19335	scaffold17822_size5094	3714	4149	SNPs
15	C4	Os02t0831500-01	Similar to Sucrose synthase	6271	scaffold8577_size10987	1	2889	scaffold5992_size12325	12325	9137	SNPs
16	C10	Os06t0229800-01	Similar to Starch synthase IIA.	4905	scaffold1456_size29059	29059	24569	scaffold8945_size9534	1	3623	SNPs
17	D6	Os03t0407400-00	-	945	scaffold14776_size6169	2241	1683	scaffold6119_size12184	6218	6724	SNPs
18	D8	Os03t0117900-01	-	955	scaffold9265_size10248	5473	6424	scaffold21042_size4123	1	446	SNPs
19	D19	Os08t0509600-01	Rice squamosa promoter binding protein-like 14.	4156	scaffold3452_size19961	487	4642	scaffold2693_size19548	108	3677	SNPs

Category A refers to genes within *qyld2.1*, B refers to genes within *qyld8.2*, C refers to genes showing high fold expression and D refers to reported yield related genes.

**Table 4:** Polymorphism observed in terms of SNPs and InDels between sequences of KMR3 and IL50-13 for the DEGs and reported yield related genes.

S. No.	Category	Gene Name	No. of variants	No. of SNPs	No. of SNPs (CDS)	No. of SNPs (UTRs)	No. of SNPs (Introns)	No.of InDels	No.of InDels (CDS)	No.of InDels (UTRs)	No.of InDels (Introns)
1	A28	Os02t0589000-01	13	10	0	0	10	3	0	0	3
2	A61	Os02t0626400-03	86	84	84	0	0	2	2	0	0
3	A11	Os02t0569800-00	118	100	4	0	96	18	0	0	18
4	A51	Os02t0616600-01	123	108	0	5	103	15	0	1	14
5	A32	Os02t0592000-00	230	195	0	0	195	35	0	0	35
6	A16	Os02t0574800-01	1	1	1	0	0	0	0	0	0
7	A1	Os02t0552500-00	3	3	3	0	0	0	0	0	0
8	A12	Os02t0569900-01	36	34	34	0	0	2	2	0	0
9	A62	**Os02t0626400-01**	195	184	184	0	0	11	11	0	0
10	A63	**Os02t0627100-01**	218	205	205	0	0	13	13	0	0
11	B8	Os08t0468100-03	7	1	0	0	1	6	0	0	6
12	B21	Os08t0487301-00	11	9	5	0	4	2	2	0	0
13	B23	Os08t0490100-01	23	17	17	0	0	6	6	0	0
14	C1	Os01t0314800-01	1	1	1	0	0	0	0	0	0
15	C4	Os02t0831500-01	1	0	0	0	0	1	0	0	1
16	C10	Os06t0229800-01	1	1	1	0	0	0	0	0	0
17	D6	Os03t0407400-00	1	1	1	0	0	0	0	0	0
18	D8	Os03t0117900-01	1	1	1	0	0	0	0	0	0
19	D19	Os08t0509600-01	4	4	0	0	4	0	0	0	0
		Total	1073	959	541	5	413	114	36	1	77

Category A refers to genes within *qyld2.1*, B refers to genes within *qyld8.2*, C refers to genes showing high fold expression and D refers to reported yield related genes. The candidate genes (PAL) showing maximum SNPs in CDS and within *qyld2.1* are shown in bold.

## Discussion

To explore the genes and mechanisms involved in heterosis for yield in test hybrid, we profiled the genome-wide transcriptomes of flag leaves and young panicles in test hybrid IR79156A/IL50-13 and control hybrid IR79156A/KMR3. Both hybrids were developed using same cytoplasmic male sterile (CMS) line IR79156A, but two cognate restorer lines one with *O. rufipogon* introgression (IL50-13) and other without *O. rufipogon* introgression (KMR3). Such a comparative analysis of hybrids has not been made before. Our previous work showed that the hybrid IR79156A/IL50-13 gave significantly high yield 43 g/plant and showed highest specific combining ability (SCA) for yield (6.04) among 36 hybrids and high standard heterosis over KRH2 (61.79%) and DRRH2 (50.23%) indicating predominance of non-additive gene action for grain yield heterosis in test hybrid^9,10^. Several studies showed that non-additive gene action is important for yield and yield related traits in rice hybrids^31–33^. Similar results were obtained for yield heterosis in maize hybrids^34–36^. Based on the above analysis, we hypothesised that this yield heterosis might be due to the new genetic variation introduced from *O. rufipogon* via the restorer line and which enhances/regulates the yield potential of test hybrid.

Since, the restorer line IL50-13 is derived from an interspecific cross of KMR3/*O. rufipogon*, we focussed primarily on the DEGs within major yield QTL *qyld2.1* and minor effect QTL *qyld8.2* reported previously^26^. We indeed found 78 DEGs within *qyld2.1* and 25 DEGs within *qyld8.2* (Supplementary Table S3). The prominent DEGs within *qyld2.1* were *E3 ubiquitin ligases*, *phenylalanine ammonia-lyases* and *cytochrome P450 family proteins* and DEGs within *qyld8.2* were *alpha-amylases*, *nitrate reductases* and *similar to KUP related potassium ion transporters*. Interestingly, the highest (5.96) fold up-regulated gene Os02t0616100-01 (*Similar to protein binding protein*) in flag leaf lies within *qyld2.1*, and the highest (8.21) fold up-regulated DEG in panicle Os08t0474000-01 (*Similar to AP2 domain containing protein RAP2.6*) lies within *qyld8.2*. These two DEGs are worth further study. However, the GO enrichment analysis revealed cytoplasmic membrane-bound vesicle and catalytic activity are most significant terms in flag leaf whereas, hemi cellulose metabolic process, cell wall polysaccharide metabolic process, xylan metabolic process, antioxidant and response to stress are most significant terms in panicle of test hybrid (Supplementary Tables S6 and S7). Thus these processes are associated with grain yield and tolerance to salt stress (since the hybrid gave high yield in coastal salinity). Starch synthesis, phenyl propanoid pathway, ubiquitin dependent degradation and phytohormone related pathways were highly enriched pathways in test hybrid according to KEGG analysis (Supplementary Tables S9 and S10). The transcriptomic analysis of super hybrid rice LYP9 and its parents 93-11 and PA64s showed that the genes in the categories of energy metabolism and transport are enriched in between the hybrid and its parents rather than in between the parents^21^. Similarly the comparative transcriptional profile of three super hybrids LY2163, LY2186, LYP9 showed the DEGs were significntly (*p* < 0.01) enriched in carbon fixation pathway in all 3 super-hybrid combinations compared to its parents^24^.

We found some genes related to salinity tolerance and other abiotic stresses were also differentially expressed in the test hybrid even though the plants were not exposed to any abiotic stress. It is pertinent to note that the restorer IL50-13 (IET21943, RPBio4919-50-13-CN2079, IC616879) was released as Chinsurah Nona2 (Gosaba 6) in 2016 for coastal saline areas of West Bengal state of India and notified by central sub-committee on crop standards notification release of varieties in 2019. It gave mean yield 2.8 t/h during 2010-2013 in 4 years multi-location testing of AICRIP (All India Coordinated Rice Improvement Project)^37^. Its yield was similar to that of KMR3 under normal non stress conditions, but in salinity stress IL50-13 showed both seedling stage and reproductive stage tolerance and grain yield was not affected even at 150 mM NaCl^38,39^. Likewise, it also showed drought tolerance under direct seeded conditions^40^. The test hybrid using this restorer line IR79156A/IL50-13 (with *O. rufipogon* introgressions) also showed higher yield than salinity check CSR10 and gave mean grain yield of 5t/h in advanced varietal trial of AICRIP^29^. The genome of IL50-13 and parent line KMR3 were re-sequenced at CCMB (*i*-Life Discoveries Ltd, data information available, seq deposited at DDBJ at DDBJ/ENA/GenBank under accessions LVCG00000000 for KMR3 and LVCH00000000 for IL50-13).

In this study we explored the link between the DEGs within the yield QTLs, their sequence difference and grain yield. Five DEGs Os02t0559800-01 (*E3 ubiquitin ligase EL5*), Os02t0624300-01 (*Similar to Y19 protein*), Os02t0569900-01 (*Cytochrome P450 family protein*) and Os02t0626400-01 (*Phenylalanine ammonia-lyase*), Os02t0626100-01 (*Similar to phenylalanine ammonia-lyase*) within *qyld2.1* and 5 DEGs Os08t0482600-01 (*Cupredoxin domain containing protein*), Os08t0473900-03 (*Similar to alpha-amylase isozyme 3D*), Os08t0473900-01 (*Alpha-amylase isozyme 3D precursor*) and Os08t0466200-01 (*Similar to KUP-related potassium transporter*), Os08t0448000-01 (*Similar to 4 coumarate CoA ligase1*) within *qyld8.2* were validated using qRT-PCR (Supplementary Fig. S5). In addition, 12 other DEGs were also validated and results were consistent with microarray results. The DEGs within *qyld2.1* were mostly down-regulated in flag leaf whereas the DEGs within *qyld8.2* were up-regulated in panicle of test hybrid.

Further, we found that only 19 DEGs out of 136 showed sequence differences between the restorer lines KMR3 and IL50-13. Of the 19 DEGs only 6 showed SNP/InDel variations in CDS region and these 6 DEGs also showed high fold change in test hybrid compared to control hybrid. In accordance with these results the candidate genes within *qyld2.1* were mainly Os02t0626400-03 (*Phenylalanine ammonia-lyase*), Os02t0626400-01 (*Phenyl alanine ammonia-lyase*) and Os02t0627100-01 (*Similar to PAL*). Os02t0626400-03 showing 84 SNPs and 2 InDels, Os02t0626400-01 showing 184 SNPs and 11 InDels and Os02t0627100-01 showing 205 SNPs and 13 InDel variations in CDS regions (Supplementary Table S11). Another DEG within *qyld2.1* Os02t0569900-01 (*Cytochrome P450 family protein*) showed 34 SNPs and 2 InDel variations in CDS region. These 4 DEGs are involved in suberin biosynthesis, phenyl propanoid biosynthesis and initial reactions in salicylate biosynthesis pathways. On the other hand 2 DEGs within *qyld8.2* were Os08t0487301-00 (*Conserved hypothetical protein*) showing 5 SNPs and 2 InDels and Os08t0490100-01 (*Similar to PBF protein*) showing 17 SNPs and 6 InDel variations in CDS regions. In addition, Os08t0509600-01 (*OsSPL14-Squamosa promoter-binding-like transcription factor*) near *qyld8.2* also showed 4 SNP variations in intron regions between KMR3 and IL50-13 but not in exons (Table 4). However, these SNP/InDel variations in introns, UTR regions and CDS regions may not change the function of the final protein, that were involved in metabolic processes, but as modifiers may significantly affect the expression of genes and translation. Alternatively, it is also possible that variations in a promoter sequence acting in trans and away from CDS region confers differential gene expression. But we only considered the genome sequence in the 2 kb upstream and 2 kb downstream of the 136 target genes/DEGs. In general, *OsSPL14* regulates primary panicle branching and vegetative shoot branching. A single point mutation in the micro RNA *OsmiR156*-targeted site in the third exon of *OsSPL14* leads to generation of ideal plant architecture with low tiller number at vegetative stage, lodging resistance and increased grain yield^41,42^.

Interestingly Os06t0248300-03 (*conserved hypothetical protein*, *OsFbox protein* 305) on chromosome 6, Os01t0842500-01 (*Similar to laccase*) on chromosome 1 and Os03t0150800-01 (*Pi transporter, Pi homeostasis, Selenite uptake, OsPT2*) on chromosome 3 were identified as miRNA target genes in test hybrid. Of these, Os06t0248300-03 was down-regulated in panicle and controls anther/pollen development in P3 and P4 stages of rice panicle^43,44^. In addition, 2 pollen-specific cis regulatory elements GTGANTG10 (GTGA) and POLLEN1LeLAT52 (AGAAA) were reported in the regulatory region of *OsFbox* gene that showed maximum activity in meiotic anther stage and controls early anther development in rice^20^. Many *OsFbox protein*-encoding genes control plant growth and various stages of panicle and seed development in rice. Os03t0150800-01 (*OsPht1;2*) the miRNA target gene on chromosome 3 was down-regulated (2.36 fold) in leaf and up-regulated (1.60 fold) in panicle in our study and also reported up-regulated in response to both nitrogen and phosphate starvation in rice roots and shoots^45^. *OsPht1;2* mediates inorganic phosphate (Pi) uptake and transport in root and shoots under Pi deprivation in rice^46^. The third gene *OsLAC5* (Os01t0842500-01, similar to laccase) was up-regulated in panicle and down-regulated in flag leaf. High accumulation of miR397 target laccases that regulate lignification process in wild species *O. nivara* and domestication associated phenotypes such as yield related traits in cultivated rice^47^. The over expression of *OsmiR397* in young panicles and grains enlarges the grain size and promotes panicle branching, leading to an increase in grain yield of up to 25% by down-regulating its target gene *OsLAC* (*laccase like protein*) on chromosome 5^48^. However, the target DEG laccase in our studies is on chromosome 1.

We considered the possible role of the DEGs within yield QTLs in improving yield and abiotic stress tolerance. The DEG *Alpha-amylase* within *qyld8.2* was up-regulated in panicle and plays a major role in starch degradation. These are starvation induced genes which provide energy for elongation of shoot by hydrolysing starch into sugars^49,50^. This was also induced during anoxia as well as stress conditions especially in cold stress in rice shoots^51^. Salinity is another major abiotic stress which affects around 7% of land area of world^52^. In higher plants *similar to KUP related potassium transporters* are up-regulated during K deficiency or salt stress. In our results it was up-regulated in panicle of test hybrid.

Three DEGs (*E3 Ubiquitin ligase*, *Similar to Y19 protein* and *Cytochrome P450*) in *qyld2.1* showed down-regulation in leaf and up-regulation in panicle. Among these sequence difference was found only in Cytochrome P450 and it was in CDS region. *E3 ubiquitin ligases* are candidate genes for yield as they have been reported to enhance yield^53^ and are up-regulated during different abiotic stresses^54–60^. Reduced expression of RING-type E3 ubiquitin ligase on short arm of chromosome 2 in rice increased grain size and weight^53^ and seed size in Arabidopsis^61^. Though *E3 ubiquitin ligase* showed high (4.68) fold change in both flag leaf and panicle of test hybrid but there was no sequence difference between KMR3 and IL50-13. However, it is also likely that trans-acting elements influence its expression. *Os11Gsk* from *O. rufipogon* was previously shown to act in trans and increase yield in IL50-7, a sister line of IL50-13^62^. *Similar to Y19 protein* on chromosome 10 was reported to be induced during drought stress^63,64^. Cytochrome P450 are involved in brassinosteroid pathway and phenyl propanoid pathway and one gene Os04g0469800 of the CytP450 family on chromosome 4 is reported to control panicle structure and seed size^65,66^.

Among the high priority candidate genes within *qyld2.1* which were differentially expressed only in flag leaf was *Phenylalanine ammonia-lyase* (PAL) which also showed sequence difference between KMR3 and IL50-13. PAL is an important enzyme in phenyl propanoid biosynthesis pathway and catalyzes the conversion of L-phenylalanine to cinnamic acid a precursor of salicylic acid which protects plants against various pests and diseases^67^. The phenolic compounds derived during phenyl propanoid biosynthesis pathway scavenge the reactive oxygen species to protect plants against abiotic stresses also^68^. The cytosolic enzymes such as PAL, *4 coumarate CoA ligase* (4CL) and *caffeoyl-CoA 3-O-methyl-transferase* (CCoAOMT) of phenyl propanoid pathway influence cell wall lignin content, and are positively correlated with lodging resistance in culm of buckwheat^69^. Our results showed that *4 coumarate CoA ligases Os4CL2* and *Os4CL3* on chromosome 2 and *Os4CL4* on chromosome 6 were down-regulated in leaf but *Os4CL5* on chromosome 8 was significantly up-regulated in panicle, whereas *caffeoyl-CoA 3-O-methyl-transferase* (CCoAOMT-2) was significantly down-regulated in leaf. Over expression of 4CL (*OsAAE3*) on chromosome 4 reduces floret development, fertility rate of anther, lignin biosynthesis and rice blast resistance in rice^70^. High lignin content decreases cell wall expansion and cell extensibility thereby it can limit water loss and prevent cell collapse during abiotic stress^71^. There is a link between lignin biosynthesis and grain yield as laccase which catalyzes the oxidative polymerization of monolignols into lignin was a DEG in our study. Lignin in plants is synthesised from mono lignols derived from phenylalanine in phenylpropanoid pathway^66^. Laccase is also the target of miRNA397 and a key regulator of domestication phenotype in rice^48^. In our results laccase *OsLAC5* was down-regulated in flag leaf and *OsLAC4*, a microRNA target gene on chromosome 1 was up-regulated in panicle. The PAL genes *OsPAL6* and *OsPAL8* mediate brown planthopper resistance by controlling lignin biosynthesis and its accumulation in rice^72^. Phenylalanine ammonia-lyase (*OsPAL4*) on chromosome 2 is associated with broad spectrum disease resistance in rice. The 750 base pair deletion in second exon of *OsPAL4* in the mutant line leads to down-regulation of *OsPAL4* which up-regulates the expression of *OsPAL2* on chromosome 2 and down-regulates the expression of un-linked *OsPAL6* on chromosome 4^73^. It is significant that in our study the same two genes *OsPAL2* (*Phenylalanine ammonia-lyase*- Os02t0626400-01) and *OsPAL4* (*Similar to phenylalanine ammonia- lyase* - Os02t0627100-01) were down-regulated (2.36 to 2.59) in flag leaf and are thus candidate genes derived from *O. rufipogon* that increase grain yield in test hybrid. Both the genes are located within subQTL-7 region (RM6318-RM1920) of *qyld2.1*^9^. Both expression and sequence analysis indicates that *OsPAL2* and *OsPAL4* influence grain yield in test hybrid. Thus phenyl propanoid pathway and PAL genes in particular are at centre stage of not only broad spectrum disease^74^, pest resistance^72^ and tolerance to abiotic stress^75^ but also for increasing grain yield.

In conclusion, carbohydrate metabolism, phenyl propanoid pathway, ubiquitin-dependent degradation and phytohormone related pathways were most enriched in test hybrid with *O. rufipogon* introgressions compared to control hybrid without *O. rufipogon* introgressions and play a major role in high yield. The yield enhancing role of introgressions from wild species is thus established and key differentially expressed genes underlying major yield enhancing QTL *qyld2.1* identified. Two DEGs *OsPAL2*, (Os02t0626400-01 *Phenyl alanine ammonia-lyase*) and *OsPAL4* (Os02t0627100-01 *Similar to PAL*) within *qyld2.1* showed high SNP and InDel variations in CDS regions and were also differentially expressed in test hybrid. We report for the first time that PAL genes within *qyld2.1* from *Oryza rufipogon* are high priority candidate genes or heterotic loci for higher grain yield in test hybrid.

## Materials and Methods

### Plant material

The seedlings of control hybrid [CMS line IR79156A x restorer line KMR3] and its test hybrid [CMS line IR79156A x restorer line IL50-13] were planted in clay pots (one plant/pot). Please note that in control hybrid the restorer male parent used is without *O. rufipogon* introgression and in test hybrid the restorer male parent used is IL50-13 which is a stable elite backcross introgression line (IL) derived from KMR3 x *O. rufipogon* and thus is with *O. rufipogon* introgression. The same cms line was used as the female parent in both control and test hybrid. Plants were grown in green house at Indian Institute of Rice Research (IIRR). Flag leaves and young panicles at P2 stage (<5 cm long) were collected at panicle initiation stage (Supplementary Fig. S6). The samples were harvested from two biological replicates.

### RNA extraction

Total RNA was extracted from flag leaves and panicles of both control and experimental hybrids (4 samples × 2 biological replications) using Trizol-RNA lysis method (Cat. No. 15596018, Invitrogen, Thermo Fisher Scientific Inc., USA). The extracted RNA was treated with RNase-free DNase I (Qiagen) to prevent genomic DNA contamination. The purity and quantity of the RNA was determined using Nanodrop Spectrophotometer (ND-1000, Thermo Scientific, USA) and integrity of RNA was measured using Agilent 2100 Bioanalyzer (Agilent technologies, Santa Clara, CA95051, USA).

### cDNA labeling

All 8 samples were labeled using Agilent Quick-Amp (single color) labeling kit (p/n5190-0442). 500 ng of each sample RNA was reverse transcribed at 40 °C using oligo dT primer tagged to a T7 polymerase promoter and converted to cDNA. The cRNA was synthesized using cDNA as template by *in vitro* transcription and Agilent dye Cy3 CTP was incorporated during this step. Labeled cRNA was cleaned up using Qiagen RNeasy mini kit columns (Qiagen, Cat No: 74106), quality and quantity was measured using Nanodrop ND-1000.

### Hybridization and scanning

The labeled cRNA samples were fragmented at 60 °C and hybridized on to a genotypic designed rice gene expression microarrays, 8 × 60 k format which contains 60,045 *Oryza sativa* probes (Agilent Technologies, *In situ* hybridization kit, part number 5190-0404, AMADID No: G4102A_048014). Hybridization was carried out in Agilent’s Surehyb Chambers at 65 °C for 16 hours. The hybridized slides were washed using Agilent gene expression wash buffers (Agilent Technologies, Part Number 5188-5327) and scanned using the Agilent Microarray Scanner (Agilent Technologies, Part Number G2600D).

### Statistical analysis

The array data was extracted from images and quantified using feature extraction software version 11.5 and 12.6 Agilent). The raw data was analyzed and normalized with the help of GeneSpring GX software (Agilent technologies, Inc., Santa Clara, CA, 95051, USA) using 75^th^ percentile shift [Percentile shift normalization is a global normalization, where the locations of all the spot intensities in an array are adjusted. This normalization takes each column in an experiment independently, and computes the *n*^th^ percentile of the expression values for this array, across all spots (where *n* has a range from 0–100 and n = 75 is the median). It subtracts this value from the expression value of each entity, and fold expression values were obtained with respect to specific control Samples] (http://genespring-support.com/category/faqcategories/microarray-data-analysis/normalization). Differential expression patterns were identified among the samples. Significant genes up-regulated fold >1 (logbase2) and down-regulated < −1 (logbase2) in the test samples with respect to control sample were identified. Statistical student t-test is used to calculate the p-values among the replicates. Volcano plot was constructed based on p-values from a t-test and fold-change values^76^. Venn diagram was generated to look at the differential expression of common and specific genes in test hybrid compared to control hybrid.

### Functional enrichment analysis

Gene ontology (GO) and KEGG (Kyoto Encyclopedia of Genes and Genomes) pathway analysis was performed to annotate and classify the functional categories and pathways of differentially expressed genes in test hybrid compared to control hybrid. Pathway analysis was performed using Genotypic Biointerpreter-Biological Analysis Software. (Genotypic Technology Private Limited, Bangalore). Genes were also classified based on their functional category, and pathways using Biological Analysis tool DAVID (Database for Annotation, Visualization, and Integrated Discovery) (http://david.abcc.ncifcrf.gov/) (LIB, Frederick, MD, 21702, USA). GO annotations for DEGs were plotted using WEGO 2.0 (Web Gene Ontology Annotation Plot) online tool^77^. Pathway analysis was also performed for some of the important pathways within the major yield QTL *qyld2.1* and minor QTL *qyld8.2* regions using Mapman tool^78^. Wilcoxon Rank Sum Test was used to calculate the *P*-values for significant pathways in test hybrid.

### Primer designing

Few differentially expressed genes underlying the major yield QTL *qyld2.1* and minor QTL *qyld8.2* were selected for real time PCR validation. In addition, several other yield related genes were selected based on their role in different metabolic pathways. Gene sequences were retrieved from NCBI (http://www.ncbi.nlm.nih.gov). Primers for quantitative polymerase chain reaction (qPCR) (Table 1) were designed using Primer 3 software (http://frodo.wi.mit.edu/).

### qRT-PCR validation of DEGs

First-strand cDNA was synthesized from 2 μg of total RNA in a 25 μl reaction mixture with M-MLV reverse transcriptase (Promega, Madison, WI, USA) and oligo dT primers. cDNA was treated with RNase and normalized to obtain similar concentration. qRT-PCR was performed on a ABI 7500 real-time analyzer (Applied Biosystems), using a SYBR premix ExTaq kit (Takara Bio). The levels of *OsActin1* served to normalize the expression ratio for each gene. Each reaction was run in duplicate (with three biological replicates) and the melting curves were constructed using Dissociation Curves Software (Applied Biosystems), to ensure that only a single specific product is amplified.

Changes in expression were calculated via the ‘comparative Ct method’ (Applied Biosystems). The mean threshold cycle (Ct) value obtained after each reaction was normalized to the Ct value of reference gene whose expression was consistent across the conditions. Further ΔΔCT values were calculated using the formulae ΔΔCT = ΔCT of test sample − ΔCT control sample, and then fold difference was calculated from 2^−ΔΔCt^. Similarly, ΔCT standard deviation was calculated as given at www3.appliedbiosystems.com/…/general documents/cms_042380.pdf.

### Sequence comparison of DEGs and other reported yield related genes for polymorphism

In order to identify polymorphism between KMR3 and IL50-13, we performed BLASTN analysis of 136 DEGs including 78 DEGs of *qyld2.1*, 25 DEGs of *qyld8.2*, 12 DEGs showing high fold expression and 21 reported yield related gene sequences against the scaffolds obtained by whole-genome sequencing of the restorers KMR3 (without *O. rufipogon*) and IL50-13 [IET21943 = RPBio4919-50-13] (with *O. rufipogon*). The comparison was done by considering the gene sequence corresponding to Nipponbare and its gene sequence was used to obtain the corresponding gene sequences from the scaffolds of the two restorers KMR3 and IL50-13. The orthologous genomic regions were available for all genes analysed. Further we also analyzed the effect of identified SNPs/InDels in the coding region that could affect the expression of the genes showing polymorphism and also the effect of the variants in the 2 kb upstream and downstream regions of the given genes using the SnpEff software (Wayne State University, Detroit MI USA)^79^. The DEGs along with their gene and chromosome names and their corresponding coordinates are given in Supplementary Table S3.

### Ethics statement

The authors declare that the experiments comply with the current laws of the country in which they were performed and in compliance with ethical standards.

## Supplementary information

Supplementary Information.

Supplementary Information 2.

Supplementary Information 3.

Supplementary Information 4.

Supplementary Information 5.

Supplementary Information 6.

Supplementary Information 7.

Supplementary Information 8.

Supplementary Information 9.

Supplementary Information10.

## Data Availability

All data generated or analysed during this study are included in this published article (and its Supplementary Information files). The sequence data is available on request.
