# Supplementary material for: Genome-wide transcriptome profile of rice hybrids with and without Oryza rufipogon introgression reveals candidate genes for yield
Source: Sci Rep. 2020 Mar 17;10:4873. doi: 10.1038/s41598-020-60922-6 (PMC7078188; doi:10.1038/s41598-020-60922-6)
Supplement: Supplementary file 10 — Supplementary Information10. [file 41598_2020_60922_MOESM10_ESM.pdf]

Genome-wide transcriptome profile of rice hybrids with and without *Oryza rufipogon* introgression reveals candidate genes for yield

G. Haritha[1], T. Shashi Rekha[2], A. Surekha [1], S. K. Mangrauthia[1], R. Rajeshwari[2], N. Sarla[1]*

[1]*ICAR-Indian Institute of Rice Research, Hyderabad 500 030, India*
[2]*CSIR-Centre for Cellular and Molecular Biology, Hyderabad 500 007, India*

***Corresponding author**

N. Sarla

ICAR-National Professor

Biotechnology

ICAR - Indian Institute of Rice Research (IIRR)

Rajendranagar, Hyderabad-500 030, India.

Tel.: +91 40 24591225

Fax: +91 40 24591217

E-mail: sarla_neelamraju@yahoo.com

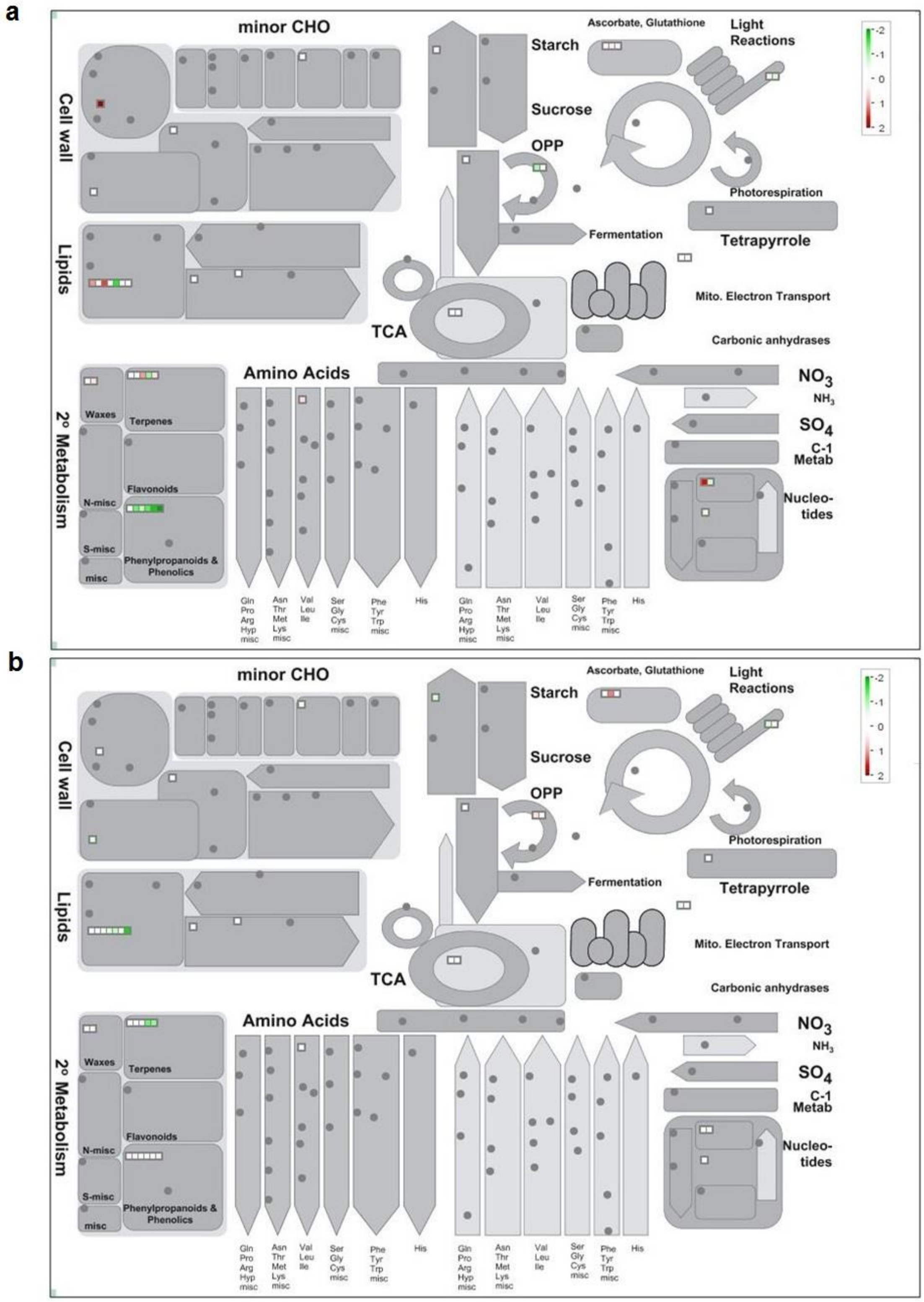

Figure S1. Metabolism overview of the differentially expressed genes within the yield QTL *qyld2.1* of test hybrid (a) Mapman overview of flag leaf (b) Mapman overview of panicle

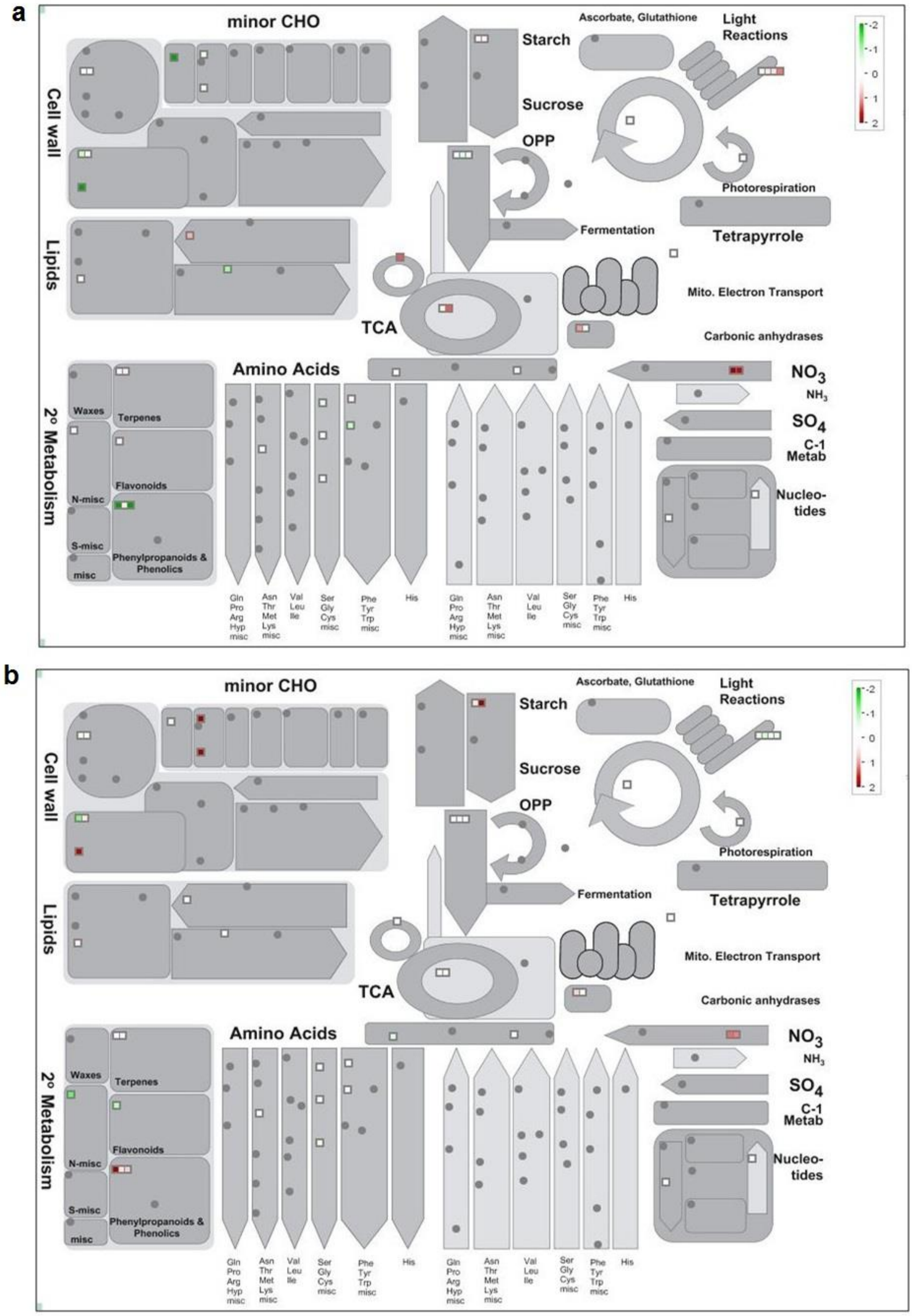

Figure S2. Metabolism overview of the differentially expressed genes within the yield QTL *qyld8.2* of test hybrid on chromosome 8 (a) Mapman overview of flag leaf (b) Mapman overview of panicle

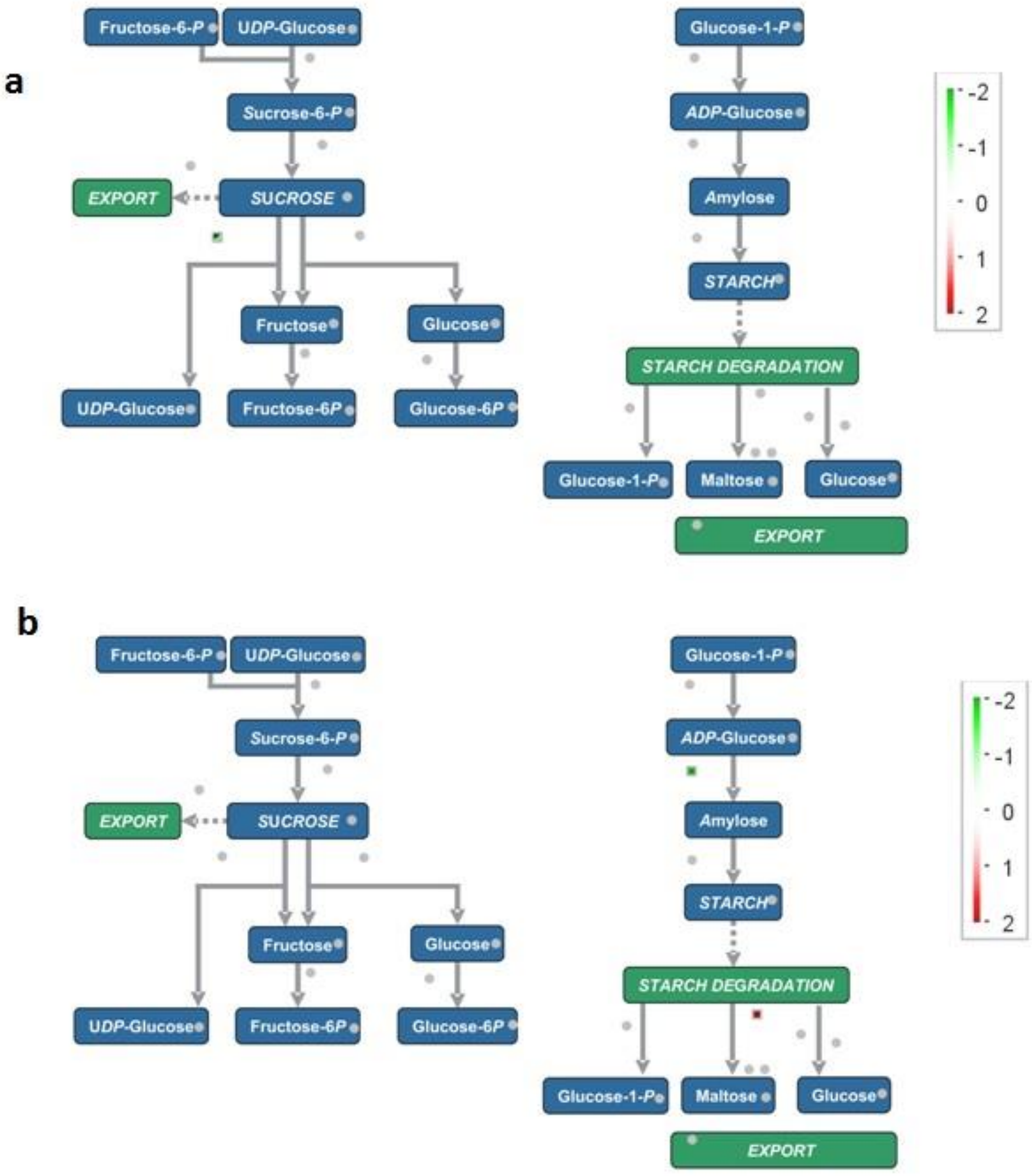

Figure S3. Major carbohydrate metabolism involves sucrose/starch degradation (a) flag leaf (b) panicle. The green squares represent the down- regulation and red squares represent the up-regulation of differentially expressed genes in test hybrid.

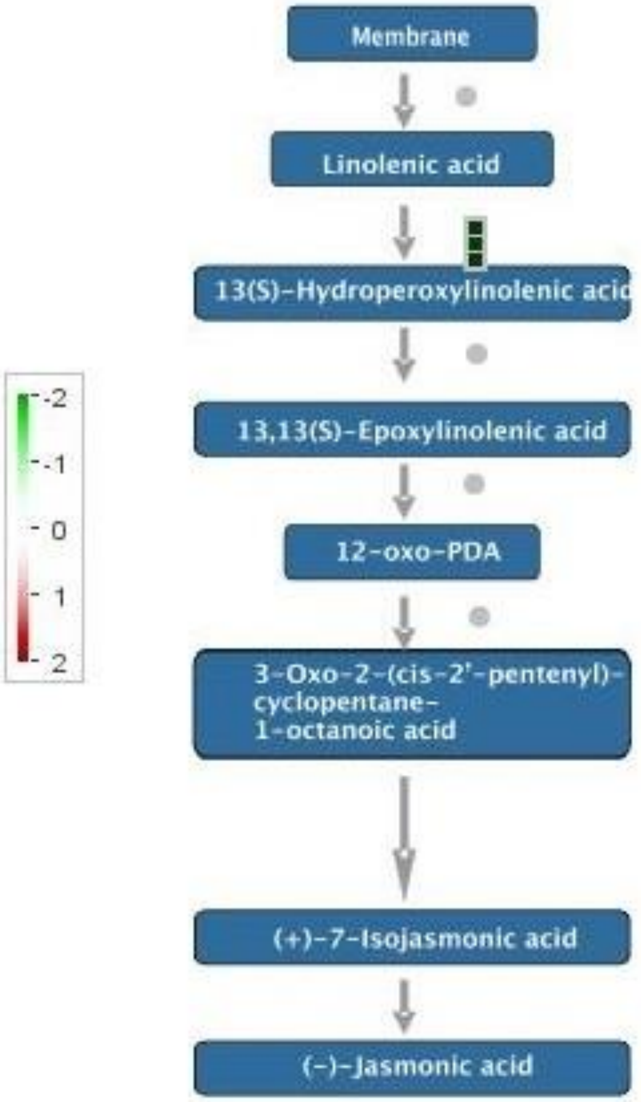

Figure S4. Bio synthesis of jasmonic acid (JA) from α-linolenic acid. The green squares represent the down regulation in flag leaf of test hybrid.

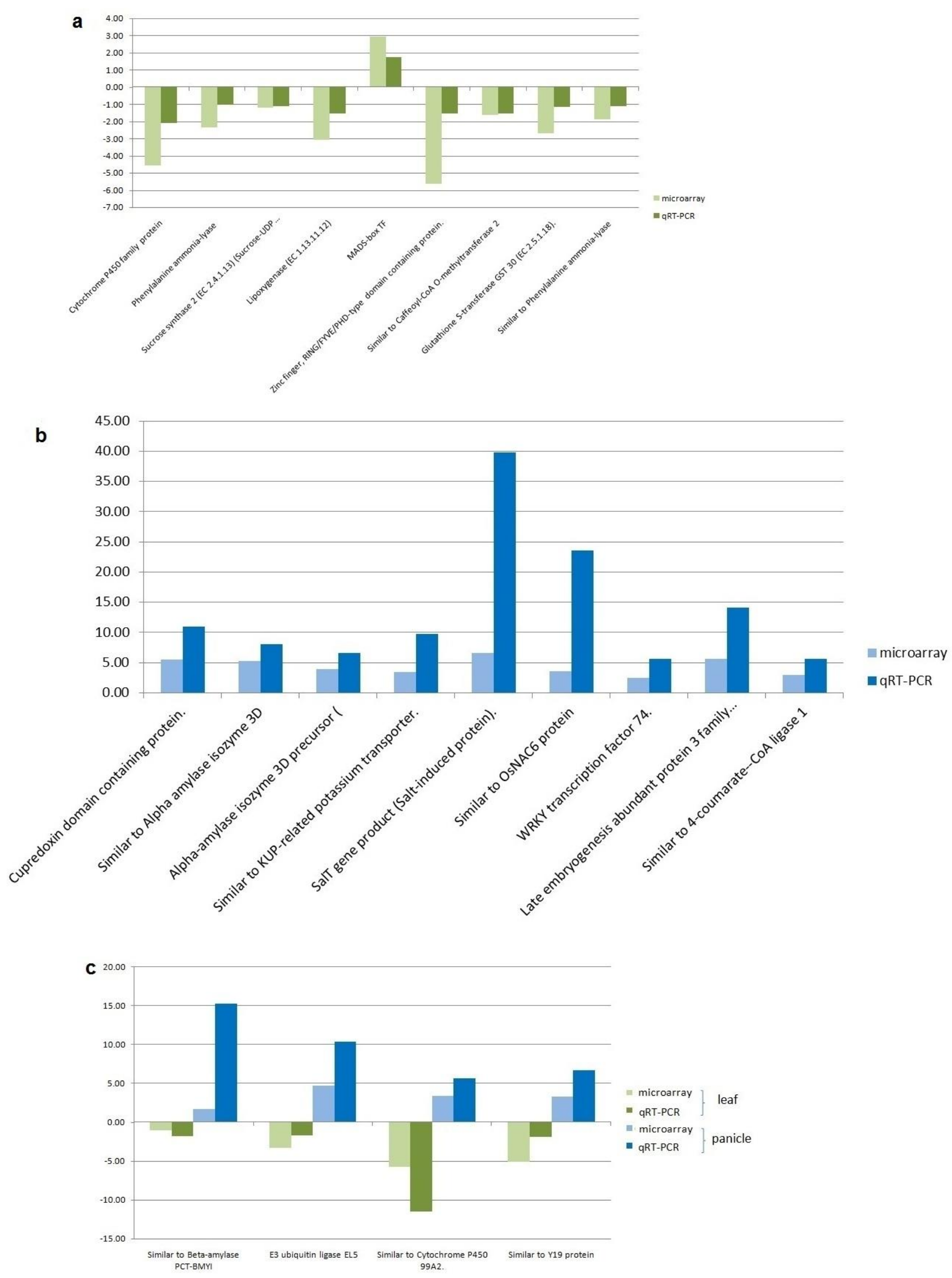

Figure S5. Expression profiles of DEGs in test hybrid as identified by microarray data were measured by qRT-PCR. The results of differences in gene expression from microarray and qRT-PCR were found to be consistent.

(a) qRT-PCR validation of differential expression of genes in flag leaf

(b) qRT-PCR validation of differential expression of genes in panicle

(c) qRT-PCR validation of differential expression of genes in both flag leaf and panicle

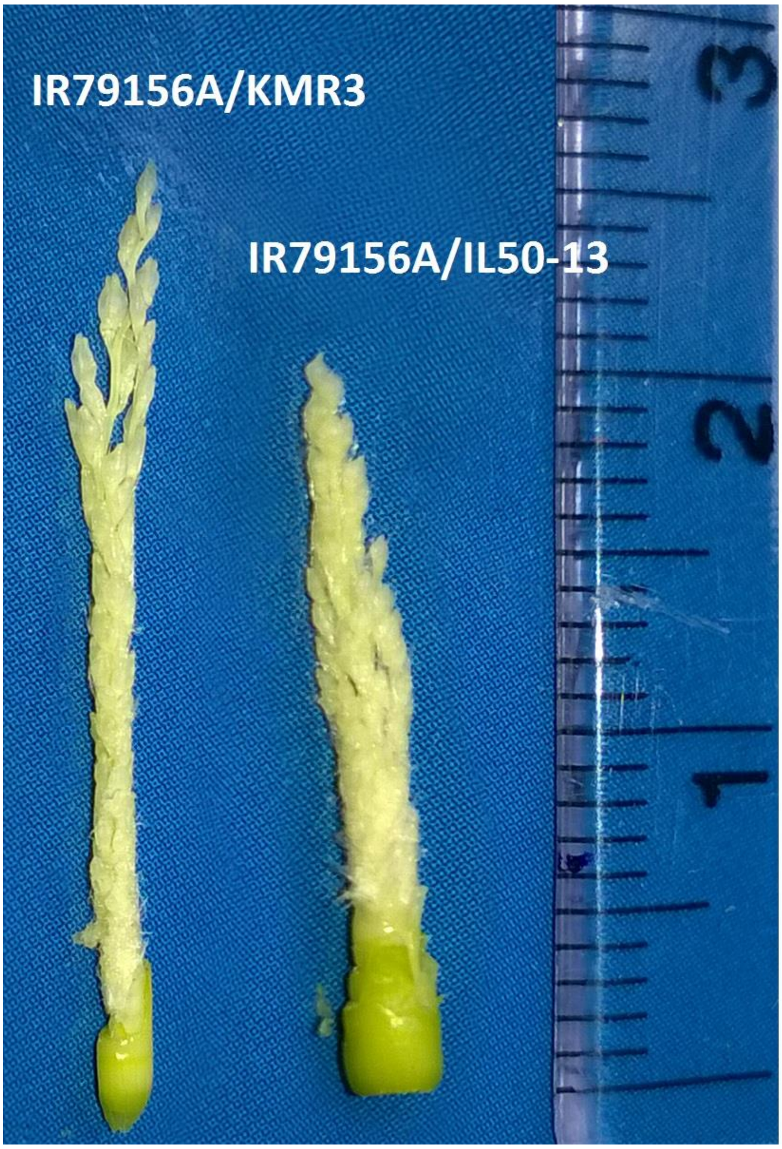

Figure S6. Young stage panicle <5cm in length used for transcriptome analysis

Supplementary figure legends

Figure S1. Metabolism overview of the differentially expressed genes within the yield QTL *qyld2.1* of test hybrid (a) Mapman overview of flag leaf (b) Mapman overview of panicle

Figure S2. Metabolism overview of the differentially expressed genes within the yield QTL *qyld8.2* of test hybrid on chromosome 8 (a) Mapman overview of flag leaf (b) Mapman overview of panicle

Figure S3. Major carbohydrate metabolism involves sucrose/starch degradation (a) flag leaf (b) panicle. The green squares represent the down- regulation and red squares represent the up-regulation of differentially expressed genes in test hybrid.

Figure S4. Bio synthesis of jasmonic acid (JA) from α-linolenic acid. The green squares represent the down regulation in flag leaf of test hybrid.

Figure S5. Expression profiles of DEGs in test hybrid as identified by microarray data were measured by qRT-PCR. The results of differences in gene expression from microarray and qRT-PCR were found to be consistent.

(a) qRT-PCR validation of differential expression of genes in flag leaf

(b) qRT-PCR validation of differential expression of genes in panicle

(c) qRT-PCR validation of differential expression of genes in both flag leaf and panicle

Figure S6. Young stage panicle <5cm in length used for transcriptome analysis

Supplementary Tables

Table S3. DEGs and reported yield related genes considered for analysis to study the differences in the gene sequences between KMR3 and IL50-13. Category A refers to genes within *qyld2.1*, B refers to genes within *qyld8.2*, C refers to genes showing high fold expression and D refers to reported yield related genes

S. No.	Category	Gene Name	Chromosome	Start	End	Strand
1	A1	Os02t0552500-00	chr02	20836392	20838251	+
2	A2	Os02t0552700-01	chr02	20846435	20847993	+
3	A3	Os02t0555300-01	chr02	20972825	20973948	+
4	A4	Os02t0555600-02	chr02	20989673	20994224	+
5	A5	Os02t0559800-01	chr02	21250083	21251343	-
6	A6	Os02t0562600-01	chr02	21330472	21335235	-
7	A7	Os02t0563000-00	chr02	21365479	21366789	+
8	A8	Os02t0565600-01	chr02	21487610	21489894	-
9	A9	Os02t0568600-00	chr02	21625022	21625725	+
10	A10	Os02t0569400-02	chr02	21690803	21692185	+
11	A11	Os02t0569800-00	chr02	21734265	21738288	+
12	A12	Os02t0569900-01	chr02	21740304	21741960	+
13	A13	Os02t0570700-01	chr02	21810375	21813457	-
14	A14	Os02t0571800-00	chr02	21886504	21897686	-
15	A15	Os02t0572300-00	chr02	21922917	21926203	-
16	A16	Os02t0574800-01	chr02	22057873	22058611	+
17	A17	Os02t0575000-01	chr02	22064966	22065592	-
18	A18	Os02t0576600-01	chr02	22146429	22150147	-
19	A19	Os02t0578333-00	chr02	22232078	22232549	+
20	A20	Os02t0579600-00	chr02	22294657	22301807	+
21	A21	Os02t0579800-01	chr02	22311834	22313012	-
22	A22	Os02t0580300-02	chr02	22333316	22337414	+
23	A23	Os02t0580900-02	chr02	22369985	22371481	-
24	A24	Os02t0581400-02	chr02	22421879	22426149	+
25	A25	Os02t0583700-00	chr02	22509550	22510620	-
26	A26	Os02t0583900-01	chr02	22514865	22515484	-
27	A27	Os02t0587300-00	chr02	22636061	22636963	+
28	A28	Os02t0589000-01	chr02	22722799	22733189	-
29	A29	Os02t0590400-02	chr02	22805689	22808343	-
30	A30	Os02t0590400-01	chr02	22805733	22808493	-
31	A31	Os02t0591000-01	chr02	22836027	22838425	+
32	A32	Os02t0592000-00	chr02	22900707	22910523	-
33	A33	Os02t0593600-01	chr02	22978830	22981452	+
34	A34	Os02t0594800-01	chr02	23062855	23066544	-
35	A35	Os02t0595100-01	chr02	23083106	23086437	-
36	A36	Os02t0596200-01	chr02	23142111	23144293	+
37	A37	Os02t0596300-01	chr02	23152884	23154628	-

38	A38	Os02t0600500-00	chr02	23495000	23495287	+
39	A39	Os02t0603700-00	chr02	23651854	23653184	+
40	A40	Os02t0604500-00	chr02	23683321	23684394	+
41	A41	Os02t0605000-01	chr02	23710890	23712446	+
42	A42	Os02t0606700-01	chr02	23769400	23772793	+
43	A43	Os02t0608050-00	chr02	23847120	23847452	+
44	A44	Os02t0609000-01	chr02	23900438	23900923	-
45	A45	Os02t0611800-01	chr02	24080704	24084615	+
46	A46	Os02t0612700-01	chr02	24133183	24139373	-
47	A47	Os02t0613900-02	chr02	24223305	24228713	+
48	A48	Os02t0613950-00	chr02	24224668	24224973	-
49	A49	Os02t0616100-01	chr02	24372806	24374394	+
50	A50	Os02t0616450-01	chr02	24391737	24395742	+
51	A51	Os02t0616600-01	chr02	24410357	24414084	-
52	A52	Os02t0617400-01	chr02	24516374	24518168	+
53	A53	Os02t0618400-02	chr02	24580457	24582527	-
54	A54	Os02t0618700-01	chr02	24595175	24596966	+
55	A55	Os02t0619166-00	chr02	24630717	24631478	-
56	A56	Os02t0620600-01	chr02	24690884	24692736	+
57	A57	Os02t0620900-00	chr02	24700974	24701678	-
58	A58	Os02t0623300-01	chr02	24819271	24820066	+
59	A59	Os02t0624300-01	chr02	24878777	24879932	-
60	A60	Os02t0626100-01	chr02	24973450	24977287	-
61	A61	Os02t0626400-03	chr02	24985256	24986844	-
62	A62	Os02t0626400-01	chr02	24985294	24989388	-
63	A63	Os02t0627100-01	chr02	25006495	25009018	-
64	A64	Os02t0629000-01	chr02	25153298	25154265	-
65	A65	Os02t0629400-01	chr02	25176174	25179701	-
66	A66	Os02t0629900-01	chr02	25191799	25193194	-
67	A67	Os02t0630100-00	chr02	25194429	25196895	-
68	A68	Os02t0630300-02	chr02	25199505	25202158	-
69	A69	Os02t0631700-01	chr02	25286967	25287842	-
70	A70	Os02t0632900-00	chr02	25356937	25360011	-
71	A71	Os02t0635600-01	chr02	25488084	25491732	+
72	A72	Os02t0638650-01	chr02	25595575	25597607	-
73	A73	Os02t0639800-05	chr02	25671552	25673590	-
74	A74	Os02t0639800-02	chr02	25671614	25677225	-
75	A75	Os02t0639800-04	chr02	25672275	25675323	-
76	A76	Os02t0640300-01	chr02	25696949	25698399	-
77	A77	Os02t0640401-00	chr02	25698500	25698889	+
78	A78	Os02t0643800-01	chr02	25878905	25879875	+
79	B1	Os08t0458532-00	chr08	22477005	22479169	-
80	B2	Os08t0459700-00	chr08	22553089	22554026	-
81	B3	Os08t0462900-01	chr08	22715246	22716410	-
82	B4	Os08t0466200-01	chr08	22904756	22908287	+
83	B5	Os08t0467100-01	chr08	22971625	22974903	+
84	B6	Os08t0468100-01	chr08	23033230	23038506	+

85	B7	Os08t0468100-02	chr08	23033973	23038463	+
86	B8	Os08t0468100-03	chr08	23036533	23038585	+
87	B9	Os08t0469300-00	chr08	23075194	23075610	+
88	B10	Os08t0473900-01	chr08	23341289	23343273	+
89	B11	Os08t0473900-03	chr08	23341357	23343240	+
90	B12	Os08t0474000-01	chr08	23353856	23354936	+
91	B13	Os08t0474866-00	chr08	23403049	23404168	-
92	B14	Os08t0476300-02	chr08	23464000	23468392	+
93	B15	Os08t0476600-00	chr08	23475471	23476235	+
94	B16	Os08t0478000-01	chr08	23575567	23576307	+
95	B17	Os08t0481000-02	chr08	23755951	23763739	-
96	B18	Os08t0482600-01	chr08	23853339	23854314	-
97	B19	Os08t0483400-00	chr08	23881968	23882372	+
98	B20	Os08t0485800-01	chr08	24020865	24022693	-
99	B21	Os08t0487301-00	chr08	24109853	24110337	+
100	B22	Os08t0489300-03	chr08	24186673	24189314	+
101	B23	Os08t0490100-01	chr08	24232676	24233936	+
102	B24	Os08t0490400-00	chr08	24245038	24245636	+
103	B25	Os08t0491300-00	chr08	24279068	24280669	-
104	C1	Os01t0314800-01	chr01	11863682	11864412	-
105	C2	Os01t0348900-01	chr01	13903285	13904626	-
106	C3	Os01t0348900-02	chr01	13903285	13904626	
107	C4	Os02t0831500-01	chr02	35754946	35761216	+
108	C5	Os03t0401300-01	chr03	16301279	16306089	-
109	C6	Os03t0401300-02	chr03	16301374	16302930	-
110	C7	Os03t0753100-01	chr03	31048351	31055017	-
111	C8	Os03t0815100-01	chr03	34166100	34167521	+
112	C9	Os04t0180400-01	chr04	5484865	5486703	+
113	C10	Os06t0229800-01	chr06	6748398	6753302	+
114	C11	Os09t0334500-01	chr09	10128837	10131086	+
115	C12	Os10t0531400-01	chr10	20645931	20646880	+
116	D1	Os01t0831000-01	chr01	35558148	35559225	+
117	D2	Os01t0746400-00	chr01	31225458	31228566	-
118	D3	Os01t0197700-01	chr01	5270449	5275585	-
119	D4	Os01t0879500-01	chr01	38157319	38159782	+
120	D5	Os02t0244100-01	chr02	8115223	8121651	+
121	D6	Os03t0407400-00	chr03	16729501	16735109	-
122	D7	Os03t0753100-01	chr03	31048351	31055017	-
123	D8	Os03t0117900-01	chr03	978816	979770	+
124	D9	Os04t0413500-01	chr04	20422171	20426921	+
125	D10	Os04t0615000-01	chr04	31212165	31214739	-
126	D11	Os04t0396500-00	chr04	19563794	19576451	-
127	D12	Os05t0158500-01	chr05	3439304	3443769	-
128	D13	Os06t0677000-00	chr06	28109236	28111005	-
129	D14	Os06t0665400-01	chr06	27480082	27481453	-
130	D15	Os06t0610300-01	chr06	24312470	24313095	+
131	D16	Os07t0616000-01	chr07	25381698	25389532	+

132	D17	Os07t0261200-01	chr07	9152377	9155030	-
133	D18	Os07t0669500-01	chr07	28299602	28301089	-
134	D19	Os08t0509600-01	chr08	25274541	25278696	-
135	D20	Os09t0441900-01	chr09	16411151	16415851	+
136	D21	Os11t0235200-01	chr11	7193230	7198486	-

Table S11. Sequences of the 19 genes showing differences between KMR3 and IL 50-13.

5'UTR sequences are highlighted in blue gray color.
CDS (exon) sequences are highlighted in yellow color.
3'UTR sequences are highlighted in orange color.
SNPs are highlighted in light cyan color and InDels are highlighted in light green color.

S.No: 1

A28: Os02t0589000-01

Query: KMR3: scaffold9446_size10065

Subject: IL50-13: scaffold14856_size6220

```
Score             Expect      Identities        Gaps         Strand
1502 bits(813)    0.0         831/844(98%)      3/844(0%)    Plus/Plus


Query  1757   TTTCAAATAATACACTATTTTAAAATAAAGTTTACACA---ANNNNNNNNNNATATCAAA  1813
              ||||||||||||||||||||||||||||||||||||||   |           ||||||||
Sbjct  1      TTTCAAATAATACACTATTTTAAAATAAAGTTTACACAATTATATTGTGTACATATCAAA  60


Query  1814   TAAACAATTTAAGATCTATATTAACTAATTTATACGTGTACTTAAACAAATGGACCCATT  1873
              ||||||||||||||||||||||||||||||||||||||||||||||||||||||||||||
Sbjct  61     TAAACAATTTAAGATCTATATTAACTAATTTATACGTGTACTTAAACAAATGGACCCATT  120


Query  1874   ATTTAAATTATCGGATCTATTTTTTATTACTTTGGACTATTATTCTCAAATAATGCATTG  1933
              ||||||||||||||||||||||||||||||||||||||||||||||||||||||||||||
Sbjct  121    ATTTAAATTATCGGATCTATTTTTTATTACTTTGGACTATTATTCTCAAATAATGCATTG  180


Query  1934   TTTTAAAATAAAGTCTACACCATCATATCGTGTACCCATTAAATTAACAATTTAAatata  1993
              ||||||||||||||||||||||||||||||||||||||||||||||||||||||||||||
Sbjct  181    TTTTAAAATAAAGTCTACACCATCATATCGTGTACCCATTAAATTAACAATTTAAATATA  240


Query  1994   tatatatatatatatataAAACTAATTATATTTACACTAGAACTGGTGGACCCATTATTT  2053
              ||||||||||||||||||||||||||||||||||||||||||||||||||||||||||||
Sbjct  241    TATATATATATATATATAAAACTAATTATATTTACACTAGAACTGGTGGACCCATTATTT  300


Query  2054   AAATCAACAGATCTATTGTTAATAACTAAAGTGTTCAAAGTTCTCTTTTTGCCCGCTACA  2113
              ||||||||||||||||||||||||||||||||||||||||||||||||||||||||||||
Sbjct  301    AAATCAACAGATCTATTGTTAATAACTAAAGTGTTCAAAGTTCTCTTTTTGCCCGCTACA  360


Query  2114   CAATACACATTTGCACCTTTGCCTTTCGCACGTGCGACAAAGCATCGTGTCTGAGGCATC  2173
              ||||||||||||||||||||||||||||||||||||||||||||||||||||||||||||
Sbjct  361    CAATACACATTTGCACCTTTGCCTTTCGCACGTGCGACAAAGCATCGTGTCTGAGGCATC  420


Query  2174   TTAGGTGATCACGACCGCTGCAACGCTACCGCTTTTGTCAATAACATGAAAATAAAATTA  2233
```

```
                ||||||||||||||||||||||||||||||||||||||||||||||||||||||||||||
Sbjct  421     TTAGGTGATCACGACCGCTGCAACGCTACCGCTTTTGTCAATAACATGAAAATAAAATTA  480


Query  2234    AAGGTGTGTTGCAGCGAGAGAAGAATTAAGCACTATAGGAACACTTAGCTCTGTTTAAGC  2293
                ||||||||||||||||||||||||||||||||||||||||||||||||||||||||||||
Sbjct  481     AAGGTGTGTTGCAGCGAGAGAAGAATTAAGCACTATAGGAACACTTAGCTCTGTTTAAGC  540

Query  2294    TGAAATCGAGAGATGATTACATAATAATTATAATGTATTGTGTTATGTTGTATTTGGATT  2353
                ||||||||||||||||||||||||||||||||||||||||||||||||||||||||||||
Sbjct  541     TGAAATCGAGAGATGATTACATAATAATTATAATGTATTGTGTTATGTTGTATTTGGATT  600


Query  2354    TTGTATTATTTGTTCGTACAGTTGAAAGAAATCATTATGATATAAATTTGAGTACAATAT  2413
                ||||||||||||||||||||||||||||||||||||||||||||||||||||||||||||
Sbjct  601     TTGTATTATTTGTTCGTACAGTTGAAAGAAATCATTATGATATAAATTTGAGTACAATAT  660


Query  2414    TAACATATATTAATAGAATTCTTAATAGGACAAGAGTGTTATAAGGGAGTTTAAGGCTCT  2473
                ||||||||||||||||||||||||||||||||||||||||||||||||||||||||||||
Sbjct  661     TAACATATATTAATAGAATTCTTAATAGGACAAGAGTGTTATAAGGGAGTTTAAGGCTCT  720


Query  2474    ATTCTTTTGGATTTTCGTTTGCACGCTCCACGCTTTCCAAGTTCCTAAATGGTGAttttt  2533
                ||||||||||||||||||||||||||||||||||||||||||||||||||||||||||||
Sbjct  721     ATTCTTTTGGATTTTCGTTTGCACGCTCCACGCTTTCCAAGTTCCTAAATGGTGATTTTT  780


Query  2534    ttaaacaactttttaactttttAGAAGTTAATTCATTCGTTTATAATCTCAAATAGCTAT  2593
                ||||||||||||||||||||||||||||||||||||||||||||||||||||||||||||
Sbjct  781     TTAAACAACTTTTTAACTTTTTAGAAGTTAATTCATTCGTTTATAATCTCAAATAGCTAT  840


Query  2594    CACA  2597
                ||||
Sbjct  841     CACA  844
```

Table of SNPs and InDels in Os02t0589000-01

	cds	5' UTR	3'UTR	Introns
No. of variants:13	0	0	0	13
No. of SNPs: 10	0	0	0	10
No.of InDels: 3	0	0	0	3

S.No: 2

A61: Os02t0626400-03

Query: KMR3: scaffold13385_size7025

Subject: IL50-13: scaffold12144_size7524

```
Score              Expect      Identities       Gaps        Strand
1862 bits(1008)    0.0         1181/1267(93%)   2/1267(0%)  Plus/Minus

Query  1     AAGCCGGAGTACACCGACCACCTCACCCACAAGCTGAAGCACCACCCGGGGCAGATCGAG  60
             ||||||||||||||||||||||| ||||||||||||||||||||||| || |||||||||
Sbjct  1266  AAGCCGGAGTACACCGACCACCTGACCCACAAGCTGAAGCACCACCCTGGACAGATCGAG  1207

Query  61    GCGGCCGCCATCATGGAGCACATCTTGGAAGGCAGCTCGTACATGAAGGAGGCGAAGAGG  120
             || || ||||||||||||||||||||| ||||||||||||||||||||  |||||||| |
Sbjct  1206  GCTGCTGCCATCATGGAGCACATCTTGCAAGGCAGCTCGTACATGAAGCTGGCGAAGAAG  1147

Query  121   CTCGGCGAGCTCGACCCGCTGATGAAGCCGAAGCAGGACAGGTACGCGCTCCGGACGTCG  180
             ||||| |||||||||||| |||||||||||||||| ||| ||||||||||||| ||||||
Sbjct  1146  CTCGGTGAGCTCGACCCGTTGATGAAGCCGAAGCAAGACCGGTACGCGCTCCGCACGTCG  1087

Query  181   CCGCAGTGGCTCGGCCCCCAGATCGAGGTCATCCGCGCCGCCACAAAGTCGATCGAGCGC  240
             ||||| ||||| ||||| |||||||| || || || || ||||| ||||| || ||||||
Sbjct  1086  CCGCAATGGCTTGGCCCTCAGATCGAAGTTATTCGTGCGGCCACCAAGTCCATTGAGCGC  1027

Query  241   GAGATCAACTCCGTCAACGACAACCCGCTCATCGACGTCTCCCGCGGGAAGGCGCTGCAC  300
             ||||||||||| ||||| |||||||| ||||| || ||||||||||  |||||||| || 
Sbjct  1026  GAGATCAACTCTGTCAATGACAACCCACTCATTGATGTCTCCCGCGACAAGGCGCTTCAT  967

Query  301   GGCGGCAACTTCCAGGGCACCCCCATCGGCGTGTCCATGGACAACACCCGCCTCGCCATC  360
             || || |||||||||||||| |||||||| ||||||||||||||||| |||||||| || 
Sbjct  966   GGTGGTAACTTCCAGGGCACTCCCATCGGTGTGTCCATGGACAACACTCGCCTCGCAATT  907

Query  361   GCCGCCATCGGGAAGCTCATGTTCGCGCAGTTCTCCGAGCTGGTGAACGACTTCTACAAC  420
             || |||||||| ||||||||||||||||||||||| ||||| |||||||||| |||||||
Sbjct  906   GCTGCCATCGGCAAGCTCATGTTCGCGCAGTTCTCAGAGCTTGTGAACGACTACTACAAC  847

Query  421   AACGGCCTGCCCTCCAACCTCTCCGGCGGCCGCAACCCGAGCTTGGACTACGGCTTCAAG  480
             |||||||| ||||||||||| |||||||| ||||||||||||||||| ||||||||||||
Sbjct  846   AACGGCCTTCCCTCCAACCTGTCCGGCGGGCGCAACCCGAGCTTGGATTACGGCTTCAAG  787

Query  481   GGCGCCGAGATCGCCATGGCGTCCTACTGCTCCGAGCTGCAGTTCTTGGGCAACCCGGTG  540
             |||||||||||||||||||| || |||||||||||||||||||||||||| ||||| |||
Sbjct  786   GGCGCCGAGATCGCCATGGCTTCGTACTGCTCCGAGCTGCAGTTCTTGGGAAACCCAGTG  727
```

```
Query  541   ACCAACCACGTCCAGAGCGCCGAGCAGCACAACCAGGACGTGAACTCGCTGGGCCTCATC  600
             |||||||||||||||||||| |||||||||||||||||||| ||||| || |||||||||
Sbjct  726   ACCAACCACGTCCAGAGCGCGGAGCAGCACAACCAGGACGTCAACTCCCTCGGCCTCATC  667

Query  601   TCCTCCAGGAAGACGGCGGAGGCCATCGACATCCTCAAGCTCATGTCCTCCACCTTCCTC  660
             |||||||||||||| || |||||| |||| || || ||||||||||||||||| ||| | 
Sbjct  666   TCCTCCAGGAAGACAGCCGAGGCCGTCGAAATACTGAAGCTCATGTCCTCCACGTTCTTG  607

Query  661   GTCGCGCTCTGCCAGGCGATCGACCTGCGCCACATCGAGGAGAACGTCAAGGGCGCCGTC  720
             ||||| || ||||||||| |||||||||| ||||||||||||||||||||||||||||||
Sbjct  606   GTCGCCCTGTGCCAGGCCATCGACCTGCGGCACATCGAGGAGAACGTCAAGGGCGCCGTC  547

Query  721   AAGACCTGCGTCATGACGGTCGCCAAGAAGACGCTGAGCACCAACTCCACCGGCGGCCTC  780
             |||| |||||||||||| || |||| |||||| |||||||| | | ||||||||||||||
Sbjct  546   AAGAGCTGCGTCATGACAGTGGCCAGGAAGACTCTGAGCACGAGCGCCACCGGCGGCCTC  487

Query  781   CACGTCGCCCGCTTCTGCGAGAAGGACCTGCTC-AGCGAGATCGACCGGGAGGCCGTGTT  839
             |||| ||| ||||||||||||||||| |||||| || |||||||||||| || ||||||||
Sbjct  486   CACGCCGCTCGCTTCTGCGAGAAGGATCTGCTCCAG-GAGATCGACCGCGAAGCCGTGTT  428

Query  840   CGCGTACGCCGACGACCCGTGCAGCGCCAACTACCCGCTGATGAAGAAGCTCCGCAGCGT  899
             ||||||||||||||||||||||||||||||||||||||||||||||||||||||||||||
Sbjct  427   CGCGTACGCCGACGACCCGTGCAGCGCCAACTACCCGCTGATGAAGAAGCTCCGCAGCGT  368

Query  900   GCTCGTCGAGCGCGCGCTCGCCAACGGCGCCGCCGAGTTCGACGCGGAGACCTCCGTGCT  959
             ||||||||||||||||||||||||||||||||||||||||||||||||||||||||||||
Sbjct  367   GCTCGTCGAGCGCGCGCTCGCCAACGGCGCCGCCGAGTTCGACGCGGAGACCTCCGTGCT  308

Query  960   CGCCAAGGTCGCGCGGTTCGAGGAGGAGCTCCGCGCGGCGCTGCCCCGGGCCGTCGAGGC  1019
             ||||||||||||||||||||||||||||||||||||||||||||||||||||||||||||
Sbjct  307   CGCCAAGGTCGCGCGGTTCGAGGAGGAGCTCCGCGCGGCGCTGCCCCGGGCCGTCGAGGC  248

Query  1020  CGCGCGCGCCGCCGTGGAGAGCGGCACGGCGGCGGCGCCCAACAGGATCGCCGAGTGCCG  1079
             ||||||||||||||||||||||||||||||||||||||||||||||||||||||||||||
Sbjct  247   CGCGCGCGCCGCCGTGGAGAGCGGCACGGCGGCGGCGCCCAACAGGATCGCCGAGTGCCG  188

Query  1080  CTCGTACCCGCTCTACCGGTTCGTGCGCGAGGAGCTCGGCACCGCCTACCTCACCGGCGA  1139
             ||||||||||||||||||||||||||||||||||||||||||||||||||||||||||||
Sbjct  187   CTCGTACCCGCTCTACCGGTTCGTGCGCGAGGAGCTCGGCACCGCCTACCTCACCGGCGA  128

Query  1140  GAAGACGCGGTCGCCCGGCGAGGAGCTCAACAAGGTGCTCGTCGCGATCAACCAGGGCAA  1199
             ||||||||||||||||||||||||||||||||||||||||||||||||||||||||||||
Sbjct  127   GAAGACGCGGTCGCCCGGCGAGGAGCTCAACAAGGTGCTCGTCGCGATCAACCAGGGCAA  68

Query  1200  GCACATCGACCCGCTGCTCGAGTGCCTCAAGGAGTGGAACGGCGCGCCACTGCCAATCTG  1259
             ||||||||||||||||||||||||||||||||||||||||||||||||||||||||||||
Sbjct  67    GCACATCGACCCGCTGCTCGAGTGCCTCAAGGAGTGGAACGGCGCGCCACTGCCAATCTG  8

Query  1260  CTGAACA  1266
             |||||||
Sbjct  7     CTGAACA  1
```

Table of SNPs and InDels in Os02t0626400-03

	cds	5' UTR	3'UTR	Introns
No. of variants:86	86	0	0	0
No. of SNPs: 84	84	0	0	0
No.of InDels: 2	2	0	0	0

S.No: 3

A11: Os02t0569800-00

Query: KMR3: scaffold2303_size25555

Subject: IL50-13: scaffold17631_size5156

Score	Expect	Identities	Gaps	Strand
2510 bits(1359)	0.0	1594/1712(93%)	18/1712(1%)	Plus/Plus

```
Query  255   ATAAAATATTTTCAAGAACAAGATGAGCTAGCAGCTAATCATTTTGTTAAAATGGACATG  314
             ||||  ||||| |||||||||||||||| ||||||||||||||||||||||   || |||
Sbjct  10    ATAATCTATTTACAAGAACAAGATGAGCAAGCAGCTAATCATTTTGTTAAA---GAGATG  66

Query  315   CAGAGATTCTTTATTTTTTGTGTGGATATGAATTGAATGAAGGTAATATTAATATCTTAT  374
             |||||||||||||||||||||||||||||||||||||||| |||||||||||||||||||
Sbjct  67    CAGAGATTCTTTATTTTTTGTGTGGATATGAATTGAATGAGGGTAATATTAATATCTTAT  126

Query  375   TGATGTTCCTAAAAATGTAGTGCGCAATTGTCGTACAAATGAAAACATTTTTTGATATGA  434
             ||||||||| ||||||||||||| ||||||| ||||||||||| |||||||||||||| |
Sbjct  127   TGATGTTCCCAAAAATGTAGTGCACAATTGTTGTACAAATGAATACATTTTTTGATATAA  186

Query  435   GGCGGCATGGTGTGGAGGCGTAGGTCAACATGAGTACAATAGTCTGCCCACTATATGTTC  494
             |||||||||||||||||||||||||||||||||| |||||||||||||||||||||||||
Sbjct  187   GGCGGCATGGTGTGGAGGCGTAGGTCAACATGAGAACAATAGTCTGCCCACTATATGTTC  246

Query  495   ATTATAGTGTTTTGCCTTGATAAGAAATGTTGACCATGCTGACCATTTATTGCTTTTTGC  554
             ||||||||||||||| ||||||||| || ||||||||||||||||||||||||||||| |
Sbjct  247   ATTATAGTGTTTTGCTTTGATAAGAGATTTTGACCATGCTGACCATTTATTGCTTTTTAC  306

Query  555   AGGCATCTGATTTCTCGGTGATCAAGGATGGAGGTGAGGAACTTCAACGAGAAGCTGGTT  614
             ||||||||||||||||||| |||||| |||||||||||||||||| |||||||||||||||
Sbjct  307   AGGCATCTGATTTCTCGGTAATCAAGTATGGAGGTGAGGAACTTTAACGAGAAGCTGGTT  366

Query  615   GAATAAAATTTCTACCTTATTACTGAGGTACATGGCCATACATTGCTATGTCGATCGTTA  674
             |||||||||||||||||||||||||||||||||  |||||||||||||||||||| ||||
Sbjct  367   GAATAAAATTTCTACCTTATTACTGAGGTACATTTCCATACATTGCTATGTCGATAGTTA  426

Query  675   TTAGTGAAGGGGGTGCTACAAGCACTTTGTTTTCTTCTTTGTGATGAATTTGATATTGTT  734
             ||||||||||||||||||||||||||||||||||||||||||||||||||||||||||||
Sbjct  427   TTAGTGAAGGGGGTGCTACAAGCACTTTGTTTTCTTCTTTGTGATGAATTTGATATTGTT  486

Query  735   CCCCCCAATACTTAGGTAAACAAGGGCGTGTACTTGTGCTTCTTCTAAAACTTAATGAAT  794
             |   |||||||||||||||||||||||||||||||||||||||||| |||||||||||||
Sbjct  487   CT--CCAATACTTAGGTAAACAAGGGCGTGTACTTGTGCTTCTTCTGAAACTTAATGAAT  544
```

```
Query  795   GGCTGCCTCCACTGCTTGTGGTaaaaaaaCTCTTAGTTGTTGCTTTATaaaaaaaaaaaT  854
             |||||||||||||||||| |||||||||||||||||||||||||||||||||   |||||
Sbjct  545   GGCTGCCTCCACTGCTTGAGGTAAAAAAACTCTTAGTTGTTGCTTTATAAAAT--AAAAT  602

Query  855   CTATGTTGCCATCGATTCTTTGCTGTCATGGTGAAAGGGCCAGCTAGCAAGAACAACACT  914
             ||||||||||||| |||| ||||| ||| |||||||||||| |||| |||||||||||||
Sbjct  603   CTATGTTGCCATCAATTC-TTGCTATCACGGTGAAAGGGCCGGCTATCAAGAACAACACT  661

Query  915   TACATAGATGTATTATACTCAAGATCCTTTCGTGATTGTTTGAAAATTATATTCATGGTT  974
             |||||||||||| ||||||||||||||||| |||||||||||||||||||||||||||||
Sbjct  662   TACATAGATGTACTATACTCAAGATCCTTTTGTGATTGTTTGAAAATTATATTCATGGTT  721

Query  975   ATTATTTGACTTGATACTTATTTTCATGGTTTTAAACACATGCACTAAAATCAGCTTGGA  1034
             ||||||| ||||||| ||||||||||||||||||||||||||||||||||||||||||||
Sbjct  722   ATTATTTAACTTGATTCTTATTTTCATGGTTTTAAACACATGCACTAAAATCAGCTTGGA  781

Query  1035  GGAATGGGATATAAGCAGGAGTTTATGTATCCAAAATATGTAATCAGACTCTGGGATATG  1094
             | ||||||||||||||||||||||||||||||||||||| |||||||||| |||||||||
Sbjct  782   GTAATGGGATATAAGCAGGAGTTTATGTATCCAAAATATTTAATCAGACTGTGGGATATG  841

Query  1095  CTGTAAATTTGAGCTGGTCTGCCTCTGTTAATGGGTTAAAAGTCATGGATGCTGGTGTTA  1154
             |||||||||||||||||||| |||||||| ||||||||||||||||||||||||||||||
Sbjct  842   CTGTAAATTTGAGCTGGTCTACCTCTGTTCATGGGTTAAAAGTCATGGATGCTGGTGTTA  901

Query  1155  G-CCAGATGAAATAGCATCATTGCGCTCTACTGTATCAGGA--TTCCCTTGAATCCTGGA  1211
             | ||||||||||||||||||||||||||||||||||| |||  |||||||||||||||||
Sbjct  902   GCCCAGATGAAATAGCATCATTGCGCTCTACTGTATCGGGATTTTCCCTTGAATCCTGGA  961

Query  1212  TTTGCTATTTTGTTTTGCTTCCCTCAAACAATTGTATCTGATGTTATAATCTGATATATC  1271
             ||||||||||||||||||||||||||||||||||| || |||||||||||||||||||||
Sbjct  962   TTTGCTATTTTGTTTTGCTTCCCTCAAACAATTGTGTCCGATGTTATAATCTGATATATC  1021

Query  1272  ATAGTTTGA-ACGTGATACACAGGTATAGTATAGTATAACCTGCAGTGCCAGATTTGGAC  1330
             |||||||   |||||||||||||||||||||||  |    ||||||||||||||||||||
Sbjct  1022  ATAGTTTATCACGTGATACACAGGTATAGTATA--AG---CTGCAGTGCCAGATTTGGAC  1076

Query  1331  ATCTTCAGTTATGGGACTTGATAAAGCTTGGAGATCATCTCGGTGAACGGGAGGATGTTG  1390
             |||||||||| |||||||||||||||||||||||||||||||||||||||||||||||||
Sbjct  1077  ATCTTCAGTTGTGGGACTTGATAAAGCTTGGAGATCATCTCGGTGAACGGGAGGATGTTG  1136

Query  1391  ATCCATGCAGTTGTGTGTTCACCTGTGTTCTGTTATACAACTAATATGCAGTGATAGATG  1450
             |||            ||||||||||||||||||||||||||||||||||| |||||||||
Sbjct  1137  ATCNNNNNNNNNNA-TGTTCACCTGTGTTCTGTTATACAACTAATATGCAATGATAGATG  1195

Query  1451  CACTATGGTATATCATCGTCAGATCTTCCTTCAAAGTGGATGGTAGCTATCTGTGCAGAT  1510
             |||| | |||||||||||||||||||| |||||||| |||||| ||||||||||||||||
Sbjct  1196  CACTGTAGTATATCATCGTCAGATCTTACTTCAAAGAGGATGGGAGCTATCTGTGCAGAT  1255

Query  1511  AGGTTGACAAAAAATTGCACGCCCATGTTCAGATTTCAGAATCAATACATGTGCAAGTTT  1570
             ||||||||||||||||||| ||||||||||||||||||||||||||||||||||||||||
Sbjct  1256  AGGTTGACAAAAAATTGCATGCCCATGTTCAGATTTCAGAATCAATACATGTGCAAGTTT  1315
```

```
Query  1571  CAGACTATTAAAGTTCAGAGCGATTTATGCATGATATCACACATTACTAGCTTAATTTAT  1630
              ||||||||||||| |||| |  |||||| |||| |||||||||||||||||||||||||
Sbjct  1316  AAGACTATTAAAGTCCAGAACATTTTATGTATGACATCACACATTACTAGCTTAATTTAT  1375

Query  1631  CTCAAAACGCAGGCCCGCACAAGCCCAAGTTTTAGCTCCAAACCATAAAACTGCAAAATT  1690
             ||||||||||||||| ||||||||||||||||||| ||||||||||||||||||||||||
Sbjct  1376  CTCAAAACGCAGGCCTGCACAAGCCCAAGTTTTAGATCCAAACCATAAAACTGCAAAATT  1435

Query  1691  CTGTTTGCCATACAATCTGACTATATTCTAATTGCATATTTCTATAATTCGCTCACTAA  1750
              ||||||||||||||||||||||||||||||||||||| |||||||||||||||| | ||
Sbjct  1436  TTGTTTGCCATACAATCTGACTATATTCTAATTGCATACTTTCTATAATTCGCTCCCCAA  1495

Query  1751  ATGTAGTCTATTATCAATTTTTAAATTCATTGTATATGTTTTGCAATTTAGATTGTCCAA  1810
             | | | |||||||| |||  |||||| ||||||||||| ||||||||||||||||| |||
Sbjct  1496  AGGCAATCTATTATTAATCATTAAATCCATTGTATATGCTTTGCAATTTAGATTGTACAA  1555

Query  1811  TTCATAGTGTTATAGTTGATTGATATCATTTGAATGTTTTTAGAGTAACTATTATTGTAT  1870
             |||||||||| |||||||||||| |||||||||||||||| |||||||||||||||||| 
Sbjct  1556  TTCATAGTGTCATAGTTGATTGACATCATTTGAATGTTTTGAGAGTAACTATTATTGTAA  1615

Query  1871  TCACAAATACATTTATAGAACAATAATGCATAAGTTTATTACGTTTTCACGTTATTTAAG  1930
             ||||||||| ||||| ||||||||| |||| |||||||||| ||||||| ||||||||||
Sbjct  1616  TCACAAATATATTTACAGAACAATATTGCACAAGTTTATTATGTTTTCATGTTATTTAAG  1675

Query  1931  ACCTGTTGCAACGCACGGGCATCGGACTAGTA  1962
             ||| |||||||||||||||||||  |||||||
Sbjct  1676  ACCCGTTGCAACGCACGGGCATCATACTAGTA  1707
```

Table of SNPs and InDels in Os02t0569800-00

	cds	5' UTR	3'UTR	Introns
No. of variants:118	4	0	0	114
No. of SNPs: 100	4	0	0	96
No.of InDels: 18	0	0	0	18

S.No: 4

A51: Os02t0616600-01

Query: KMR3: scaffold15492_size5752

Subject: IL5013: scaffold26405_size2842

Score	Expect	Identities	Gaps	Strand
2756 bits(1492)	0.0	1739/1862(93%)	15/1862(0%)	Plus/Plus

```
Query  1     AGTCAAATTTGGACATAAATTCATCTAAATTCAAACTCTTCATTTTGATATCAAATTTGC  60
             ||| ||| |||||| |||||||||||||||||||||||||||||||||||||||||||||
Sbjct  515   AGTGAAACTTGGACCTAAATTCATCTAAATTCAAACTCTTCATTTTGATATCAAATTTGC  574

Query  61    TATTTTAAGATTTTATTTGAATTTCCTTAATTTATTCAAATACCATTTGAATAAATATTT  120
             ||||||||||||||||||||||||||||||||||||||||||||||||||||||||||||
Sbjct  575   TATTTTAAGATTTTATTTGAATTTCCTTAATTTATTCAAATACCATTTGAATAAATATTT  634

Query  121   GATTTAATCTTATGATTGAATTTACAATTTAAACTTATATTTGCTTTCCAAATATTTTCT  180
             ||||||||||||| ||||||||||||||||||||||||||||||||||||||||||||||
Sbjct  635   GATTTAATCTTATAATTGAATTTACAATTTAAACTTATATTTGCTTTCCAAATATTTTCT  694

Query  181   TTTATTTCCAAAATACCCTTTAGCCACTAAAAAGCCGCCGG------CTTTACACCTTCC  234
             |||||||||||||| || |||||||||||||| ||||||||      |||||| ||||||
Sbjct  695   TTTATTTCCAAAATGCCTTTTAGCCACTAAAA-GCCGCCGGTTGGAACTTTACGCCTTCC  753

Query  235   ATGGGGACGTTTGCGCGGTCTAGGGACCTCAACGTCAAACCGTTGGTTGTAAAGCCCGGC  294
             |||||||| || ||| |||||| |||||||||||||  ||||||| ||||||||||||||
Sbjct  754   ATGGGGACATTCGCGTGGTCTACGGACCTCAACGTCGGACCGTTGTTTGTAAAGCCCGGC  813

Query  295   AAAAACCAAACCTAAGGTCGAAGTGCTGCTCCTGGCACCCCACGCCTTGATGCGTACAGC  354
             | |||||||||| |||||||||||||||||||||||| |||| ||||||||||||| |||
Sbjct  814   ATAAACCAAACCCAAGGTCGAAGTGCTGCTCCTGGCATCCCATGCCTTGATGCGTATAGC  873

Query  355   GGTCGGAGCTTCACTATCCCCTCGACCTCCATTGCACGCCGCACAGTTAGCTCTTGAGCT  414
              |||||||||||||||||||||||||||||||||||| |||||||||||||||| | |||
Sbjct  874   AGTCGGAGCTTCACTATCCCCTCGACCTCCATTGCACACCGCACAGTTAGCTCTCGGGCT  933

Query  415   CTGTGTGATCCTAGCCGCTCTGCCGCCACTCACGGTTGACGGAGTAACACTTCCACCCCA  474
             |||||||||||||||||||| |||||||||||| |||| |||||||||||||||||||||
Sbjct  934   CTGTGTGATCCTAGCCGCTCCGCCGCCACTCACAGTTGTCGGAGTAACACTTCCACCCCA  993

Query  475   CTAAAACTGCACCCACCTCCTATTCTTCTAGCCGATATGTTGTCTATCAACACGGCTAGA  534
             ||||| |||||||||||||||||||||||||||||||||||||||||| |||||||||||
Sbjct  994   CTAAACCTGCACCCACCTCCTATTCTTCTAGCCGATATGTTGTCTATCGACACGGCTAGA  1053
```

```
Query  535   AATGATACCAAACCTAGTTGTCATGCTGTCCAACTCTAGCGACGACTAGCGAACACCCAA  594
             |||||||||||||||||| ||||||||  ||||| ||| ||||||||||| |||||| ||
Sbjct  1054  AATGATACCAAACCTAGTCGTCATGCTACCCAACCCTAACGACGACTAGCAAACACCTAA  1113

Query  595   AACCCTAGCCAATATTTATCCTACCTCGATATCGGCCTAAACAACTAAACCCTAGCCACT  654
             |||||||||| ||||||||||||||||||||||||| |||||||||||| ||||||||||
Sbjct  1114  AACCCTAGCCGATATTTATCCTACCTCGATATCGGCTTAAACAACTAAATCCTAGCCACT  1173

Query  655   ATTCTACNNNNNNNNNNNTCAACCTTCACCCATATTAGCCACATGCCTATTTAACCATATC  714
             |||||||           ||||||| ||||||||||||||||||||||||||||||||||
Sbjct  1174  ATTCTACCAAAGAC----CAACCTTGACCCATATTAGCCACATGCCTATTTAACCATATC  1229

Query  715   AACTATCTAGTATATTTCCTATCGTCCAAACCACGAGAGCCTCCACaaaaaaaTATAGAG  774
             ||||||||||||||||||||||| | |||||||||||||||||||||  |||||||||||
Sbjct  1230  AACTATCTAGTATATTTCCTATCATACAAACCACGAGAGCCTCCACAT-AAAATATAGAG  1288

Query  775   CCTAGCCGATATGCTATCCTACTCCACAAACGACTCCCATACAACTAAACCCTAGGTATT  834
             |||| ||| |||||||||||| ||||||| ||||||||||| ||||||||||||| ||||
Sbjct  1289  CCTAACCG-TATGCTATCCTATTCCACAATCGACTCCCATATAACTAAACCCTAGCTATT  1347

Query  835   ATACTACCTAAACCTACCGCTAGTAAATACTAGCCCCATAATCCCTATACCCTATAAAAG  894
             |||| ||||||||||||| |||| ||||||||||||||||||||||||||||||||||||
Sbjct  1348  ATACCACCTAAACCTACCACTAGCAAATACTAGCCCCATAATCCCTATACCCTATAAAAG  1407

Query  895   ATATAGGTTATACCTTAAACCCTAGCCCTTTTAGCCGTTTATCGTTTTCTTACCATTTTA  954
             | ||||||||||||||||||||||||||| |||| |||||||||||||||||  ||||||
Sbjct  1408  AGATAGGTTATACCTTAAACCCTAGCCCTCTTAGTCGTTTATCGTTTTCTTATTATTTTA  1467

Query  955   TGGCTTTTATTGAAATTAGTTCTTCCTCTTTCTCAGTAGAGTCTATTGCCTTTTGGATGG  1014
             |||||||||||||| || |||||||||||||||||||||||| |||||  ||||||||||
Sbjct  1468  TGGCTTTTATTGAATTTGGTTCTTCCTCTTTCTCAGTAGAGTTTATTGTATTTTGGATGG  1527

Query  1015  CAACATATGGTATTTCGAGAATCGGATAATCAGGTTGCCTATCTTGGA-TTATTTGCGTT  1073
             |||| |||||||||||||||||||||||||| |||||||||||||||| |||||||||||
Sbjct  1528  CAACCTATGGTATTTCGAGAATCGGATAATCGGGTTGCCTATCTTGGATTTATTTGCGTT  1587

Query  1074  GGAGCAAGGCAAGTTATTATTCCTC-CCTTTGAGCATAATTATCCTATATTCTACATTCT  1132
             ||||||||||||||||||||||||| ||||||||||||||||||||||||||||||||||
Sbjct  1588  GGAGCAAGGCAAGTTATTATTCCTCTCCTTTGAGCATAATTATCCTATATTCTACATTCT  1647

Query  1133  ATTCTATGCATTAGCCATTTATGATTTTGAATGCATAATCTGAACATATAGCAAACCAGT  1192
             ||||||||||||| ||||| ||||||||||||||||||||||||||||||||||||||||
Sbjct  1648  ATTCTATGCATTAACCATTCATGATTTTGAATGCATAATCTGAACATATAGCAAACCAGT  1707

Query  1193  TATATGCTAGCTTTTGCTTAGCCCTGCCTAGTTAGACTGCATAAAATATAATGAACTTAG  1252
             |||||||||||||||||||||||||||||||||||| |||||||||||||||||||||||
Sbjct  1708  TATATGCTAGCTTTTGCTTAGCCCTGCCTAGTTAGATTGCATAAAATATAATGAACTTAG  1767

Query  1253  ATTGGGACAGTACGTAAGATTTGGTTGATTTGCGGGTGGCTAGTACTGTCTCAGTCGATA  1312
             ||||||||||||||||||||||||||||||| | ||||||||||||| |||| ||| |||
Sbjct  1768  ATTGGGACAGTACGTAAGATTTGGTTGATTTTCAGGTGGCTAGTACTATCTCGGTCAATA  1827
```

```
Query  1313  TAAGTTTACCAAGTGCTTATGTCAAATGAAAGAGTACGACCGCAGCTTCTAGAATGGGGA  1372
             |||||||||||||| ||||||||||| ||| |||||||||| ||||||||||||| ||||
Sbjct  1828  TAAGTTTACCAAGTACTTATGTCAAACGAACGAGTACGACCACAGCTTCTAGAACGGGGA  1887

Query  1373  CAGACCTAGATATTATGTTAATTAACAATGTGGTACCTCTGTACAGTGGTTCTTATGTAA  1432
             ||||||||||||||||||||||||||||||||||||||||||||||||||||||||||||
Sbjct  1888  CAGACCTAGATATTATGTTAATTAACAATGTGGTACCTCTGTACAGTGGTTCTTATGTAA  1947

Query  1433  GTCCTCGCGAAGGAGGCAAATATATGCTGAGCAGGGCAAATGTGATATTTACCGAGGCGC  1492
             ||||||||||||||||||||||||||| | | ||||||||||||||||||||||||||||
Sbjct  1948  GTCCTCGCGAAGGAGGCAAATATATGCCGCGAAGGGCAAATGTGATATTTACCGAGGCGC  2007

Query  1493  ACATAAATATCTTGATTATCACGCGATGTTTGTGTGATTTTATGGAAACCTGGTTGAGAT  1552
             |  ||||||||||||||||| |||||||| ||||||| |||||| |||||||||||||||
Sbjct  2008  AGGTAAATATCTTGATTATCGCGCGATGTGTGTGTGAATTTATGAAAACCTGGTTGAGAT  2067

Query  1553  GACGTGGGGTCTCTCCTCCAGTTCTCTCTAAAAACCTCGGGAACTCCAGAACTCCTCTCG  1612
             ||||||||||||||| ||| |||||||||||||||| ||||||| |||||||||||||||
Sbjct  2068  GACGTGGGGTCTCTCATCCTGTTCTCTCTAAAAACCCCGGGAACGCCAGAACTCCTCTCG  2127

Query  1613  CTGCTGATAACGTTACGTTCAGAGCGCATGGGGACTAGAGTTCATGGAACAGGTGCCACA  1672
             |||||||||||||||| |||| ||||||||||||||||||||||||||||||||| ||||
Sbjct  2128  CTGCTGATAACGTTACATTCAAAGCGCATGGGGACTAGAGTTCATGGAACAGGTGTCACA  2187

Query  1673  ATTGTTAATAACTTAATACTGGGCCATGACTAAGCTAATGATTATTAGCAAGTTATGGAC  1732
             |||||||||||| |||||||||||||||||||||||||| |||||| ||||||  |||||
Sbjct  2188  ATTGTTAATAACCTAATACTGGGCCATGACTAAGCTAATCATTATTGGCAAGTCGTGGAC  2247

Query  1733  TGCGGGTAAAGCGTACATCTACTGCAGTATGAATATGTTTAAAACTATTCGAATAGCCGT  1792
             || |||||||||||||||||||||||||||||||||  |||||||||||||||||| |||
Sbjct  2248  TGTGGGTAAAGCGTACATCTACTGCAGTATGAATATCCTTAAAACTATTCGAATAGTCGT  2307

Query  1793  GCTCACGGATTTGAGCATCGGGACTGATATGGTACTTTGGTTAGAAATATAATTCAAGAT  1852
             ||||||||||||||||||||||||| ||||||||||||||||||||||||||||||||||
Sbjct  2308  GCTCACGGATTTGAGCATCGGGACTCATATGGTACTTTGGTTAGAAATATAATTCAAGAT  2367

Query  1853  TT  1854
             ||
Sbjct  2368  TT  2369
```

Table of SNPs and InDels in Os02t0616600-01

	cds	5' UTR	3'UTR	Introns
No. of variants: 123	0	0	6	117
No. of SNPs: 108	0	0	5	103
No.of InDels: 15	0	0	1	14

S.No: 5

A32: Os02t0592000-00

Query: KMR3: scaffold8341_size11245

Subject: IL5013: scaffold17585_size5169

Score	Expect	Identities	Gaps	Strand
1334 bits(722)	0.0	1194/1424(84%)	35/1424(2%)	Plus/Plus

```
Query  1026  CGCCGACTGGTGTTTTCATCACCATTGATAGACGATTTTATTTCCACATCGGTCACCTCT  1085
             ||||||| |||||||||||   ||||||| ||||| ||| || |||||| || |||||||
Sbjct  1     CGCCGACGGGTGTTTTCATTGGCATTGATGGACGACTTTGTTCCCACATTGGCCACCTCT  60

Query  1086  GTCGTCACCGAGGGAAATATCAAAAAGCTAAGTCATCACAAAttttcttttttatatgat  1145
             | | ||||| || ||||||| | ||||||||||||| |  ||||||||| ||||||||||
Sbjct  61    GCCATCACCAAGAGAAATATTACAAAGCTAAGTCATTATTAATTTTCTTCTTTATATGAT  120

Query  1146  atttttcttttCCTCTGCCTCACTACGTCAACTGGTTCGTCATTGCCTACTGAGTTGGGG  1205
             |||||  |||||||||  | |||||| |||| ||||||  ||||| ||| ||||| ||||
Sbjct  121   ATTTTCTTTTTCCTCTAACCCACTACATCAATTGGTTCACCATTGACTAGTGAGTCGGGG  180

Query  1206  GCTACAGATGTTATATCATAACTTTTCAACAA-TTTTCATAATATTTATCTTGATTGCTC  1264
             |||| ||||||||| |||||  |||| ||||| |||||||||||||||||||||||||| 
Sbjct  181   GCTATAGATGTTATGTCATA-TTTTTTAACAATTTTTCATAATATTTATCTTGATTGCTT  239

Query  1265  GCGACATAATCCGAGTACATAAGTGCCTATCGAGTTATGGAGTTCCATCTTCCTATGCTT  1324
             |||| ||||||  |||||| ||||||||||||||||||||||||| ||||||||||||||
Sbjct  240   GCGATATAATCTAAGTACAAAAGTGCCTATCGAGTTATGGAGTTCTATCTTCCTATGCTT  299

Query  1325  TCTGTTTGGTTTGCCAATGGATAGTTGCTTTTGGGCCCATTCCTAGCACCCGGGGGCTCG  1384
             || ||||||||||||||||||||||||| |||||||| |||||||||||||| |||||||
Sbjct  300   TCCGTTTGGTTTGCCAATGGATAGTTGCCTTTGGGCCAATTCCTAGCACCCGTGGGCTCG  359

Query  1385  TTGGATGGTCCGAATAACGCAAGGTGCTAAGTATTATTCGGAATAAATCAAAAGCATAGA  1444
             |||||||| |||| |||||||||||||||| ||||| ||| |||||||||||||||||||
Sbjct  360   TTGGATGGACCGAGTAACGCAAGGTGCTAAATATTACTCGAAATAAATCAAAAGCATAGA  419

Query  1445  GATAAGATAAATTTATTTTCTGCTCTTTACAATTGCAAAAGTACTCGAGTAGTAAATTCT  1504
             ||| ||||||||||||||||||||||| | ||||||||||||||   |||| ||||||||
Sbjct  420   GATGAGATAAATTTATTTTCTGCTCTTAATAATTGCAAAAGTACCTTAGTAATAAATTCT  479

Query  1505  GATTCGTGAAACTTTGTTCCATTAATGGTGAACTTATGTCACTCATATGCATGTTTGGGA  1564
             ||| |||||||||||||||||||||||  |||||||| ||||||||||||||||||||||
Sbjct  480   GATCCGTGAAACTTTGTTCCATTAATGACGAACTTATATCACTCATATGCATGTTTGGGA  539

Query  1565  AGTGGCTAGGCCGACTCCCGATGCTTGGGGACTATGACTAGTCGGGCAGAGTGTTTAAAC  1624
             |||| |||||| |||||||| ||||||| | ||||||||||| |||||||||||||||||
Sbjct  540   AGTGCCTAGGCTGACTCCCGGTGCTTGGTGGCTATGACTAGTTGGGCAGAGTGTTTAAAC  599
```

```
Query  1625  GTTAGGAGTCATCTGCTCAGGGAAATCAAAATTGACAAGAGGAGAAATACATATTTACAA  1684
             || | ||||||||||||| |||||||||||||||||||||| ||||||||||||||| ||
Sbjct  600   GTAATGAGTCATCTGCTCGGGGAAATCAAAATTGACAAGAGAAGAAATACATATTTATAA  659

Query  1685  ACATTTTAAAATACTTGAATTTTTCTCAAGTACTGTATTTACATCAGATACTACTCATCC  1744
             ||| |||  |||| |||||| |||||||||||||||||||||||||||||||||||||||
Sbjct  660   ACACTTTT-AATATTTGAATCTTTCTCAAGTACTGTATTTACATCAGATACTACTCATCC  718

Query  1745  TATATGTTTGTTCTGCAGGGTCCAGATCCAGATATGCATAATAGGGATTCATGGCTTTAA  1804
             |||||||||||||| |    || ||||  | | || || || |  |||||||||||||||
Sbjct  719   TATATGTTTGTTCTAC----TC-AGAT--ATACAT-CA-AAGA--GATTCATGGCTTTAA  767

Query  1805  GCTTATCTCCTACGCTTCAGGAATGGATTAGTCAGAACATCACCATCAGCTCGCCTTCGT  1864
             |||||||||||| | | ||||||||||| |||||||||||||||| | |||  || ||||
Sbjct  768   GCTTATCTCCTATGTTACAGGAATGGATCAGTCAGAACATCACCACCGGCTTACCGTCGT  827

Query  1865  CGCCGCCGACTAGTGTCTTTGCCTGCACGACTGGCGAATTATGCCGCCGTTGCTGGGCGT  1924
             |||| ||| || ||||| | ||||  |||  |||    | |  ||||||||| |||||||
Sbjct  828   CGCCACCGGCTGGTGTCCTCGCCTATACGGTTGGGTGGTCACACCGCCGTTGTTGGGCGT  887

Query  1925  TTACGTCTTCACCGACCGGCTTGCCTTCAT-CGCCGCCAACTGGTGTCTTTGCCTGCACG  1983
                  || ||||||||||||  |||| | | ||||| | |||||||| || ||||  |||
Sbjct  888   CGTTTTCGTCACCGACCGGCC-GCCT-CGTCCGCCGTCGACTGGTGTTTTCGCCTATACG  945

Query  1984  GCTAGCGTATTATGCCGCCGTTGC------TGGGCGTCTACGTCTTTACCATCGGCTTGC  2037
             | | | || |  |  | |            |||||||||||||||| |||  ||||||||
Sbjct  946   GTT-G-GT-TGGTCACACNNNNNNNNNNATTGGGCGTCTACGTCTTCACCGCCGGCTTGC  1002

Query  2038  CTTTGGTGCCACCGACTGGTGTCCTTGCCTGTACGGCTGGCGTATTATGCCGCCA-TTGT  2096
             ||| |   || |||||| ||||  |  |||  ||||||||| ||||||||||||  ||||
Sbjct  1003  CTTCGTCACCGCCGACTAGTGT-TTCACCTACACGGCTGGCATATTATGCCGCCGTTTGT  1061

Query  2097  TGGGTGTCGACGTCTTCACCGACAGGCTTGTCTCCGCCGCCGCCGACTGGCGTCTCCGCC  2156
             |||| ||  |||||||||||| | |||||| || || |  |||||||||| |||| ||||
Sbjct  1062  TGGGCGTATACGTCTTCACCGCC-GGCTTGCCTTCGTCATCGCCGACTGGTGTCT-CGCC  1119

Query  2157  TGCACGGCTGGCATATTATGCCGCCGTTA-TTGAGCGTCTACATTTTCACCGACCGGCTT  2215
             | ||| ||||||||||||||||||||||  ||| | |||||| | || ||||||||||||
Sbjct  1120  TACACAGCTGGCATATTATGCCGCCGTTTGTTGGGTGTCTACGTCTTTACCGACCGGCTT  1179

Query  2216  GCCTCCGCTGCCGCCGACTGGTGTCTCCGCCAGCACGGCTAGCCTATTATGCTGCCATTG  2275
             |||| ||| |||||||||||||||||||||| |||||||| ||||||||||| |||||||
Sbjct  1180  GCCTTCGCCGCCGCCGACTGGTGTCTCCGCCCGCACGGCTGGCCTATTATGCCGCCATTG  1239

Query  2276  TTGGGCGTCTACGTCTTCACCAACCGGC-CGCCT-CGTCCGCTGCCGACTGGTGTCTCCA  2333
             |||||||||||||||||||||  ||||| ||||| || ||  |||||||||||||||||
Sbjct  1240  TTGGGCGTCTACGTCTTCACCG-CCGGCTCGCCTTCG-CCATTGCCGACTGGTGTCTCCG  1297
```

```
Query  2334  CCTGCACGGCTGGCCTATTATGCCGTTGTTGTTGGACGTCTACATCTTCATCGACCAGCC  2393
             ||| || || ||   ||||||||||  ||||| ||||||||||||||||  ||||| |||
Sbjct  1298  CCTACATGGTTGATTTATTATGCCGCCGTTGTAGGACGTCTACATCTTCTCCGACCGGCC  1357

Query  2394  GCCTCGTATGATGCCAACTGGTGGCTATCGTCTTCACGGCAGGCC  2437
              ||| |||||| ||||||| || |||||| ||||||||| ||||
Sbjct  1358  ACCTTGTATGACGCCAACTTGTACTATCGCCTTCACGGCTGGCC  1401
```

Table of SNPs and InDels in Os02t0592000-00

	cds	5' UTR	3'UTR	Introns
No. of variants: 230	0	0	0	230
No. of SNPs: 195	0	0	0	195
No.of InDels: 35	0	0	0	35

S.No: 6

A16: Os02t0574800-01

Query: KMR3: scaffold14052_size6604

Subject: IL50-13: scaffold21804_size3915

Score	Expect	Identities	Gaps	Strand
455 bits(246)	5e-133	248/249(99%)	0/249(0%)	Plus/Minus

```
Query  25   ggcggcggcgCTGGCTGTCCCGCCGGAGGTGCTGGCCGGCGTGGATGAGGTGGCGCAGGA  84
            ||||||||||||||||||||||| ||||||||||||||||||||||||||||||||||||
Sbjct  249  GGCGGCGGCGCTGGCTGTCCCGCAGGAGGTGCTGGCCGGCGTGGATGAGGTGGCGCAGGA  190

Query  85   CGTCCTGTTCGACCTCATTGGGAGCTATCCGGAAGTGGACGACGTGTTGCACTTCATGAA  144
            ||||||||||||||||||||||||||||||||||||||||||||||||||||||||||||
Sbjct  189  CGTCCTGTTCGACCTCATTGGGAGCTATCCGGAAGTGGACGACGTGTTGCACTTCATGAA  130

Query  145  CGAGTGACGACGTGGCGGGCTTGGCCCCACCACGTGTCGCCGCACGATTGTCCGGGTTTG  204
            ||||||||||||||||||||||||||||||||||||||||||||||||||||||||||||
Sbjct  129  CGAGTGACGACGTGGCGGGCTTGGCCCCACCACGTGTCGCCGCACGATTGTCCGGGTTTG  70

Query  205  GGCGGGAGGCTAAAAGTCGGGTCAGGTTGATGCTGCATTGCTGGCGCTCTAGTGCTGCTA  264
            ||||||||||||||||||||||||||||||||||||||||||||||||||||||||||||
Sbjct  69   GGCGGGAGGCTAAAAGTCGGGTCAGGTTGATGCTGCATTGCTGGCGCTCTAGTGCTGCTA  10

Query  265  GCCTAGCTA  273
            |||||||||
Sbjct  9    GCCTAGCTA  1
```

Table of SNPs and InDels in Os02t0574800-01

		cds	5' UTR	3'UTR	Introns
No. of variants:	1	1	0	0	0
No. of SNPs:	1	1	0	0	0
No.of InDels:	0	0	0	0	0

S.No: 7

A1: Os02t0552500-00

Query: KMR3: scaffold23485_size2774

Subject: IL50-13: scaffold30709_size2103

Score	Expect	Identities	Gaps	Strand
713 bits(386)	0.0	392/395(99%)	0/395(0%)	Plus/Plus

```
Query  1    TACGGCTTAAATGGTTGGCAAAGAGAGGAGAGCATCGCGACAGACCTTGGACAACCATTG  60
            ||||||||||||||||||||||||||||||||||||||||||||||||||||||||||||
Sbjct  21   TACGGCTTAAATGGTTGGCAAAGAGAGGAGAGCATCGCGACAGACCTTGGACAACCATTG  80

Query  61   AGACTAAGCAGCCGAAGGAGTTACAGGACTTGTTCTTCTCAGCCACACGGCAAGTGTTGG  120
            ||||||||||||||||||||||||||||||||||||||||||||||||||||||||||||
Sbjct  81   AGACTAAGCAGCCGAAGGAGTTACAGGACTTGTTCTTCTCAGCCACACGGCAAGTGTTGG  140

Query  121  GAGATGGGGGAAAACACAAAGTTTTGGCTTGCGGATTGGCTGCCAATTGGGAGCATAAAG  180
            ||||||||||||||||||||||||||||||||||||||||||||||||||||| ||||||
Sbjct  141  GAGATGGGGGAAAACACAAAGTTTTGGCTTGCGGATTGGCTGCCAATTGGGAGTATAAAG  200

Query  181  GCGAGATTTCCATTAATCTTCAGTCATGTGGAGCGCAAGGGTTTGACAGTGGCGAGAGGA  240
            ||||||||||| |||||||||| |||||||||||||||||||||||||||||||||||||
Sbjct  201  GCGAGATTTCCGTTAATCTTCAATCATGTGGAGCGCAAGGGTTTGACAGTGGCGAGAGGA  260

Query  241  CTACAGAACCATAGGTGGGTGCGCGACATCAAAGGAGCTCCATCCAACCGAGCCATTGGG  300
            ||||||||||||||||||||||||||||||||||||||||||||||||||||||||||||
Sbjct  261  CTACAGAACCATAGGTGGGTGCGCGACATCAAAGGAGCTCCATCCAACCGAGCCATTGGG  320

Query  301  GAGTACTTTCAAATTTGGGATGCGATCCAGAAGGTGCAGCTGTGAGGAGGGGAAGAGGAT  360
            ||||||||||||||||||||||||||||||||||||||||||||||||||||||||||||
Sbjct  321  GAGTACTTTCAAATTTGGGATGCGATCCAGAAGGTGCAGCTGTGAGGAGGGGAAGAGGAT  380

Query  361  GAGACAATATGGAAGTGGGCCTCGAATGGAAGATT  395
            |||||||||||||||||||||||||||||||||||
Sbjct  381  GAGACAATATGGAAGTGGGCCTCGAATGGAAGATT  415
```

Table of SNPs and InDels in Os02t0552500-00

		cds	5' UTR	3'UTR	Introns
No. of variants:	3	3	0	0	0
No. of SNPs:	3	3	0	0	0
No.of InDels:	0	0	0	0	0

S.No: 8

A12: Os02t0569900-01

Query: KMR3: scaffold7129_size12786

Subject: IL50-13: scaffold24827_size3169

Score	Expect	Identities	Gaps	Strand
220 bits(119)	1e-61	192/228(84%)	2/228(0%)	Plus/Minus

```
Query  1071  GGGAAGGA-GTACGAGTACCTGCCGTTCGGGTCCGGGAGGAGGCTGTGCCCGGGGCTGCC  1129
             |||||||| ||  |||| | |||||||||||||||||||| |||||||||| ||||||||
Sbjct  340   GGGAAGGACGT-GGAGTTCATGCCGTTCGGGTCCGGGAGGCGGCTGTGCCCCGGGCTGCC  282

Query  1130  GCTGGCGGAGCGCGTCGTGCCGTTCGTGCTGGCCTCGCTGCTGCGCGCGTTCGAGTGGCG  1189
             ||||||||||||||||||||||||| | ||||| ||| |||||| | |||||||||||  
Sbjct  281   GCTGGCGGAGCGCGTCGTGCCGTTCATCCTGGCGTCGATGCTGCACACGTTCGAGTGGAA  222

Query  1190  CCTCCCCGATGGCGTGTCCGCCGAGGACCTGGACGTCAGCGAGAGGTTCAACACCGCCAA  1249
              |||||||  ||| || | || |||||| |||||||   ||||| ||||||  | |||||
Sbjct  221   GCTCCCCGGCGGCATGACGGCGGAGGACGTGGACGTGTCCGAGAAGTTCAAGTCTGCCAA  162

Query  1250  TGTGCTCGCCGTGCCTCTCAAGGTCGTGCCTGTCGTCGTCAACTAGTC  1297
             |||||| |||||||||||||||| |||||||||| |  |||| |||||
Sbjct  161   TGTGCTTGCCGTGCCTCTCAAGGCCGTGCCTGTCCTGATCAAATAGTC  114
```

Table of SNPs and InDels in Os02t0569900-01

	cds	5' UTR	3'UTR	Introns
No. of variants:36	36	0	0	0
No. of SNPs: 34	34	0	0	0
No.of InDels: 2	2	0	0	0

S.No: 9

A62: Os02t0626400-01

Query: KMR3: scaffold13385_size7025_2826_4920

Subject:IL50-13: scaffold12144_size7524_2050_4141 Reversed:

Score	Expect	Identities	Gaps	Strand
2780 bits(1505)	0.0	1901/2096(91%)	11/2096(0%)	Plus/Plus

```
Query  1      GCCCCGCGCCGACCCGCTCAACTGGGGGAAGGCGGCGGAGGAGATGGCCGGGAGCCACCT  60
              |||||||||||||||||| ||||||||||||||||||||||||||| | |||||||||||
Sbjct  7      GCCCCGCGCCGACCCGCTTAACTGGGGGAAGGCGGCGGAGGAGATGTCGGGGAGCCACCT  66

Query  61     CGACGAGGTGAAGAGGATGGTGGCGGAGTACCGGCAGCCCGTGGTGCGGATCGAGGGCGC  120
              ||||||||||||| |||||||||| |||||||| |||||  |||||  ||||||||||||
Sbjct  67     CGACGAGGTGAAGCGGATGGTGGCCGAGTACCGCCAGCCGCTGGTGAAGATCGAGGGCGC  126

Query  121    CAGCCTGAGGATCGCGCAGGTGGCGGCGGTTGCCGGCGCCGGCGACGGCGAGGCCGCCAT  180
              ||||||||||||||||||||| || ||||| |  | || ||||  ||||||    |||| 
Sbjct  127    CAGCCTGAGGATCGCGCAGGTCGCCGCGGT-G--GCCG-CGGC--CGGCGA---AGCCAG  177

Query  181    GGTCGAGCTCGACGAGTCGGCGCGGGAGCGCGTCAAGGCCAGCAGCGACTGGGTGATGAA  240
              ||||||||||||||||||||| || |||||||||||||||||||||||||||||||||||
Sbjct  178    GGTCGAGCTCGACGAGTCGGCCCGCGAGCGCGTCAAGGCCAGCAGCGACTGGGTGATGAA  237

Query  241    CAGCATGGCGAACGGCACCGACAGCTACGGCGTCACCACCGGGTTCGGCGCCACGTCGCA  300
              |||||||  ||||||||| |||||||| || ||||||||||| |||||||||||||| ||
Sbjct  238    CAGCATGATGAACGGCACGGACAGCTATGGTGTCACCACCGGCTTCGGCGCCACGTCCCA  297

Query  301    CCGGCGGACCAAGGAGGGCGGCGCGCTGCAGCGGGAGCTCATCAGGTTCCTCAACGCCGG  360
              | || |||||||||| || || || |||||  | |||||||||||||||||||| |||||
Sbjct  298    CAGGAGGACCAAGGAAGGTGGTGCCCTGCAAAGAGAGCTCATCAGGTTCCTCAATGCCGG  357

Query  361    CGCCTTCGGCACCGGCGCCGACGGCCACGTCCTCCCCGCGGGTGCCACGCGCGCGGCGAT  420
              ||||||||||||  || | ||||||||||||||  |||| |  |||||||||||||||||
Sbjct  358    CGCCTTCGGCACGAGCACGGACGGCCACGTCCTGTCCGCCGAGGCCACGCGCGCGGCGAT  417

Query  421    GCTCGTCCGGATCAACACCCTGCTGCAAGGCTACTCCGGCATCCGGTTCGAGATCCTCGA  480
              ||||||||| |||||||| || || || ||||||||||||||||| ||||||||||||||
Sbjct  418    GCTCGTCCGCATCAACACGCTCCTCCAGGGCTACTCCGGCATCCGCTTCGAGATCCTCGA  477

Query  481    GGCGGTCGCCAAGCTGCTCAACGCCAACGTCACGCCGTGCCTGCCGCTCCGGGGCACGAT  540
              |||| |  ||||||||||||| |||||||||||||| |||||||| ||| |||||||| |
Sbjct  478    GGCGATTACCAAGCTGCTCAATGCCAACGTCACGCCATGCCTGCCACTCAGGGGCACGGT  537

Query  541    CACCGCGTCGGGCGACCTCGTCCCGCTCTCCTACATCGCCGGGCTCGTCACCGGCCGGGA  600
               |||||||| |||||||| ||||||||||||||||| |||||  | ||||||||||| ||
Sbjct  538    GACCGCGTCCGGCGACCTGGTCCCGCTCTCCTACATTGCCGGCTTGGTCACCGGCCGCGA  597
```

```
Query  601   GAACTCCGTCGCGGTGGCGCCCGACGGCAGGAAGGTGAACGCCGCCGAGGCGTTCAAGAT  660
             |||||| |||||||| || || || ||||| |||||||||||| | |||||||| |||||
Sbjct  598   GAACTCTGTCGCGGTCGCTCCTGATGGCAGCAAGGTGAACGCCTCTGAGGCGTTTAAGAT  657

Query  661   CGCCGGCATCCAGGGCGGCTTCTTCGAGCTGCAGCCCAAAGAAGGCCTCGCCATGGTGAA  720
              || |||||||| || |||||||||||| |||||||||| ||||| ||||||||||||||
Sbjct  658   TGCTGGCATCCAAGGTGGCTTCTTCGAGTTGCAGCCCAAGGAAGGTCTCGCCATGGTGAA  717

Query  721   CGGGACGGCCGTGGGCTCCGGCCTCGCCTCCACCGTGCTGTTCGAGGCCAACGTCCTCGC  780
              || || ||||| ||||| || || |||||||| ||||| || ||||| ||| | || ||
Sbjct  718   TGGCACTGCCGTCGGCTCTGGACTTGCCTCCACGGTGCTCTTTGAGGCGAACATTCTTGC  777

Query  781   CATCCTCGCCGAGGTGCTCTCCGCCGTGTTCTGCGAGGTGATGAACGGCAAGCCGGAGTA  840
               |||| |||||||| || |||||||||||||||||||||||||||||||||||||||||
Sbjct  778   TGTCCTTGCCGAGGTCCTGTCCGCCGTGTTCTGCGAGGTGATGAACGGCAAGCCGGAGTA  837

Query  841   CACCGACCACCTCACCCACAAGCTGAAGCACCACCCGGGGCAGATCGAGGCGGCCGCCAT  900
             |||||||||||| ||||||||||||||||||||||| || ||||||||||| || |||||
Sbjct  838   CACCGACCACCTGACCCACAAGCTGAAGCACCACCCTGGACAGATCGAGGCTGCTGCCAT  897

Query  901   CATGGAGCACATCTTGGAAGGCAGCTCGTACATGAAGGAGGCGAAGAGGCTCGGCGAGCT  960
             |||||||||||||||| ||||||||||||||||||||  |||||||| |||||| |||||
Sbjct  898   CATGGAGCACATCTTGCAAGGCAGCTCGTACATGAAGCTGGCGAAGAAGCTCGGTGAGCT  957

Query  961   CGACCCGCTGATGAAGCCGAAGCAGGACAGGTACGCGCTCCGGACGTCGCCGCAGTGGCT  1020
             ||||||| |||||||||||||||| ||| ||||||||||||| ||||||||||| |||||
Sbjct  958   CGACCCGTTGATGAAGCCGAAGCAAGACCGGTACGCGCTCCGCACGTCGCCGCAATGGCT  1017

Query  1021  CGGCCCCCAGATCGAGGTCATCCGCGCCGCCACAAAGTCGATCGAGCGCGAGATCAACTC  1080
              ||||| |||||||| || || || || ||||| ||||| || |||||||||||||||||
Sbjct  1018  TGGCCCTCAGATCGAAGTTATTCGTGCGGCCACCAAGTCCATTGAGCGCGAGATCAACTC  1077

Query  1081  CGTCAACGACAACCCGCTCATCGACGTCTCCCGCGGGAAGGCGCTGCACGGCGGCAACTT  1140
              ||||| |||||||| ||||| || ||||||||||  |||||||| || || || |||||
Sbjct  1078  TGTCAATGACAACCCACTCATTGATGTCTCCCGCGACAAGGCGCTTCATGGTGGTAACTT  1137

Query  1141  CCAGGGCACCCCCATCGGCGTGTCCATGGACAACACCCGCCTCGCCATCGCCGCCATCGG  1200
             ||||||||| |||||||| ||||||||||||||||| |||||||| || || ||||||||
Sbjct  1138  CCAGGGCACTCCCATCGGTGTGTCCATGGACAACACTCGCCTCGCAATTGCTGCCATCGG  1197

Query  1201  GAAGCTCATGTTCGCGCAGTTCTCCGAGCTGGTGAACGACTTCTACAACAACGGCCTGCC  1260
              ||||||||||||||||||||||| ||||| |||||||||| ||||||||||||||| ||
Sbjct  1198  CAAGCTCATGTTCGCGCAGTTCTCAGAGCTTGTGAACGACTACTACAACAACGGCCTTCC  1257

Query  1261  CTCCAACCTCTCCGGCGGCCGCAACCCGAGCTTGGACTACGGCTTCAAGGGCGCCGAGAT  1320
             ||||||||| |||||||| ||||||||||||||||| |||||||||||||||||||||||
Sbjct  1258  CTCCAACCTGTCCGGCGGGCGCAACCCGAGCTTGGATTACGGCTTCAAGGGCGCCGAGAT  1317
```

```
Query  1321  CGCCATGGCGTCCTACTGCTCCGAGCTGCAGTTCTTGGGCAACCCGGTGACCAACCACGT  1380
             ||||||||| || |||||||||||||||||||||||||| ||||| ||||||||||||||
Sbjct  1318  CGCCATGGCTTCGTACTGCTCCGAGCTGCAGTTCTTGGGAAACCCAGTGACCAACCACGT  1377

Query  1381  CCAGAGCGCCGAGCAGCACAACCAGGACGTGAACTCGCTGGGCCTCATCTCCTCCAGGAA  1440
             ||||||||| |||||||||||||||||||| ||||| || ||||||||||||||||||||
Sbjct  1378  CCAGAGCGCGGAGCAGCACAACCAGGACGTCAACTCCCTCGGCCTCATCTCCTCCAGGAA  1437

Query  1441  GACGGCGGAGGCCATCGACATCCTCAAGCTCATGTCCTCCACCTTCCTCGTCGCGCTCTG  1500
             ||| || |||||| |||| || || ||||||||||||||||| ||| | ||||| || ||
Sbjct  1438  GACAGCCGAGGCCGTCGAAATACTGAAGCTCATGTCCTCCACGTTCTTGGTCGCCCTGTG  1497

Query  1501  CCAGGCGATCGACCTGCGCCACATCGAGGAGAACGTCAAGGGCGCCGTCAAGACCTGCGT  1560
             |||||| ||||||||||| |||||||||||||||||||||||||||||||||| ||||||
Sbjct  1498  CCAGGCCATCGACCTGCGGCACATCGAGGAGAACGTCAAGGGCGCCGTCAAGAGCTGCGT  1557

Query  1561  CATGACGGTCGCCAAGAAGACGCTGAGCACCAACTCCACCGGCGGCCTCCACGTCGCCCG  1620
             |||||| || |||| |||||| |||||||| | | |||||||||||||||||| ||| ||
Sbjct  1558  CATGACAGTGGCCAGGAAGACTCTGAGCACGAGCGCCACCGGCGGCCTCCACGCCGCTCG  1617

Query  1621  CTTCTGCGAGAAGGACCTGCTC-AGCGAGATCGACCGGGAGGCCGTGTTCGCGTACGCCG  1679
             ||||||||||||||| |||||| || ||||||||||| || |||||||||||||||||||
Sbjct  1618  CTTCTGCGAGAAGGATCTGCTCCAG-GAGATCGACCGCGAAGCCGTGTTCGCGTACGCCG  1676

Query  1680  ACGACCCGTGCAGCGCCAACTACCCGCTGATGAAGAAGCTCCGCAGCGTGCTCGTCGAGC  1739
             ||||||||||||||||||||||||||||||||||||||||||||||||||||||||||||
Sbjct  1677  ACGACCCGTGCAGCGCCAACTACCCGCTGATGAAGAAGCTCCGCAGCGTGCTCGTCGAGC  1736

Query  1740  GCGCGCTCGCCAACGGCGCCGCCGAGTTCGACGCGGAGACCTCCGTGCTCGCCAAGGTCG  1799
             ||||||||||||||||||||||||||||||||||||||||||||||||||||||||||||
Sbjct  1737  GCGCGCTCGCCAACGGCGCCGCCGAGTTCGACGCGGAGACCTCCGTGCTCGCCAAGGTCG  1796

Query  1800  CGCGGTTCGAGGAGGAGCTCCGCGCGGCGCTGCCCCGGGCCGTCGAGGCCGCGCGCGCCG  1859
             ||||||||||||||||||||||||||||||||||||||||||||||||||||||||||||
Sbjct  1797  CGCGGTTCGAGGAGGAGCTCCGCGCGGCGCTGCCCCGGGCCGTCGAGGCCGCGCGCGCCG  1856

Query  1860  CCGTGGAGAGCGGCACGGCGGCGGCGCCCAACAGGATCGCCGAGTGCCGCTCGTACCCGC  1919
             ||||||||||||||||||||||||||||||||||||||||||||||||||||||||||||
Sbjct  1857  CCGTGGAGAGCGGCACGGCGGCGGCGCCCAACAGGATCGCCGAGTGCCGCTCGTACCCGC  1916

Query  1920  TCTACCGGTTCGTGCGCGAGGAGCTCGGCACCGCCTACCTCACCGGCGAGAAGACGCGGT  1979
             ||||||||||||||||||||||||||||||||||||||||||||||||||||||||||||
Sbjct  1917  TCTACCGGTTCGTGCGCGAGGAGCTCGGCACCGCCTACCTCACCGGCGAGAAGACGCGGT  1976

Query  1980  CGCCCGGCGAGGAGCTCAACAAGGTGCTCGTCGCGATCAACCAGGGCAAGCACATCGACC  2039
             ||||||||||||||||||||||||||||||||||||||||||||||||||||||||||||
Sbjct  1977  CGCCCGGCGAGGAGCTCAACAAGGTGCTCGTCGCGATCAACCAGGGCAAGCACATCGACC  2036

Query  2040  CGCTGCTCGAGTGCCTCAAGGAGTGGAACGGCGCGCCACTGCCAATCTGCTGAACA  2095
             ||||||||||||||||||||||||||||||||||||||||||||||||||||||||
Sbjct  2037  CGCTGCTCGAGTGCCTCAAGGAGTGGAACGGCGCGCCACTGCCAATCTGCTGAACA  2092
```

Table of SNPs and InDels in Os02t0626400-01

		cds	5' UTR	3'UTR	Introns
No. of variants:	195	195	0	0	0
No. of SNPs:	184	184	0	0	0
No.of InDels:	11	11	0	0	0

S.No: 10

A63: Os02t0627100-01

Query: KMR3: scaffold7515_size12279_1251_3210

Subject:IL50-13: scaffold12144_size7524_2051_4200

Score	Expect	Identities	Gaps	Strand
2032 bits(1100)	0.0	1533/1751(88%)	13/1751(0%)	Plus/Minus

```
Query  1     CAGATTCCTCAATGCCGGCGCGTTCGGCAGTGGCACCGACGGCCATGTTTGCCGGCAGA  60
             ||| ||||||||||||||||| |||||||   |||| |||||||| ||  || | || ||
Sbjct  1752  CAGGTTCCTCAATGCCGGCGCCTTCGGCACGAGCACGGACGGCCACGTCCTGTCCGCCGA  1693

Query  61    GGCAACCCGCGCGGCAATGCTCGTCCGCATCAACACACTCCTCCAGGGATACTCCGGCAT  120
             ||| || |||||||| |||||||||||||||||||| ||||||||||| |||||||||||
Sbjct  1692  GGCCACGCGCGCGGCGATGCTCGTCCGCATCAACACGCTCCTCCAGGGCTACTCCGGCAT  1633

Query  121   CCGGTTCGAGATCCTCGAGGCGATCGCCAAGCTGCTCAACGCGAACGTCACGCCGTGCCT  180
             ||| ||||||||||||||||||||  ||||||||||||| || ||||||||||| |||||
Sbjct  1632  CCGCTTCGAGATCCTCGAGGCGATTACCAAGCTGCTCAATGCCAACGTCACGCCATGCCT  1573

Query  181   GCCGCTCCGGGGCACGATCACGGCGTCCGGTGACCTCGTCCCGCTGTCCTACATTGCCGG  240
             ||| ||| |||||||| | || |||||||| ||||| |||||||| ||||||||||||||
Sbjct  1572  GCCACTCAGGGGCACGGTGACCGCGTCCGGCGACCTGGTCCCGCTCTCCTACATTGCCGG  1513

Query  241   TCTT-GTCACTGGGCGCGAGAATGCCGTGGCAGTTGCACCAGATGGCAGCAAGGTGAACG  299
              ||| ||||| || ||||||||  | || || || || || |||||||||||||||||||
Sbjct  1512  -CTTGGTCACCGGCCGCGAGAACTCTGTCGCGGTCGCTCCTGATGGCAGCAAGGTGAACG  1454

Query  300   CCGCTGAGGCGTTCAAGATTGCTGGCATCCAGGGCGGCTTCTTCGAGCTGCAGCCCAAGG  359
             || |||||||||| ||||||||||||||||| || |||||||||||| ||||||||||||
Sbjct  1453  CCTCTGAGGCGTTTAAGATTGCTGGCATCCAAGGTGGCTTCTTCGAGTTGCAGCCCAAGG  1394

Query  360   AAGGCCTTGCCATGGTCAATGGCACCGCCGTGGGCTCTGGCCTTGCATCGACCGTGCTCT  419
             |||| || |||||||| |||||||| ||||| |||||||| ||||| || || |||||||
Sbjct  1393  AAGGTCTCGCCATGGTGAATGGCACTGCCGTCGGCTCTGGACTTGCCTCCACGGTGCTCT  1334

Query  420   TTGAGGCCAACATTCTTGCCATCCTCGCCGAGGTCCTCTCGGCCGTGTTCTGCGAGGTGA  479
             ||||||| |||||||||||  |||| ||||||||||| || |||||||||||||||||||
Sbjct  1333  TTGAGGCGAACATTCTTGCTGTCCTTGCCGAGGTCCTGTCCGCCGTGTTCTGCGAGGTGA  1274

Query  480   TGAACGGCAAGCCGGAGTACACCGACCACCTTACTCACAAGCTCAAACACCATCCAGGAC  539
             ||||||||||||||||||||||||||||||| || |||||||| || ||||| || ||||
Sbjct  1273  TGAACGGCAAGCCGGAGTACACCGACCACCTGACCCACAAGCTGAAGCACCACCCTGGAC  1214

Query  540   AGATCGAGGCCGCCGCCATCATGGAGCACATCTTGGAAGGAAGCTCCTACATGAAGCATG  599
             |||||||||| || ||||||||||||||||||||| |||| ||||| ||||||||||  |
Sbjct  1213  AGATCGAGGCTGCTGCCATCATGGAGCACATCTTGCAAGGCAGCTCGTACATGAAGCTGG  1154
```

```
Query  600   CCAAGAAGCTTGGTGAGCTCGACCCACTGATGAAGCCGAAGCAAGACCGGTACGCGCTCC  659
             | |||||||| ||||||||||||||  |||||||||||||||||||||||||||||||||
Sbjct  1153  CGAAGAAGCTCGGTGAGCTCGACCCGTTGATGAAGCCGAAGCAAGACCGGTACGCGCTCC  1094

Query  660   GGACATCCCCACAGTGGCTCGGCCCACAAATTGAGGTCATCCGTGCCGCCACCAAGTCCA  719
             | || || || || ||||| ||||| || || || || || ||||| |||||||||||||
Sbjct  1093  GCACGTCGCCGCAATGGCTTGGCCCTCAGATCGAAGTTATTCGTGCGGCCACCAAGTCCA  1034

Query  720   TCGAGCGTGAGATCAACTCCGTGAACGACAACCCGCTCATCGACGTCTCCCGCGGCAAGG  779
             | ||||| ||||||||||| || || |||||||| ||||| || |||||||||| |||||
Sbjct  1033  TTGAGCGCGAGATCAACTCTGTCAATGACAACCCACTCATTGATGTCTCCCGCGACAAGG  974

Query  780   CGCTGCACGGTGGCAACTTCCAGGGCACGCCCATCGGCGTGTCCATGGA-----------  828
             |||| || ||||| |||||||||||||| |||||||| |||||||||||
Sbjct  973   CGCTTCATGGTGGTAACTTCCAGGGCACTCCCATCGGTGTGTCCATGGACAACACTCGCC  914

Query  829   NNNNNNNNNNTGCCATCGGCAAGCTCATGTTCGCGCAGTTCTCCGAGCTCGTGAACGACT  888
                       ||||||||||||||||||||||||||||||||| ||||| ||||||||||
Sbjct  913   TCGCAATTGCTGCCATCGGCAAGCTCATGTTCGCGCAGTTCTCAGAGCTTGTGAACGACT  854

Query  889   TCTACAACAACGGCCTGCCATCCAACCTGTCTGGCGGTCGCAACCCGAGCTTGGACTACG  948
              ||||||||||||||| || ||||||||||| ||||| ||||||||||||||||| ||||
Sbjct  853   ACTACAACAACGGCCTTCCCTCCAACCTGTCCGGCGGGCGCAACCCGAGCTTGGATTACG  794

Query  949   GGTTCAAGGGCGCCGAGATCGCCATGGCCTCCTACTGCTCCGAGCTGCAGTTCTTGGGCA  1008
             | |||||||||||||||||||||||||| || |||||||||||||||||||||||||| |
Sbjct  793   GCTTCAAGGGCGCCGAGATCGCCATGGCTTCGTACTGCTCCGAGCTGCAGTTCTTGGGAA  734

Query  1009  ACCCAGTGACCAACCACGTCCAGAGCGCCGAGCAGCACAACCAGGACGTCAACTCTCTGG  1068
             |||||||||||||||||||||||||||| |||||||||||||||||||||||||| || |
Sbjct  733   ACCCAGTGACCAACCACGTCCAGAGCGCGGAGCAGCACAACCAGGACGTCAACTCCCTCG  674

Query  1069  GACTCATCTCCTCCAGGAAGACCGCCGAGGCAATCGACATCCTGAAGCTCATGTCCTCCA  1128
             | |||||||||||||||||||| ||||||||  |||| || |||||||||||||||||||
Sbjct  673   GCCTCATCTCCTCCAGGAAGACAGCCGAGGCCGTCGAAATACTGAAGCTCATGTCCTCCA  614

Query  1129  CGTTCCTGATCGCCCTCTGCCAGGCCGTCGACCTGCGGCACATCGAGGAGAACGTCAAGA  1188
             ||||| || ||||||| ||||||||| ||||||||||||||||||||||||||||||||
Sbjct  613   CGTTCTTGGTCGCCCTGTGCCAGGCCATCGACCTGCGGCACATCGAGGAGAACGTCAAGG  554

Query  1189  GCGCCGTCAAGAGCTGCGTCATGACGGTGGCCAAGAAGACACTCAGCACCAACTCCACCG  1248
             ||||||||||||||||||||||||| ||||||| |||||| || ||||| | | ||||||
Sbjct  553   GCGCCGTCAAGAGCTGCGTCATGACAGTGGCCAGGAAGACTCTGAGCACGAGCGCCACCG  494

Query  1249  GCGATCTCCACGTCGCTCGCTTCTGCGAGAAGGACCTGCTCAAGGAGATCGACCGCGAGG  1308
             |||  ||||||| ||||||||||||||||||||| |||||| |||||||||||||||| |
Sbjct  493   GCGGCCTCCACGCCGCTCGCTTCTGCGAGAAGGATCTGCTCCAGGAGATCGACCGCGAAG  434
```

```
Query  1309  CGGTGTTCGCGTACGCGGACGACCCGTGCAGCGCCAACTACCCGCTGATGAAGAAGCTGC  1368
             | |||||||||||||| ||||||||||||||||||||||||||||||||||||||||| |
Sbjct  433   CCGTGTTCGCGTACGCCGACGACCCGTGCAGCGCCAACTACCCGCTGATGAAGAAGCTCC  374

Query  1369  GCAACGTGCTCGTGGAGCGCGCCCTCGCCAACGGCGCGGCCGAGTTCAACGCCGACACCT  1428
             ||| ||||||||| |||||||| |||||||||||||| ||||||||| |||| || ||||
Sbjct  373   GCAGCGTGCTCGTCGAGCGCGCGCTCGCCAACGGCGCCGCCGAGTTCGACGCGGAGACCT  314

Query  1429  CCGTGTTCGCCAAGGTCGCGCAGTTCGAGGAGGAGCTGCGCGCGACTCTGCCAGGTGCGA  1488
             ||||| ||||||||||||||| ||||||||||||||| |||||| | | |||||  | ||  
Sbjct  313   CCGTGCTCGCCAAGGTCGCGCGGTTCGAGGAGGAGCTCCGCGCGGCGCTGCCCCGGGCCG  254

Query  1489  TCGAGGCCGCACGTGCGGCTGTGGAGAACGGCACGGCGGCGATTCCCAGCAGAATCACCG  1548
             |||||||||| || || || ||||||| |||||||||||||   |||| ||| ||| |||
Sbjct  253   TCGAGGCCGCGCGCGCCGCCGTGGAGAGCGGCACGGCGGCGGCGCCCAACAGGATCGCCG  194

Query  1549  AGTGCAGGTCGTATCCGCTCTACCGGTTCGTGCGCGAGGAGCTCGGAACAAAGTACCTGA  1608
             ||||| | ||||| |||||||||||||||||||||||||||||||| ||    ||||| |
Sbjct  193   AGTGCCGCTCGTACCCGCTCTACCGGTTCGTGCGCGAGGAGCTCGGCACCGCCTACCTCA  134

Query  1609  CCGGCGAGAAGACGCGGTCCCCCGGCGAGGAGCTGAACAAGGTTCTTGTGGCCATCAACG  1668
             ||||||||||||||||||| |||||||||||||| |||||||| || || || |||||| 
Sbjct  133   CCGGCGAGAAGACGCGGTCGCCCGGCGAGGAGCTCAACAAGGTGCTCGTCGCGATCAACC  74

Query  1669  AAGGCAAGCACATCGACCCGTTGCTCGAGTGCCTCAAGGAGTGGAACGGCGAGCCCCTGC  1728
             | |||||||||||||||||| |||||||||||||||||||||||||||||| ||| ||||
Sbjct  73    AGGGCAAGCACATCGACCCGCTGCTCGAGTGCCTCAAGGAGTGGAACGGCGCGCCACTGC  14

Query  1729  CAATCTGCTGA  1739
             |||||||||||
Sbjct  13    CAATCTGCTGA  3
```

Table of SNPs and InDels in Os02t0627100-01

		cds	5' UTR	3'UTR	Introns
No. of variants:	218	218	0	0	0
No. of SNPs:	205	205	0	0	0
No.of InDels:	13	13	0	0	0

S.No: 11

B8: Os08t0468100-03

Query: KMR3: scaffold32698_size2633

Subject: IL50-13: scaffold5531_size12871

Score	Expect	Identities	Gaps	Strand
2891 bits(1565)	0.0	1582/1589(99%)	6/1589(0%)	Plus/Plus

```
Query  1    TCTCATCTTGCATACGCATGCATGCAGCATTTCGTTTCTAATTGGCACGACACGTATATA  60
            ||||||||||||||||||||||||||||||||||||||||||||||||||||||||||||
Sbjct  125  TCTCATCTTGCATACGCATGCATGCAGCATTTCGTTTCTAATTGGCACGACACGTATATA  184

Query  61   CATCGTCGTACGTATACCAAAATAATTAATGCGGTGCGTATAATTTAGTAGTAGGAGTTT  120
            ||||||||||||||||||||||||||||||||||||||||||||||||||||||||||||
Sbjct  185  CATCGTCGTACGTATACCAAAATAATTAATGCGGTGCGTATAATTTAGTAGTAGGAGTTT  244

Query  121  AGAACACGATGAAAGAATTGGCCATGTCGTACTTCATCTTCTCCAGGTTCGGCGAGACGG  180
            ||||||||||||||||||||||||||||||||||||||||||||||||||||||||||||
Sbjct  245  AGAACACGATGAAAGAATTGGCCATGTCGTACTTCATCTTCTCCAGGTTCGGCGAGACGG  304

Query  181  CGAACTTGATCATCGGCGGCGGCCCGCAGGCGAGCGCGAGCGTGTCGTCGCCGCCCTCCG  240
            ||||||||||||||||||||||||||||||||||||||||||||||||||||||||||||
Sbjct  305  CGAACTTGATCATCGGCGGCGGCCCGCAGGCGAGCGCGAGCGTGTCGTCGCCGCCCTCCG  364

Query  241  GCACGTGCTCCCGCAGCACCTCCTCCGTGACGAACCCGACGCCGTACTTCCACCCTTCCT  300
            ||||||||||||||||||||||||||||||||||||||||||||||||||||||||||||
Sbjct  365  GCACGTGCTCCCGCAGCACCTCCTCCGTGACGAACCCGACGCCGTACTTCCACCCTTCCT  424

Query  301  CCGGCCGCTTCACCTGGTCGATGACGTACCACACCTTGAGCCTGTCCGGGTACTCCGCCG  360
            ||||||||||||||||||||||||||||||||||||||||||||||||||||||||||||
Sbjct  425  CCGGCCGCTTCACCTGGTCGATGACGTACCACACCTTGAGCCTGTCCGGGTACTCCGCCG  484

Query  361  CCCACCGGTCGAGCTCGTCGCGGAGGAGGATGTCGTCCTCCGTCCGGTTCGCGTACACCA  420
            ||||||||||||||||||||||||||||||||||||||||||||||||||||||||||||
Sbjct  485  CCCACCGGTCGAGCTCGTCGCGGAGGAGGATGTCGTCCTCCGTCCGGTTCGCGTACACCA  544

Query  421  GGTGCATCTCCGTCGTGTCCTCCGGCTGGTCGCGCAGCACCGACTGGATGACCTGGTACA  480
            ||||||||||||||||||||||||||||||||||||||||||||||||||||||||||||
Sbjct  545  GGTGCATCTCCGTCGTGTCCTCCGGCTGGTCGCGCAGCACCGACTGGATGACCTGGTACA  604

Query  481  TGGGCGTGATCCCGCTCCCGCCGGCGATCATCGCCAGCCGCCGCGCGTTCCGCGGCTTGC  540
            ||||||||||||||||||||||||||||||||||||||||||||||||||||||||||||
Sbjct  605  TGGGCGTGATCCCGCTCCCGCCGGCGATCATCGCCAGCCGCCGCGCGTTCCGCGGCTTGC  664

Query  541  CGTTGATGACGAACTCGCCGCGGCCGGTGTACTCGACGTGGCCGAGTGGCCCCTTGACGT  600
            ||||||||||||||||||||||||||||||||||||||||||||||||||||||||||||
Sbjct  665  CGTTGATGACGAACTCGCCGCGGCCGGTGTACTCGACGTGGCCGAGTGGCCCCTTGACGT  724
```

```
Query  601   CGATGTAGGCGCCCACGGGGAGCGAGTCCAGGTACTGCGTCATGAGCCCGCCATCGGGGA  660
             ||||||||||||||||||||||||||||||||||||||||||||||||||||||||||||
Sbjct  725   CGATGTAGGCGCCCACGGGGAGCGAGTCCAGGTACTGCGTCATGAGCCCGCCATCGGGGA  784

Query  661   ACTTGGGGTGCTCGTTCTTGAAGTACACCTTGATGAGGAGGTCGAAGTGGCCGACCTCGT  720
             ||||||||||||||||||||||||||||||||||||||||||||||||||||||||||||
Sbjct  785   ACTTGGGGTGCTCGTTCTTGAAGTACACCTTGATGAGGAGGTCGAAGTGGCCGACCTCGT  844

Query  721   CGACCATGCTCGTCGGCGTGTACGCCCGCATGCACAGCTTCCCTTCGATGCTGGCGCACA  780
             ||||||||||||||||||||||||||||||||||||||||||||||||||||||||||||
Sbjct  845   CGACCATGCTCGTCGGCGTGTACGCCCGCATGCACAGCTTCCCTTCGATGCTGGCGCACA  904

Query  781   CGAAGATGTGCTTGCCGACGGGGAGGCCGAGCACCTGGTCGGAGGACGGCAGCGCGAAGC  840
             ||||||||||||||||||||||||||||||||||||||||||||||||||||||||||||
Sbjct  905   CGAAGATGTGCTTGCCGACGGGGAGGCCGAGCACCTGGTCGGAGGACGGCAGCGCGAAGC  964

Query  841   GGAAGAGGCGGACGTCGCGGGAGAGCTCCTTCTTGTCGACGAGTTGGCAGGGGACCTTGT  900
             ||||||||||||||||||||||||||||||||||||||||||||||||||||||||||||
Sbjct  965   GGAAGAGGCGGACGTCGCGGGAGAGCTCCTTCTTGTCGACGAGTTGGCAGGGGACCTTGT  1024

Query  901   CGCGCGGGCTGGAGAGCGCGACGGGCGCCGGCGCCTTGATGGCCTCGCGGATGGGGGCGA  960
             ||||||||||||||||||||||||||||||||||||||||||||||||||||||||||||
Sbjct  1025  CGCGCGGGCTGGAGAGCGCGACGGGCGCCGGCGCCTTGATGGCCTCGCGGATGGGGGCGA  1084

Query  961   GCTGGGAGAGGTTGGACGCGCCGTGGACGGAGTTGTCGGAGCTGTACCCGGCGCCGGTGG  1020
             ||||||||||||||||||||||||||||||||||||||||||||||||||||||||||||
Sbjct  1085  GCTGGGAGAGGTTGGACGCGCCGTGGACGGAGTTGTCGGAGCTGTACCCGGCGCCGGTGG  1144

Query  1021  TGATGAGCTCGCCGATGCGGTAGGTGTCGAGGAGCGCCTTGGCCTTGTCGGAGTGGATGG  1080
             ||||||||||||||||||||||||||||||||||||||||||||||||||||||||||||
Sbjct  1145  TGATGAGCTCGCCGATGCGGTAGGTGTCGAGGAGCGCCTTGGCCTTGTCGGAGTGGATGG  1204

Query  1081  CGTCGAACTCCTCGGTGCAGTCGGTGCCGGCGTTGATGAGGATGCTGTCGGCGCCGCCGG  1140
             ||||||||||||||||||||||||||||||||||||||||||||||||||||||||||||
Sbjct  1205  CGTCGAACTCCTCGGTGCAGTCGGTGCCGGCGTTGATGAGGATGCTGTCGGCGCCGCCGG  1264

Query  1141  GGTGGTCCTTGAGGAAGGCCGTGCAGTCGTAGACGTGACCGTGGACGACGATCCACGCCG  1200
             ||||||||||||||||||||||||||||||||||||||||||||||||||||||||||||
Sbjct  1265  GGTGGTCCTTGAGGAAGGCCGTGCAGTCGTAGACGTGACCGTGGACGACGATCCACGCCG  1324

Query  1201  AGTCCTGCGACGAGTGCTTGCGCACCTCGGACATGGTAAACTGCTTGCCGTCGGTGGTGT  1260
             ||||||||||||||||||||||||||||||||||||||||||||||||||||||||||||
Sbjct  1325  AGTCCTGCGACGAGTGCTTGCGCACCTCGGACATGGTAAACTGCTTGCCGTCGGTGGTGT  1384

Query  1261  TCATGAACGGCGTCGACGTGCTCCGCTTCAGCCCCGGTGCGGCCGCCTCCGCCGTCTCCA  1320
             ||||||||||||||||||||||||||||||||||||||||||||||||||||||||||||
Sbjct  1385  TCATGAACGGCGTCGACGTGCTCCGCTTCAGCCCCGGTGCGGCCGCCTCCGCCGTCTCCA  1444

Query  1321  GGTGCTTCTGCCTCGCCATCCACCCGCCGGTCTGGTTCCCGGGCTGCGTCGGGTGCTCGA  1380
             ||||||||||||||||||||||||||||||||||||||||||||||||||||||||||||
Sbjct  1445  GGTGCTTCTGCCTCGCCATCCACCCGCCGGTCTGGTTCCCGGGCTGCGTCGGGTGCTCGA  1504
```

```
Query  1381  ACACCAGCCCGATCTCACCCTTGTGCGGCCGGCACACGTTCACCTTCACCTTGAACCAGC  1440
             ||||||||||||||||||||||||||||||||||||||||||||||||||||||||||||
Sbjct  1505  ACACCAGCCCGATCTCACCCTTGTGCGGCCGGCACACGTTCACCTTCACCTTGAACCAGC  1564

Query  1441  AGTTGTTCATCATCCCCTGCAAAAACCCAACCGATCGAATGAATTTGCCATCGTCGTCTA  1500
             ||||||||||||||||||||||||||||||||||||||||||||||||||||||||||||
Sbjct  1565  AGTTGTTCATCATCCCCTGCAAAAACCCAACCGATCGAATGAATTTGCCATCGTCGTCTA  1624

Query  1501  CATCGCCGTCGCCGTCGCCATGGCCGGAGACGAAATGAAGAACCTACCATGAGATTCCAG  1560
             ||||||||||||| |     ||||||||||||| ||||||||||||||||||||||||||
Sbjct  1625  CATCGCCGTCGCC-T-----TGGCCGGAGACGGAATGAAGAACCTACCATGAGATTCCAG  1678

Query  1561  ATGAGCTTCTCGGGCTGGGTGTTGTGCGA  1589
             |||||||||||||||||||||||||||||
Sbjct  1679  ATGAGCTTCTCGGGCTGGGTGTTGTGCGA  1707
```

Table of SNPs and InDels in Os08t0468100-03

	cds	5' UTR	3'UTR	Introns
No. of variants:7	0	0	0	7
No. of SNPs: 1	0	0	0	1
No.of InDels: 6	0	0	0	6

S.No: 12

B21: Os08t0487301-00

Query: KMR3: scaffold243_size48649

Subject: IL50-13: scaffold6702_size11543

Score	Expect	Identities	Gaps	Strand
492 bits(266)	7e-144	289/300(96%)	2/300(0%)	Plus/Minus

```
Query  1    TCTCCCACTGCCAGTAGCCGGTCCGGGAGAGGCGGCAGAGGAAGGGGAGATCGAAGATGA  60
            ||||||||||||||||||||||||||||||||||||||||||||||||||||||||||||
Sbjct  396  TCTCCCACTGCCAGTAGCCGGTCCGGGAGAGGCGGCAGAGGAAGGGGAGATCGAAGATGA  337

Query  61   CGGCGCCGCCGATGTGGCTGCCGTTTGGTGGGGCGGAGCGGCGGGAGACCGCGGCGGCGG  120
            |||||||||||||||||||||||||||| |||||||||| ||||||||||||||| || ||
Sbjct  336  CGGCGCCGCCGATGTGGCTGCCGTTTGGCGGGGCGGAGCTGCGGGAGACCGCGGTGGTGG  277

Query  121  AGCTAGAGCCACACGTGCGACGGCTCCCTGGTCAACTTGCGCAGCCGCGAACAAGTAGAG  180
            |||| |||||||||| |||||||||||||||| ||||||||||||||||||||||| ||||
Sbjct  276  AGCTGGAGCCACACGCGCGACGGCTCCCTGGCCAACTTGCGCAGCCGCGAACAAGGAGAG  217

Query  181  CCACGCCTTCACCGCCGCCTCGCGATGACGATGGCGATGAGTTCGATCTCCGGCCGCCGC  240
            ||||||||||||||||||||||||||||||||||||||||||||||||||||||||||||
Sbjct  216  CCACGCCTTCACCGCCGCCTCGCGATGACGATGGCGATGAGTTCGATCTCCGGCCGCCGC  157

Query  241  CCAGCCGCGTGCATGAGCTGGAGGAGGTGCCCGGCGAGTTTC-GGCAGTGAGTTTCGTCT  299
            || ||||||||||||||||||||||||||| |||||||||| ||||||||||||||||
Sbjct  156  CCCGCCGCGTGCATGAGCTGGAGGAGGTGCC-GGCGAGTTTCGGGCAGTGAGTTTCGTCT  98
```

Table of SNPs and InDels in Os08t0487301-00

	cds	5' UTR	3'UTR	Introns
No. of variants:11	7	0	0	4
No. of SNPs: 9	5	0	0	4
No.of InDels: 2	2	0	0	0

S.No: 13

B23: Os08t0490100-01

Query: KMR3: scaffold8993_size10535

Subject: IL50-13: scaffold3687_size21453

Score	Expect	Identities	Gaps	Strand
154 bits(83)	3e-42	132/155(85%)	6/155(3%)	Plus/Plus

```
Query  345   TGCCCGCGGTGCGAGTCGCGGGACACCAAATTCTGCTACTACAACAACTACAACACCTCC   404
             |||||||||||| | |||  | ||||||| |||||||||||||||||||||  |||| |
Sbjct  1     TGCCCGCGGTGCAACTCGACGAACACCAAGTTCTGCTACTACAACAACTACA--GCCT-C   57

Query  405   CAGC-CCCG-GC-ACTTCTGCAAGTGTTGCCGCCGCTACTGGACCAAGGGTGGCACGCTC   461
             |||| |||| || |||||||||||   ||||| ||||||||||  |||| ||| |||||
Sbjct  58    CAGCAGCCGCGCTACTTCTGCAAGACGTGCCGGCGCTACTGGACGGAGGGCGGCTCGCTC   117

Query  462   CGCAACGTCCCCGTCGGCGGCGGCACGCGCAAGAA   496
             |||||||||||||||||||||||| ||||||||||
Sbjct  118   CGCAACGTCCCCGTCGGCGGCGGCTCGCGCAAGAA   152
```

Table of SNPs and InDels in Os08t0490100-01

	cds	5' UTR	3'UTR	Introns
No. of variants:23	23	0	0	0
No. of SNPs: 17	17	0	0	0
No.of InDels: 6	6	0	0	0

S.No: 14

C1: Os01t0314800-01

Query: KMR3: scaffold2182_size25019

Subject: IL50-13: scaffold17822_size5094

```
Score             Expect     Identities      Gaps          Strand
800 bits(433)     0.0        435/436(99%)    0/436(0%)     Plus/Minus

Query  296  GTCCACCTCCTTTGCGCCGCCGGCCGGCCGGTAGTACCCGGTCACCGGGTCCGGCACCCA  355
            |||||||||||||||||||||||||||||||||||||||||| |||||||||||||||||
Sbjct  436  GTCCACCTCCTTTGCGCCGCCGGCCGGCCGGTAGTACCCGGTGACCGGGTCCGGCACCCA  377

Query  356  CGCCGTCTTCTCCTCGGCGGCCTTCGCCATCGTGCGCTTGGCCACCGCCGCCGCCACCTT  415
            ||||||||||||||||||||||||||||||||||||||||||||||||||||||||||||
Sbjct  376  CGCCGTCTTCTCCTCGGCGGCCTTCGCCATCGTGCGCTTGGCCACCGCCGCCGCCACCTT  317

Query  416  CTTCTCGTCCGCCCTCCGGCCGGCTCCCTTCGCCACGGCCGCCGCCACCGAGTAACCCCT  475
            ||||||||||||||||||||||||||||||||||||||||||||||||||||||||||||
Sbjct  316  CTTCTCGTCCGCCCTCCGGCCGGCTCCCTTCGCCACGGCCGCCGCCACCGAGTAACCCCT  257

Query  476  CCTGCAAATCATCATCACACATCACCCACGGATTAATTAATTGGCCATCAATACACAAAA  535
            ||||||||||||||||||||||||||||||||||||||||||||||||||||||||||||
Sbjct  256  CCTGCAAATCATCATCACACATCACCCACGGATTAATTAATTGGCCATCAATACACAAAA  197

Query  536  CAACACACTAACACAGCACTTGTAAAGAAAACAAACGATTTCATGGACAACTTGAATGCA  595
            ||||||||||||||||||||||||||||||||||||||||||||||||||||||||||||
Sbjct  196  CAACACACTAACACAGCACTTGTAAAGAAAACAAACGATTTCATGGACAACTTGAATGCA  137

Query  596  TGGAGAGCTAACCTTTGTGCCAGAAGGCTGAGACAGCTCGATGCAACCCTCTCCATGGAT  655
            ||||||||||||||||||||||||||||||||||||||||||||||||||||||||||||
Sbjct  136  TGGAGAGCTAACCTTTGTGCCAGAAGGCTGAGACAGCTCGATGCAACCCTCTCCATGGAT  77

Query  656  GATCAACTTTCTTTCTCTCTCTAAGTTTGAACTATATGTAAAAATGAGCTGGTAAATCAG  715
            ||||||||||||||||||||||||||||||||||||||||||||||||||||||||||||
Sbjct  76   GATCAACTTTCTTTCTCTCTCTAAGTTTGAACTATATGTAAAAATGAGCTGGTAAATCAG  17

Query  716  GAATCTTGTTTGAGTT  731
            ||||||||||||||||
Sbjct  16   GAATCTTGTTTGAGTT  1
```

Table of SNPs and InDels in Os01t0314800-01

	cds	5' UTR	3'UTR	Introns
No. of variants:1	1	0	0	0
No. of SNPs: 1	1	0	0	0
No.of InDels: 0	0	0	0	0

S.No: 15

C4: Os02t0831500-01

Query: KMR3: scaffold8577_size10987

Subject: IL50-13: scaffold5992_size12325

Score	Expect	Identities	Gaps	Strand
5330 bits(2886)	0.0	2889/2890(99%)	1/2890(0%)	Plus/Minus

```
Query  1     AGCT-ATTTAATAAATTTTAAGTAAAATCATACTAAAAAATATATGATTTTGTTTGGATG  59
             |||| |||||||||||||||||||||||||||||||||||||||||||||||||||||||
Sbjct  2890  AGCTAATTTAATAAATTTTAAGTAAAATCATACTAAAAAATATATGATTTTGTTTGGATG  2831

Query  60    CTAGCCACGCAATTGCGCGGGCCACCCAGCTAGTTATCATATATGATACTACATAATCAC  119
             ||||||||||||||||||||||||||||||||||||||||||||||||||||||||||||
Sbjct  2830  CTAGCCACGCAATTGCGCGGGCCACCCAGCTAGTTATCATATATGATACTACATAATCAC  2771

Query  120   TTATTGAGAATTCAGATAGATAGTGACACATGAATAAATGCACATATTGCAGCTAACAAG  179
             ||||||||||||||||||||||||||||||||||||||||||||||||||||||||||||
Sbjct  2770  TTATTGAGAATTCAGATAGATAGTGACACATGAATAAATGCACATATTGCAGCTAACAAG  2711

Query  180   GCTCATACCAGAAGCAAAGGGCACCAAATGCAATGTAGAGTTGGAACCGATTGAAAACAC  239
             ||||||||||||||||||||||||||||||||||||||||||||||||||||||||||||
Sbjct  2710  GCTCATACCAGAAGCAAAGGGCACCAAATGCAATGTAGAGTTGGAACCGATTGAAAACAC  2651

Query  240   AAAGCACTCCAACATCCTCCGTGTGCCATTCAAGACTGAGGATGGCAAAGTTCTTCCACA  299
             ||||||||||||||||||||||||||||||||||||||||||||||||||||||||||||
Sbjct  2650  AAAGCACTCCAACATCCTCCGTGTGCCATTCAAGACTGAGGATGGCAAAGTTCTTCCACA  2591

Query  300   GTGGGTCTCCCGGTTTGACATCTATCCTTACCTGGAGAGATATGCCCAGGTTTGCCAGCC  359
             ||||||||||||||||||||||||||||||||||||||||||||||||||||||||||||
Sbjct  2590  GTGGGTCTCCCGGTTTGACATCTATCCTTACCTGGAGAGATATGCCCAGGTTTGCCAGCC  2531

Query  360   AGTTAGATGCCGATTTCATTTCCTACTGCACATATTTTAATTTTACCTGCAGCATTCATT  419
             ||||||||||||||||||||||||||||||||||||||||||||||||||||||||||||
Sbjct  2530  AGTTAGATGCCGATTTCATTTCCTACTGCACATATTTTAATTTTACCTGCAGCATTCATT  2471

Query  420   GACAATAAACTTCCTTGAATTATTGTACATGTCGATATCAGGATTCTTCAGTCAAGATTC  479
             ||||||||||||||||||||||||||||||||||||||||||||||||||||||||||||
Sbjct  2470  GACAATAAACTTCCTTGAATTATTGTACATGTCGATATCAGGATTCTTCAGTCAAGATTC  2411

Query  480   TCGAAATATTGGAGGGGAAACCAGACTTGGTCATTGGCAATTACACCGACGGTAATCTAG  539
             ||||||||||||||||||||||||||||||||||||||||||||||||||||||||||||
Sbjct  2410  TCGAAATATTGGAGGGGAAACCAGACTTGGTCATTGGCAATTACACCGACGGTAATCTAG  2351

Query  540   TAGCATCCCTCCTGACAAGCAAACTAGGAGTCACTCAGGTGAAAACGGATCACTACAGCA  599
             ||||||||||||||||||||||||||||||||||||||||||||||||||||||||||||
Sbjct  2350  TAGCATCCCTCCTGACAAGCAAACTAGGAGTCACTCAGGTGAAAACGGATCACTACAGCA  2291
```

```
Query  600   ATAAGATCATGCTGGTTTTAAAATAATGGCCAACTTTTGCATATTCATGAGATTATAATG  659
             ||||||||||||||||||||||||||||||||||||||||||||||||||||||||||||
Sbjct  2290  ATAAGATCATGCTGGTTTTAAAATAATGGCCAACTTTTGCATATTCATGAGATTATAATG  2231

Query  660   ACTAAATATGCCAACACATCGCTAAAAGAAACTTACCATCACCAATCAACAGGGAACAAT  719
             ||||||||||||||||||||||||||||||||||||||||||||||||||||||||||||
Sbjct  2230  ACTAAATATGCCAACACATCGCTAAAAGAAACTTACCATCACCAATCAACAGGGAACAAT  2171

Query  720   AGCGCATGCTCTAGAGAAGACGAAGTACGAGGATTCAGACATCAAGTGGAGAGAATTGGA  779
             ||||||||||||||||||||||||||||||||||||||||||||||||||||||||||||
Sbjct  2170  AGCGCATGCTCTAGAGAAGACGAAGTACGAGGATTCAGACATCAAGTGGAGAGAATTGGA  2111

Query  780   CCACAAGTACCATTTCTCATGCCAGTTTACTGCTGACATGATTGCCATGAACACTAGTGA  839
             ||||||||||||||||||||||||||||||||||||||||||||||||||||||||||||
Sbjct  2110  CCACAAGTACCATTTCTCATGCCAGTTTACTGCTGACATGATTGCCATGAACACTAGTGA  2051

Query  840   CTTCATCATCGCTAGTACCTACCAAGAAATAGCTGGAAGGTCAGTTACTCCACAAACCAA  899
             ||||||||||||||||||||||||||||||||||||||||||||||||||||||||||||
Sbjct  2050  CTTCATCATCGCTAGTACCTACCAAGAAATAGCTGGAAGGTCAGTTACTCCACAAACCAA  1991

Query  900   AATCCTTTTACCTTGTGGCTTAGAACTACCACTATGCAGCTGATAGCCTGACATTGGAGC  959
             ||||||||||||||||||||||||||||||||||||||||||||||||||||||||||||
Sbjct  1990  AATCCTTTTACCTTGTGGCTTAGAACTACCACTATGCAGCTGATAGCCTGACATTGGAGC  1931

Query  960   GTCAAAAATATAAAATGCAGCAAAGAGAAGCCTGGCCAGTATGAGAGTCACTATGCATTC  1019
             ||||||||||||||||||||||||||||||||||||||||||||||||||||||||||||
Sbjct  1930  GTCAAAAATATAAAATGCAGCAAAGAGAAGCCTGGCCAGTATGAGAGTCACTATGCATTC  1871

Query  1020  ACTATGCCAGGGCTCTGCCGCTATGCCACAGGCATCAACGTATTTGATCCCAAGTTCAAC  1079
             ||||||||||||||||||||||||||||||||||||||||||||||||||||||||||||
Sbjct  1870  ACTATGCCAGGGCTCTGCCGCTATGCCACAGGCATCAACGTATTTGATCCCAAGTTCAAC  1811

Query  1080  ATTGCTGCACCAGGTGCAGATCAATCTGTTTACTTTCCCTTTACACAAAAGCAGAAGCGC  1139
             ||||||||||||||||||||||||||||||||||||||||||||||||||||||||||||
Sbjct  1810  ATTGCTGCACCAGGTGCAGATCAATCTGTTTACTTTCCCTTTACACAAAAGCAGAAGCGC  1751

Query  1140  CTGACTGACTTACACCCCCAAATTGAGGAGTTGCTCTATAGCAAAGAGGACAATAATGAG  1199
             ||||||||||||||||||||||||||||||||||||||||||||||||||||||||||||
Sbjct  1750  CTGACTGACTTACACCCCCAAATTGAGGAGTTGCTCTATAGCAAAGAGGACAATAATGAG  1691

Query  1200  CATATGTAAGTACATCCCTTGGGAAGCATATCCAGTATGCAGGATCttttttttttACAG  1259
             ||||||||||||||||||||||||||||||||||||||||||||||||||||||||||||
Sbjct  1690  CATATGTAAGTACATCCCTTGGGAAGCATATCCAGTATGCAGGATCTTTTTTTTTTACAG  1631

Query  1260  CATTGCTTTATTAACAACTCGCATCACATGTGGTATGATTAAATCAGAGGGCACCTAGCA  1319
             ||||||||||||||||||||||||||||||||||||||||||||||||||||||||||||
Sbjct  1630  CATTGCTTTATTAACAACTCGCATCACATGTGGTATGATTAAATCAGAGGGCACCTAGCA  1571

Query  1320  GACAGGAGTAAGCCAATCATCTTTTCAATGGCAAGACTTGACAAGATAAAGAACATCACA  1379
             ||||||||||||||||||||||||||||||||||||||||||||||||||||||||||||
Sbjct  1570  GACAGGAGTAAGCCAATCATCTTTTCAATGGCAAGACTTGACAAGATAAAGAACATCACA  1511
```

```
Query  1380  GGGCTGGTCGAATGGTATGGTCAGAACAAGAGGCTCAGGGACCTTGTGAACCTTGTCATT  1439
             ||||||||||||||||||||||||||||||||||||||||||||||||||||||||||||
Sbjct  1510  GGGCTGGTCGAATGGTATGGTCAGAACAAGAGGCTCAGGGACCTTGTGAACCTTGTCATT  1451

Query  1440  GTTGGGGGCCTCCTGGACCCTTCACAGTCAAAGGACCGTGAGGAGATAGAGGAGATAAAC  1499
             ||||||||||||||||||||||||||||||||||||||||||||||||||||||||||||
Sbjct  1450  GTTGGGGGCCTCCTGGACCCTTCACAGTCAAAGGACCGTGAGGAGATAGAGGAGATAAAC  1391

Query  1500  AAGATGCACAGCCTGATAAACAAGTACCAGCTCGTGGGACAGATCCGTTGGATAAAAGGA  1559
             ||||||||||||||||||||||||||||||||||||||||||||||||||||||||||||
Sbjct  1390  AAGATGCACAGCCTGATAAACAAGTACCAGCTCGTGGGACAGATCCGTTGGATAAAAGGA  1331

Query  1560  CAGACTGACCGAGTGCGCAATGGGGAACTTTACCGTTGCATCGCGGACACCAAGGGAGCC  1619
             ||||||||||||||||||||||||||||||||||||||||||||||||||||||||||||
Sbjct  1330  CAGACTGACCGAGTGCGCAATGGGGAACTTTACCGTTGCATCGCGGACACCAAGGGAGCC  1271

Query  1620  TTTGTTCAGGTACACCGTCAACTTAAAAGGATACTACTATTTTCTTGGTTTTAAACATAA  1679
             ||||||||||||||||||||||||||||||||||||||||||||||||||||||||||||
Sbjct  1270  TTTGTTCAGGTACACCGTCAACTTAAAAGGATACTACTATTTTCTTGGTTTTAAACATAA  1211

Query  1680  GACTGCAACACTAACCTTATGACTGGACGCTGTGGCTGTCAGCCTGCACTTTATGAAGCA  1739
             ||||||||||||||||||||||||||||||||||||||||||||||||||||||||||||
Sbjct  1210  GACTGCAACACTAACCTTATGACTGGACGCTGTGGCTGTCAGCCTGCACTTTATGAAGCA  1151

Query  1740  TTCGGACTAACAGTCATCGAGGCAATGAACTGCGGGCTGCCCACCTTTGCAACAAATCAA  1799
             ||||||||||||||||||||||||||||||||||||||||||||||||||||||||||||
Sbjct  1150  TTCGGACTAACAGTCATCGAGGCAATGAACTGCGGGCTGCCCACCTTTGCAACAAATCAA  1091

Query  1800  GGAGGACCTGCAGAGATCATTGTCGATGAGGTTTCGGGTTTCCATATCAACCCGCTCAAT  1859
             ||||||||||||||||||||||||||||||||||||||||||||||||||||||||||||
Sbjct  1090  GGAGGACCTGCAGAGATCATTGTCGATGAGGTTTCGGGTTTCCATATCAACCCGCTCAAT  1031

Query  1860  GGCAAGGAGGCAAGCGACAAGATTGCTGACTTCTTTCAGAAATGCAAGGAAGACCTCATC  1919
             ||||||||||||||||||||||||||||||||||||||||||||||||||||||||||||
Sbjct  1030  GGCAAGGAGGCAAGCGACAAGATTGCTGACTTCTTTCAGAAATGCAAGGAAGACCTCATC  971

Query  1920  TACTGGAGCAAGATGTCCACCGCTGGACTCCAACGCATCTATGAGTGGTAACAATAGCCA  1979
             ||||||||||||||||||||||||||||||||||||||||||||||||||||||||||||
Sbjct  970   TACTGGAGCAAGATGTCCACCGCTGGACTCCAACGCATCTATGAGTGGTAACAATAGCCA  911

Query  1980  TTCCACCTATCACTCTCTTCTCAGCTGAACCTAAATATATATAGCCATCACCAAGAAAAG  2039
             ||||||||||||||||||||||||||||||||||||||||||||||||||||||||||||
Sbjct  910   TTCCACCTATCACTCTCTTCTCAGCTGAACCTAAATATATATAGCCATCACCAAGAAAAG  851

Query  2040  GAACAGTAAAATTTGTGCTGTTTGCATCTATGCCTTGAACACTTTAGATCCAGTCATACC  2099
             ||||||||||||||||||||||||||||||||||||||||||||||||||||||||||||
Sbjct  850   GAACAGTAAAATTTGTGCTGTTTGCATCTATGCCTTGAACACTTTAGATCCAGTCATACC  791

Query  2100  ATGCTAAAATCCTAGGTGGATACCTTATGTTTTTCTTAATAAACTAAGGGCCATGCTTG  2159
             ||||||||||||||||||||||||||||||||||||||||||||||||||||||||||||
Sbjct  790   ATGCTAAAATCCTAGGTGGATACCTTATGTTTTTCTTAATAAACTAAGGGCCATGCTTG  731
```

```
Query  2160  TCTATGGTTGAAGTTACACGTGGCAGATCTATGCAACCAAAGTTCTGAACATGGCATCAA  2219
             ||||||||||||||||||||||||||||||||||||||||||||||||||||||||||||
Sbjct  730   TCTATGGTTGAAGTTACACGTGGCAGATCTATGCAACCAAAGTTCTGAACATGGCATCAA  671

Query  2220  TATATGGCTTCTGGAGGACTCTAGACAAGGAAGAAAGACAAGCTAAACAGCACTACCTAC  2279
             ||||||||||||||||||||||||||||||||||||||||||||||||||||||||||||
Sbjct  670   TATATGGCTTCTGGAGGACTCTAGACAAGGAAGAAAGACAAGCTAAACAGCACTACCTAC  611

Query  2280  ACATGTTCTATAATCTTCAATTTAGGAAGTTGGTGAGTTTAGGACCAGTGCAAGACAAAC  2339
             ||||||||||||||||||||||||||||||||||||||||||||||||||||||||||||
Sbjct  610   ACATGTTCTATAATCTTCAATTTAGGAAGTTGGTGAGTTTAGGACCAGTGCAAGACAAAC  551

Query  2340  AATATGCTGAAAATCTATGAATATATTTTGTTCATTTCTTTCAATTTTTCAGGCAAAGAA  2399
             ||||||||||||||||||||||||||||||||||||||||||||||||||||||||||||
Sbjct  550   AATATGCTGAAAATCTATGAATATATTTTGTTCATTTCTTTCAATTTTTCAGGCAAAGAA  491

Query  2400  TGTGCCAACACTGGGCGAACAACCAGCACAACCTACAGAGAGCGCAGAGCCTAATAGGAT  2459
             ||||||||||||||||||||||||||||||||||||||||||||||||||||||||||||
Sbjct  490   TGTGCCAACACTGGGCGAACAACCAGCACAACCTACAGAGAGCGCAGAGCCTAATAGGAT  431

Query  2460  CATACCAAGACCCAAAGAAAGGCAAGTGTGCCCATTCTTACGAAATTTACTGAAGAAAGA  2519
             ||||||||||||||||||||||||||||||||||||||||||||||||||||||||||||
Sbjct  430   CATACCAAGACCCAAAGAAAGGCAAGTGTGCCCATTCTTACGAAATTTACTGAAGAAAGA  371

Query  2520  GACAGGGAACAACTGACTGAAACATTTGTCGTTTGAGCGCTGCAGAAGGACGCAAATAAG  2579
             ||||||||||||||||||||||||||||||||||||||||||||||||||||||||||||
Sbjct  370   GACAGGGAACAACTGACTGAAACATTTGTCGTTTGAGCGCTGCAGAAGGACGCAAATAAG  311

Query  2580  GATCCAGAGGTGAGAATTCTCAGCACTGATGAAGCTGCAAAAACAGCAAACAGAGAGCAT  2639
             ||||||||||||||||||||||||||||||||||||||||||||||||||||||||||||
Sbjct  310   GATCCAGAGGTGAGAATTCTCAGCACTGATGAAGCTGCAAAAACAGCAAACAGAGAGCAT  251

Query  2640  CACTCCAGACTGAGATATTAATCCATTAATATACTGTTGTGTCATGCAGGATTGCAACCA  2699
             ||||||||||||||||||||||||||||||||||||||||||||||||||||||||||||
Sbjct  250   CACTCCAGACTGAGATATTAATCCATTAATATACTGTTGTGTCATGCAGGATTGCAACCA  191

Query  2700  ACTTACTTGGACCACTGCTCCCAGCCTCCAATTTCTCAACAGATGGAGCTTGAAGTAGAG  2759
             ||||||||||||||||||||||||||||||||||||||||||||||||||||||||||||
Sbjct  190   ACTTACTTGGACCACTGCTCCCAGCCTCCAATTTCTCAACAGATGGAGCTTGAAGTAGAG  131

Query  2760  ACCAATCGTCAGAACTTTCTGAAGTTGTTGGAGTGAGAAATGTATTGTATAACATTGATT  2819
             ||||||||||||||||||||||||||||||||||||||||||||||||||||||||||||
Sbjct  130   ACCAATCGTCAGAACTTTCTGAAGTTGTTGGAGTGAGAAATGTATTGTATAACATTGATT  71

Query  2820  GTGAAGATTTAATAAATGATTACTGTGGGAATAATAAAAAGATTAACATACTTGGTAGCA  2879
             ||||||||||||||||||||||||||||||||||||||||||||||||||||||||||||
Sbjct  70    GTGAAGATTTAATAAATGATTACTGTGGGAATAATAAAAAGATTAACATACTTGGTAGCA  11

Query  2880  CTGACTACTT  2889
             ||||||||||
Sbjct  10    CTGACTACTT  1
```

Table of SNPs and InDels in Os02t0831500-01

	cds	5' UTR	3'UTR	Introns
No. of variants:1	0	0	0	1
No. of SNPs: 0	0	0	0	0
No.of InDels: 1	0	0	0	1

S.No: 16

C10: Os06t0229800-01

Query: KMR3: scaffold1456_size29059

Subject: IL50-13: scaffold8945_size9534

Score	Expect	Identities	Gaps	Strand
6684 bits(3619)	0.0	3621/3622(99%)	0/3622(0%)	Plus/Minus

```
Query  870   AACCCGATCAGCGGCACGTCGTCGCGCACCTCCAGCCCCAGCTCGCGCTGCAGCGCCGCC  929
             ||||||||||||||||||||||||||||||||||||||| ||||||||||||||||||||
Sbjct  3623  AACCCGATCAGCGGCACGTCGTCGCGCACCTCCAGCCCCGGCTCGCGCTGCAGCGCCGCC  3564

Query  930   TTGCACCGCGGCTTGCCGGAGTCCAGCGAGGCCACGGTGTAGTTGGCGTAGCCGTCGGAC  989
             ||||||||||||||||||||||||||||||||||||||||||||||||||||||||||||
Sbjct  3563  TTGCACCGCGGCTTGCCGGAGTCCAGCGAGGCCACGGTGTAGTTGGCGTAGCCGTCGGAC  3504

Query  990   TGCAGGTGCACGTCCACCTCCGGGTTCCACTCCCGGTAGTCGATGCCGTTCACGATGCCG  1049
             ||||||||||||||||||||||||||||||||||||||||||||||||||||||||||||
Sbjct  3503  TGCAGGTGCACGTCCACCTCCGGGTTCCACTCCCGGTAGTCGATGCCGTTCACGATGCCG  3444

Query  1050  TTCATCTTCCAGTCGTTCTCCCGTATGATGTCGTGGAGGCCCCAGCCGCCCTCCGTCGTC  1109
             ||||||||||||||||||||||||||||||||||||||||||||||||||||||||||||
Sbjct  3443  TTCATCTTCCAGTCGTTCTCCCGTATGATGTCGTGGAGGCCCCAGCCGCCCTCCGTCGTC  3384

Query  1110  TTCAGCTCCCAGAGGTAGCCGGGGCTCACGGTCACCACCCGGTCCGCCATCTTCAGGCCC  1169
             ||||||||||||||||||||||||||||||||||||||||||||||||||||||||||||
Sbjct  3383  TTCAGCTCCCAGAGGTAGCCGGGGCTCACGGTCACCACCCGGTCCGCCATCTTCAGGCCC  3324

Query  1170  GCGCCGAAGATGTTGGCGTGCTCGCCGCCGACGGGGTCGTACAGCTTGAAGTGATCCAGG  1229
             ||||||||||||||||||||||||||||||||||||||||||||||||||||||||||||
Sbjct  3323  GCGCCGAAGATGTTGGCGTGCTCGCCGCCGACGGGGTCGTACAGCTTGAAGTGATCCAGG  3264

Query  1230  TAGTGCTCCGGCAATTCCATGTAGGGGAATTCATCTACTGGGCCACGGCCCTACAAACAT  1289
             ||||||||||||||||||||||||||||||||||||||||||||||||||||||||||||
Sbjct  3263  TAGTGCTCCGGCAATTCCATGTAGGGGAATTCATCTACTGGGCCACGGCCCTACAAACAT  3204

Query  1290  TACACGAACCATTTAGCATCACaaaaaaaGTACTCCCCGGCACGTTTCATATTGTAAATT  1349
             ||||||||||||||||||||||||||||||||||||||||||||||||||||||||||||
Sbjct  3203  TACACGAACCATTTAGCATCACAAAAAAAGTACTCCCCGGCACGTTTCATATTGTAAATT  3144

Query  1350  GTTTTGATAtttttttAAGTTAAACAGTTTTAAATTTAATTAGTTTATAGAAAAATATAA  1409
             ||||||||||||||||||||||||||||||||||||||||||||||||||||||||||||
Sbjct  3143  GTTTTGATATTTTTTTAAGTTAAACAGTTTTAAATTTAATTAGTTTATAGAAAAATATAA  3084

Query  1410  TAACATTTCCAATACAAAACAAACATATAATCAAATTACATTAAACATTATATTTAATTA  1469
             ||||||||||||||||||||||||||||||||||||||||||||||||||||||||||||
Sbjct  3083  TAACATTTCCAATACAAAACAAACATATAATCAAATTACATTAAACATTATATTTAATTA  3024

Query  1470  AACTAATTTGATGTTATAGATGCTGTTAAATTTTTCTATAAATTTAATCAAACGTAAAGA  1529
             ||||||||||||||||||||||||||||||||||||||||||||||||||||||||||||
Sbjct  3023  AACTAATTTGATGTTATAGATGCTGTTAAATTTTTCTATAAATTTAATCAAACGTAAAGA  2964
```

```
Query  1530  TGTTTGATTGGaaaaaaaaCGACTTATAAATTATAATATGAACGAAGAAAGTGCTGAAC  1589
             ||||||||||||||||||||||||||||||||||||||||||||||||||||||||||||
Sbjct  2963  TGTTTGATTGGAAAAAAAACGACTTATAAATTATAATATGAACGAAGAAAGTGCTGAAC  2904

Query  1590  TAAAAGTAGTAATAGACTGAAGAAGAGTTTATCGTGTCCCTGCTCAATAATTACCATGAC  1649
             ||||||||||||||||||||||||||||||||||||||||||||||||||||||||||||
Sbjct  2903  TAAAAGTAGTAATAGACTGAAGAAGAGTTTATCGTGTCCCTGCTCAATAATTACCATGAC  2844

Query  1650  CCACAATTTGTGCACTCAACTAATTTCTCTAACAAACAGCAGGAGATGCATACAAATAGA  1709
             ||||||||||||||||||||||||||||||||||||||||||||||||||||||||||||
Sbjct  2843  CCACAATTTGTGCACTCAACTAATTTCTCTAACAAACAGCAGGAGATGCATACAAATAGA  2784

Query  1710  ATAAAGATGATTCATTAGGGAGGAGAGAAAAACAAAACCTGGTAAGCGATATTATGTATC  1769
             ||||||||||||||||||||||||||||||||||||||||||||||||||||||||||||
Sbjct  2783  ATAAAGATGATTCATTAGGGAGGAGAGAAAAACAAAACCTGGTAAGCGATATTATGTATC  2724

Query  1770  ACAAGGACAGAGCGAGTGTACTGCATCATGCCATTGTCTCTGTAATATGCCTTCAGATAA  1829
             ||||||||||||||||||||||||||||||||||||||||||||||||||||||||||||
Sbjct  2723  ACAAGGACAGAGCGAGTGTACTGCATCATGCCATTGTCTCTGTAATATGCCTTCAGATAA  2664

Query  1830  ACAGGCAGGAGTGCAGTGTGCCAATCGTTTGCAAGGAACACCAAGTTGCCATCCCCATAG  1889
             ||||||||||||||||||||||||||||||||||||||||||||||||||||||||||||
Sbjct  2663  ACAGGCAGGAGTGCAGTGTGCCAATCGTTTGCAAGGAACACCAAGTTGCCATCCCCATAG  2604

Query  1890  GGCACACCACCGCATGGAACGTGCCAAGGAACCTGCCGATTGTACAGAAACAGAAAATAT  1949
             ||||||||||||||||||||||||||||||||||||||||||||||||||||||||||||
Sbjct  2603  GGCACACCACCGCATGGAACGTGCCAAGGAACCTGCCGATTGTACAGAAACAGAAAATAT  2544

Query  1950  AGCCCTTTGTAGGGAAACATATGACAACATTGGCAAGAGCATTGCATATTACGAACTGTA  2009
             ||||||||||||||||||||||||||||||||||||||||||||||||||||||||||||
Sbjct  2543  AGCCCTTTGTAGGGAAACATATGACAACATTGGCAAGAGCATTGCATATTACGAACTGTA  2484

Query  2010  GCAGGTTGAAATGCTGCTAGAGGTATCGAAGGAGTTCATCCGTGTATTTGGCGATAATGT  2069
             ||||||||||||||||||||||||||||||||||||||||||||||||||||||||||||
Sbjct  2483  GCAGGTTGAAATGCTGCTAGAGGTATCGAAGGAGTTCATCCGTGTATTTGGCGATAATGT  2424

Query  2070  GTAATATATTTAAAATAAAAAAGAATATGAATCTCTGTGAAATCTTGCAGAAACATTTAC  2129
             ||||||||||||||||||||||||||||||||||||||||||||||||||||||||||||
Sbjct  2423  GTAATATATTTAAAATAAAAAAGAATATGAATCTCTGTGAAATCTTGCAGAAACATTTAC  2364

Query  2130  GGGCTGTTTGTTTGAGGGATCAAATTCAAATCATCTACTGCGACCTCATTTCTCCTACTT  2189
             ||||||||||||||||||||||||||||||||||||||||||||||||||||||||||||
Sbjct  2363  GGGCTGTTTGTTTGAGGGATCAAATTCAAATCATCTACTGCGACCTCATTTCTCCTACTT  2304

Query  2190  ATTGTGTGCTTCAGTCCAAAAGTTAATACACTTCTTCTATTTATCAACAGAAAAAATGAT  2249
             ||||||||||||||||||||||||||||||||||||||||||||||||||||||||||||
Sbjct  2303  ATTGTGTGCTTCAGTCCAAAAGTTAATACACTTCTTCTATTTATCAACAGAAAAAATGAT  2244

Query  2250  TTGTGCTTTTGACCATTCCTCAAACCAAACAAATACATCTCATTCCTAAAACCAAATTAA  2309
             ||||||||||||||||||||||||||||||||||||||||||||||||||||||||||||
Sbjct  2243  TTGTGCTTTTGACCATTCCTCAAACCAAACAAATACATCTCATTCCTAAAACCAAATTAA  2184
```

```
Query  2310  TGATTGATAATGGAGTTGGAGAAAATACCTCAACAGCAGCCTTACAAAACAGAATCATGC  2369
             ||||||||||||||||||||||||||||||||||||||||||||||||||||||||||||
Sbjct  2183  TGATTGATAATGGAGTTGGAGAAAATACCTCAACAGCAGCCTTACAAAACAGAATCATGC  2124

Query  2370  GCTTCATGATTTCCTGAATGAAAATGGGAATGGGTAAGCATTTTCAAATCAGTACGCCTA  2429
             ||||||||||||||||||||||||||||||||||||||||||||||||||||||||||||
Sbjct  2123  GCTTCATGATTTCCTGAATGAAAATGGGAATGGGTAAGCATTTTCAAATCAGTACGCCTA  2064

Query  2430  GGAGATGCAACTTAAGTTTTACTAATGCAATGTAATACCAAACAGATAGAAGCTTCACCT  2489
             ||||||||||||||||||||||||||||||||||||||||||||||||||||||||||||
Sbjct  2063  GGAGATGCAACTTAAGTTTTACTAATGCAATGTAATACCAAACAGATAGAAGCTTCACCT  2004

Query  2490  GTCTGTTCCCCCCATAGATGTCATCCTGACGGTGACGGAAGAGAGGAGCGTCAATGAACA  2549
             ||||||||||||||||||||||||||||||||||||||||||||||||||||||||||||
Sbjct  2003  GTCTGTTCCCCCCATAGATGTCATCCTGACGGTGACGGAAGAGAGGAGCGTCAATGAACA  1944

Query  2550  CAAAATCAACTCCATCGATAAATGCATGGAAATATTTCACTTCCAGATCCTGCAGGCCAA  2609
             ||||||||||||||||||||||||||||||||||||||||||||||||||||||||||||
Sbjct  1943  CAAAATCAACTCCATCGATAAATGCATGGAAATATTTCACTTCCAGATCCTGCAGGCCAA  1884

Query  2610  AATAAAAACCAATTACTAAGGAATTTGCGTCTCCTATGCCAGTCTTCTTGGATTGCATTG  2669
             ||||||||||||||||||||||||||||||||||||||||||||||||||||||||||||
Sbjct  1883  AATAAAAACCAATTACTAAGGAATTTGCGTCTCCTATGCCAGTCTTCTTGGATTGCATTG  1824

Query  2670  TTGACACACACCTGTCCAGCAGCCTTGTAGTATTTCCTGATTCCTACATCCTGGGCTTCC  2729
             ||||||||||||||||||||||||||||||||||||||||||||||||||||||||||||
Sbjct  1823  TTGACACACACCTGTCCAGCAGCCTTGTAGTATTTCCTGATTCCTACATCCTGGGCTTCC  1764

Query  2730  GCGTAATCACCGTACCTTGGTACCACAACCTAAGATTGAAAGATGCATTTGCCAGTTATC  2789
             ||||||||||||||||||||||||||||||||||||||||||||||||||||||||||||
Sbjct  1763  GCGTAATCACCGTACCTTGGTACCACAACCTAAGATTGAAAGATGCATTTGCCAGTTATC  1704

Query  2790  AGTTTATATCAGGAAGTCACCGAGAGTCCCCGTTTCCGATTTCTGAATACGTTGTGGTTT  2849
             ||||||||||||||||||||||||||||||||||||||||||||||||||||||||||||
Sbjct  1703  AGTTTATATCAGGAAGTCACCGAGAGTCCCCGTTTCCGATTTCTGAATACGTTGTGGTTT  1644

Query  2850  ATATTATTACCATAACTAAAATTGCTaaaaaaaaaTTGCTACATTTCTCTACTATCTTAA  2909
             ||||||||||||||||||||||||||||||||||||||||||||||||||||||||||||
Sbjct  1643  ATATTATTACCATAACTAAAATTGCTAAAAAAAAATTGCTACATTTCTCTACTATCTTAA  1584

Query  2910  AAGCGAAGTTGTCTTCCTTCCATCCCCTCCTCCCAACGCGTGCAGCGTGTCCATATGCGA  2969
             ||||||||||||||||||||||||||||||||||||||||||||||||||||||||||||
Sbjct  1583  AAGCGAAGTTGTCTTCCTTCCATCCCCTCCTCCCAACGCGTGCAGCGTGTCCATATGCGA  1524

Query  2970  AAAGTAACTACCGTCGCTCCCTAATTATAAGGGCCTAGTTCGTTAACGGTCTCCTAATCT  3029
             ||||||||||||||||||||||||||||||||||||||||||||||||||||||||||||
Sbjct  1523  AAAGTAACTACCGTCGCTCCCTAATTATAAGGGCCTAGTTCGTTAACGGTCTCCTAATCT  1464

Query  3030  ATCCCTAATTGTATGGGCCTGATCCGTTATCATGTACAGGTATGTAACTAGTGTAACTAA  3089
             ||||||||||||||||||||||||||||||||||||||||||||||||||||||||||||
Sbjct  1463  ATCCCTAATTGTATGGGCCTGATCCGTTATCATGTACAGGTATGTAACTAGTGTAACTAA  1404
```

```
Query  3090  GAGAACTACTTTTGGCACTAGTTTCCATGGCCACGTGATGATCTAAGCGGCTACGCCATA  3149
             ||||||||||||||||||||||||||||||||||||||||||||||||||||||||||||
Sbjct  1403  GAGAACTACTTTTGGCACTAGTTTCCATGGCCACGTGATGATCTAAGCGGCTACGCCATA  1344

Query  3150  GTGTATATATTTTCAACAGCATGTTAGAAATGAACATTTCTGTAACAATAGTAAGCAATT  3209
             ||||||||||||||||||||||||||||||||||||||||||||||||||||||||||||
Sbjct  1343  GTGTATATATTTTCAACAGCATGTTAGAAATGAACATTTCTGTAACAATAGTAAGCAATT  1284

Query  3210  TAGCTTTCAAGTACAGATTTGCAAATTTATCAATATTACTTTTGTATTTTGAATCTAACA  3269
             ||||||||||||||||||||||||||||||||||||||||||||||||||||||||||||
Sbjct  1283  TAGCTTTCAAGTACAGATTTGCAAATTTATCAATATTACTTTTGTATTTTGAATCTAACA  1224

Query  3270  GCTGGAGTAGCCTTTTTAATTTGCCATCATGATTATGTAAACAAAAGAACATAAGAGAAC  3329
             ||||||||||||||||||||||||||||||||||||||||||||||||||||||||||||
Sbjct  1223  GCTGGAGTAGCCTTTTTAATTTGCCATCATGATTATGTAAACAAAAGAACATAAGAGAAC  1164

Query  3330  TAATCTGCTTTACATTTGATCCAAATTTATATATCTGAAAATATACGTTAATTAAATGAT  3389
             ||||||||||||||||||||||||||||||||||||||||||||||||||||||||||||
Sbjct  1163  TAATCTGCTTTACATTTGATCCAAATTTATATATCTGAAAATATACGTTAATTAAATGAT  1104

Query  3390  CAAATCTGTAAGAATGGAAGAAACATCCAACCATGAAGGAGAGAAAAAAGGAGTAATACC  3449
             ||||||||||||||||||||||||||||||||||||||||||||||||||||||||||||
Sbjct  1103  CAAATCTGTAAGAATGGAAGAAACATCCAACCATGAAGGAGAGAAAAAAGGAGTAATACC  1044

Query  3450  ATAACACGATGTCCTCTCCTCGCCAAAGCTTTGGGTAAAGCACCTGCAACATCTCCAAGC  3509
             ||||||||||||||||||||||||||||||||||||||||||||||||||||||||||||
Sbjct  1043  ATAACACGATGTCCTCTCCTCGCCAAAGCTTTGGGTAAAGCACCTGCAACATCTCCAAGC  984

Query  3510  CCACCTGTGCAAATGCAACACTTGTTCATATCAGTAACTGAAGCAAATTTACAAGATCAG  3569
             ||||||||||||||||||||||||||||||||||||||||||||||||||||||||||||
Sbjct  983   CCACCTGTGCAAATGCAACACTTGTTCATATCAGTAACTGAAGCAAATTTACAAGATCAG  924

Query  3570  AAGAACaaaaaagagaaaaaaGAAGTATATATCGTGTACATGCCTGTTTTGCACCAGGGA  3629
             ||||||||||||||||||||||||||||||||||||||||||||||||||||||||||||
Sbjct  923   AAGAACAAAAAAGAGAAAAAAGAAGTATATATCGTGTACATGCCTGTTTTGCACCAGGGA  864

Query  3630  GAACATTCAGCAGCCACCACGATCACGTTCATGACATTCTCCCCAGCCAAAGGGCCCGAA  3689
             ||||||||||||||||||||||||||||||||||||||||||||||||||||||||||||
Sbjct  863   GAACATTCAGCAGCCACCACGATCACGTTCATGACATTCTCCCCAGCCAAAGGGCCCGAA  804

Query  3690  TCGTCATCCTGGTCGATCTCAGAATCTGACGCATCTGAATCAGCCCAGTCATCATCGTCA  3749
             ||||||||||||||||||||||||||||||||||||||||||||||||||||||||||||
Sbjct  803   TCGTCATCCTGGTCGATCTCAGAATCTGACGCATCTGAATCAGCCCAGTCATCATCGTCA  744

Query  3750  TCGCCATCCTCCGCGGCGTCCGGTTCGTTCAGATCAAAATATTTCTTGAAATCCCACGTC  3809
             ||||||||||||||||||||||||||||||||||||||||||||||||||||||||||||
Sbjct  743   TCGCCATCCTCCGCGGCGTCCGGTTCGTTCAGATCAAAATATTTCTTGAAATCCCACGTC  684

Query  3810  TCCTCTTCTACCGCAGGAATAGGGGAAGATCTAGTCGCCTTTGGCTTCGGAGCACTCTCA  3869
             ||||||||||||||||||||||||||||||||||||||||||||||||||||||||||||
Sbjct  683   TCCTCTTCTACCGCAGGAATAGGGGAAGATCTAGTCGCCTTTGGCTTCGGAGCACTCTCA  624
```

```
Query  3870  ACAACTTTTGCGCTCTTATCCTCTGCAAATTCCGAATTGTCAGGCTTGGGGAGAGGAGCG  3929
             ||||||||||||||||||||||||||||||||||||||||||||||||||||||||||||
Sbjct  623   ACAACTTTTGCGCTCTTATCCTCTGCAAATTCCGAATTGTCAGGCTTGGGGAGAGGAGCG  564

Query  3930  GACGACCTGGGATTTGAACCGGGCGGTGGCGGCGCCGGCGCCGTCGCGTCTGGGATGACG  3989
             ||||||||||||||||||||||||||||||||||||||||||||||||||||||||||||
Sbjct  563   GACGACCTGGGATTTGAACCGGGCGGTGGCGGCGCCGGCGCCGTCGCGTCTGGGATGACG  504

Query  3990  AACTCCGGCTGCGGCTTGTCTACGGATGGAAGGATCACGGGGGAGTCCGGCGCTGGGAGC  4049
             ||||||||||||||||||||||||||||||||||||||||||||||||||||||||||||
Sbjct  503   AACTCCGGCTGCGGCTTGTCTACGGATGGAAGGATCACGGGGGAGTCCGGCGCTGGGAGC  444

Query  4050  TTCGTTATGGTCACCGGCGGCGTGGCGGCGGCAGGCTTGTCGGCGCCGGTAACGACCGAT  4109
             ||||||||||||||||||||||||||||||||||||||||||||||||||||||||||||
Sbjct  443   TTCGTTATGGTCACCGGCGGCGTGGCGGCGGCAGGCTTGTCGGCGCCGGTAACGACCGAT  384

Query  4110  CCGTTCCGTGAGGCAGGTGTGTCGTCGTCGCGGCCGCTGAGAAGCGCTCCGTTCTTACTT  4169
             ||||||||||||||||||||||||||||||||||||||||||||||||||||||||||||
Sbjct  383   CCGTTCCGTGAGGCAGGTGTGTCGTCGTCGCGGCCGCTGAGAAGCGCTCCGTTCTTACTT  324

Query  4170  GAGGCGGCGTCCTGTGTcggcgccggcgccggcgcggccgccccgcgcactctcgccgcg  4229
             ||||||||||||||||||||||||||||||||||||||||||||||||||||||||||||
Sbjct  323   GAGGCGGCGTCCTGTGTCGGCGCCGGCGCCGGCGCGGCCGCCCCGCGCACTCTCGCCGCG  264

Query  4230  tcaccgccggagccgtaccggccgACGGGCGGGACGGTGCCCCTCCGCTTCAAGACCTAC  4289
             ||||||||||||||||||||||||||||||||||||||||||||||||||||||||||||
Sbjct  263   TCACCGCCGGAGCCGTACCGGCCGACGGGCGGGACGGTGCCCCTCCGCTTCAAGACCTAC  204

Query  4290  AAACACAGCGCGCGCACGCGCAAGCACACGCGTCGGTTTCAAACGAAAACATCAAAGCGA  4349
             ||||||||||||||||||||||||||||||||||||||||||||||||||||||||||||
Sbjct  203   AAACACAGCGCGCGCACGCGCAAGCACACGCGTCGGTTTCAAACGAAAACATCAAAGCGA  144

Query  4350  ATCAAAGCAGAGAACACCTCTCGAGCACGACGATCTCGGCGCCCCGATCGACACCACCAA  4409
             ||||||||||||||||||||||||||||||||||||||||||||||||||||||||||||
Sbjct  143   ATCAAAGCAGAGAACACCTCTCGAGCACGACGATCTCGGCGCCCCGATCGACACCACCAA  84

Query  4410  CCCACCTTGCCGACGCCGCCGCGACGCGAGCGGGGCGGCTGCCACGCGCCCGAAGAGAAC  4469
             ||||||||||||||||||||||||||||||||||||||||||||||||||||||||||||
Sbjct  83    CCCACCTTGCCGACGCCGCCGCGACGCGAGCGGGGCGGCTGCCACGCGCCCGAAGAGAAC  24

Query  4470  TCCTCCTCCTCCTCGTCGTCCT  4491
             ||||||||||||||||||||||
Sbjct  23    TCCTCCTCCTCCTCGTCGTCCT  2
```

Table of SNPs and InDels in Os06t0229800-01

	cds	5' UTR	3'UTR	Introns
No. of variants:1	1	0	0	0
No. of SNPs: 1	1	0	0	0
No.of InDels: 0	0	0	0	0

S.No: 17

D6: Os03t0407400-00

Query: KMR3: scaffold14776_size6169

Subject: I150-13: scaffold6119_size12184

Score	Expect	Identities	Gaps	Strand
931 bits(504)	0.0	506/507(99%)	0/507(0%)	Plus/Minus

```
Query  1    GTAAAATAAATCAGCAATCACGTACTCATCATGGCAGCAAAGCCAAATTACAGATAACCG  60
            ||||||||||||||||||||||||||||||||||||||||||||||||||||||||||||
Sbjct  507  GTAAAATAAATCAGCAATCACGTACTCATCATGGCAGCAAAGCCAAATTACAGATAACCG  448

Query  61   CAAGCAGAGCAAGGCAATCAACATATGCATCGATGAGGAACTAATTAACACCAGAACCTA  120
            ||||||||||||||||||||||||||||||||||||||||||||||||||||||||||||
Sbjct  447  CAAGCAGAGCAAGGCAATCAACATATGCATCGATGAGGAACTAATTAACACCAGAACCTA  388

Query  121  GCTAGCTAATTAAGTACGCGCGCAGCTCGATCGATCATCATCGAGAACTAATTAATTAAC  180
            ||||||||||||||||||||||||||||||||||||||||||||||||||||||||||||
Sbjct  387  GCTAGCTAATTAAGTACGCGCGCAGCTCGATCGATCATCATCGAGAACTAATTAATTAAC  328

Query  181  CAATCAGTGCAGCTTCGCTCAATCGATCGATCGATCACAAGCAggggggggCAGCAACGAG  240
            ||||||||||||||||||||||||||||||||||||||||||||||||||||||||||||
Sbjct  327  CAATCAGTGCAGCTTCGCTCAATCGATCGATCGATCACAAGCAGGGGGGGCAGCAACGAG  268

Query  241  GGACGCCGCAGCAGCACCTGCACGCCGGGCACGCGCAGGAGCAGCCGCCGGggcacgggc  300
            ||||||||||||||||||||||||||||||||||||||||||||||||||||||||||||
Sbjct  267  GGACGCCGCAGCAGCACCTGCACGCCGGGCACGCGCAGGAGCAGCCGCCGGGGCACGGGC  208

Query  301  acgggcaggtgcagcggcacgagcatcccgcgcacggcggcgcgcagcacgcgcacggcg  360
            ||||||||||||||||||||||||||||||||||||||||||||||||||||||||||||
Sbjct  207  ACGGGCAGGTGCAGCGGCACGAGCATCCCGCGCACGGCGGCGCGCAGCACGCGCACGGCG  148

Query  361  gggagcagctgcagctcgcgcacgcgcggacgccgacgccgccgcagcagcagcgacggc  420
            ||||||||||||||||||||||||||||||||||||||||||||||||||||||||||||
Sbjct  147  GGGAGCAGCTGCAGCTCGCGCACGCGCGGACGCCGACGCCGCCGCAGCAGCAGCGACGGC  88

Query  421  agcagcagctgcggcggcggcagcgACACGGACTCTTCGTTAACGCCGCCCCACATGAGG  480
            ||||||||||||||||||||||||||||||||||||||||||||||||||||||||||||
Sbjct  87   AGCAGCAGCTGCGGCGGCGGCAGCGACACGGACTCTTCGTTAACGCCGCCCCACATGAGG  28

Query  481  AGGAGGAGGagcagcagcagttgcagt  507
            ||||||||||| |||||||||||||||
Sbjct  27   AGGAGGAGGAGGAGCAGCAGTTGCAGT  1
```

Table of SNPs and InDels in Os03t0407400-00

	cds	5' UTR	3'UTR	Introns
No. of variants:1	1	0	0	0
No. of SNPs: 1	1	0	0	0
No.of InDels: 0	0	0	0	0

S.No: 18

D8: Os03t0117900-01

Query: KMR3: scaffold9265_size10248

Subject: IL50-13: Scaffold21042_size4123

Score	Expect	Identities	Gaps	Strand
819 bits(443)	0.0	445/446(99%)	0/446(0%)	Plus/Plus

```
Query  507   ccggaggccgccgtcgccgcTGGGGCGGGCGATCAGAAGCGCGTCGTGGACGAGGACGAC  566
             |||||||||||||||||||||||| |||||||||||||||||||||||||||||||||||
Sbjct  1     CCGGAGGCCGCCGTCGCCGCTGGAGCGGGCGATCAGAAGCGCGTCGTGGACGAGGACGAC  60

Query  567   GTCTTCGAGATGCCGCGGCTGCTGGTGAGCATGGCCGAGGGCTTGATGATGAGCCCGCCG  626
             ||||||||||||||||||||||||||||||||||||||||||||||||||||||||||||
Sbjct  61    GTCTTCGAGATGCCGCGGCTGCTGGTGAGCATGGCCGAGGGCTTGATGATGAGCCCGCCG  120

Query  627   AGGCTGAGCCCCTCGACGGACGGCGTCGGCGGCGTGTCGCCGGAGGACGACGAGGACGAG  686
             ||||||||||||||||||||||||||||||||||||||||||||||||||||||||||||
Sbjct  121   AGGCTGAGCCCCTCGACGGACGGCGTCGGCGGCGTGTCGCCGGAGGACGACGAGGACGAG  180

Query  687   GACGGCATGAGCCTGTGGAACCATTCCTGAAATGGGCTTACGGGTGCATAGCATTTGCAG  746
             ||||||||||||||||||||||||||||||||||||||||||||||||||||||||||||
Sbjct  181   GACGGCATGAGCCTGTGGAACCATTCCTGAAATGGGCTTACGGGTGCATAGCATTTGCAG  240

Query  747   GAGGACACCATGTATTTTCCATGTGTTCGTATGAATTCTGTGCCTACGTACAGTACGTAC  806
             ||||||||||||||||||||||||||||||||||||||||||||||||||||||||||||
Sbjct  241   GAGGACACCATGTATTTTCCATGTGTTCGTATGAATTCTGTGCCTACGTACAGTACGTAC  300

Query  807   TCGGCACGTATGCGAGATAGGAACGGAGCAGAGCAGGATACTGAATTTTATCGTAGTTGT  866
             ||||||||||||||||||||||||||||||||||||||||||||||||||||||||||||
Sbjct  301   TCGGCACGTATGCGAGATAGGAACGGAGCAGAGCAGGATACTGAATTTTATCGTAGTTGT  360

Query  867   CCTGTTACGTGTATACGGTATATATGGTCACTCCACTGATGTATAAACCATCCGTAGACG  926
             ||||||||||||||||||||||||||||||||||||||||||||||||||||||||||||
Sbjct  361   CCTGTTACGTGTATACGGTATATATGGTCACTCCACTGATGTATAAACCATCCGTAGACG  420

Query  927   AATTATGGCTGAAAACGATGCCCCTG  952
             ||||||||||||||||||||||||||
Sbjct  421   AATTATGGCTGAAAACGATGCCCCTG  446
```

Table of SNPs and InDels in Os03t0117900-01

	cds	5' UTR	3'UTR	Introns
No. of variants:1	1	0	0	0
No. of SNPs: 1	1	0	0	0
No.of InDels: 0	0	0	0	0

S.No: 19

D19: Os08t0509600-01

Query: KMR3: scaffold3452_size19961

Subject: IL50-13: scaffold2693_size19548

Score	Expect	Identities	Gaps	Strand
6571 bits(3558)	0.0	3566/3570(99%)	0/3570(0%)	Plus/Plus

```
Query  1    TTCCGtctctttcctctctcttctctctccccctctcCTggaggagagagaggagaagag  60
            ||||||||||||||||||||||||||||||||||||||||||||||||||||||||||||
Sbjct  1    TTCCGTCTCTTTCCTCTCTCTTCTCTCTCCCCCTCTCCTGGAGGAGAGAGAGGAGAAGAG  60

Query  61   gagggggggCCGCGCCAAGAGCCACGCGCTACAGTCTCCTTCCCACCCGCGACCGCGA  120
            ||||||||||||||||||||||||||||||||||||||||||||||||||||||||||||
Sbjct  61   GAGGGGGGGCCGCGCCAAGAGCCACGCGCTACAGTCTCCTTCCCACCCGCGACCGCGA  120

Query  121  GCAATGGAGATGGCCAGTGGAGGAGGcgccgccgccgccgccgGCGGCGGAGTAGGCGGC  180
            ||||||||||||||||||||||||||||||||||||||||||||||||||||||||||||
Sbjct  121  GCAATGGAGATGGCCAGTGGAGGAGGCGCCGCCGCCGCCGCCGGCGGCGGAGTAGGCGGC  180

Query  181  AGCGGCGGCGGTGGTGGTGGAGGGGACGAGCACCGCCAGCTGCACGGTCTCAAGTTCGGC  240
            ||||||||||||||||||||||||||||||||||||||||||||||||||||||||||||
Sbjct  181  AGCGGCGGCGGTGGTGGTGGAGGGGACGAGCACCGCCAGCTGCACGGTCTCAAGTTCGGC  240

Query  241  AAGAAGATCTACTTCGAGGAcgccgccgcggcagcaggcggcggcggcactggcagtggc  300
            ||||||||||||||||||||||||||||||||||||||||||||||||||||||||||||
Sbjct  241  AAGAAGATCTACTTCGAGGACGCCGCCGCGGCAGCAGGCGGCGGCGGCACTGGCAGTGGC  300

Query  301  agtggcagcgcgagcgccgcgccgccgTCCTCGTCTTCCAAGGCGGCGGGTGGTGGACGC  360
            ||||||||||||||||||||||||||||||||||||||||||||||||||||||||||||
Sbjct  301  AGTGGCAGCGCGAGCGCCGCGCCGCCGTCCTCGTCTTCCAAGGCGGCGGGTGGTGGACGC  360

Query  361  GGCGGAGGGGGCAAGAACAAGGGGAAGGGCGTGGCCGCGGCGGCGCCAccgccgccgccg  420
            ||||||||||||||||||||||||||||||||||||||||||||||||||||||||||||
Sbjct  361  GGCGGAGGGGGCAAGAACAAGGGGAAGGGCGTGGCCGCGGCGGCGCCACCGCCGCCGCCG  420

Query  421  ccgccgccgcGGTGCCAGGTGGAGGGGTGCGGCGCGGATCTGAGCGGGATCAAGAACTAC  480
            ||||||||||||||||||||||||||||||||||||||||||||||||||||||||||||
Sbjct  421  CCGCCGCCGCGGTGCCAGGTGGAGGGGTGCGGCGCGGATCTGAGCGGGATCAAGAACTAC  480

Query  481  TACTGCCGCCACAAGGTGTGCTTCATGCATTCCAAGGCTCCCCGCGTCGTCGTCGCCGGC  540
            ||||||||||||||||||||||||||||||||||||||||||||||||||||||||||||
Sbjct  481  TACTGCCGCCACAAGGTGTGCTTCATGCATTCCAAGGCTCCCCGCGTCGTCGTCGCCGGC  540
```

```
Query  541   CTCGAGCAGCGCTTCTGCCAGCAGTGCAGCAGGTCACTCTCTCACTCACCTCGCCATTGC  600
             ||||||||||||||||||||||||||||||||||||||||||||||||||||||||||||
Sbjct  541   CTCGAGCAGCGCTTCTGCCAGCAGTGCAGCAGGTCACTCTCTCACTCACCTCGCCATTGC  600

Query  601   TGATGTCACCACTGCTTTTGCTTTGCTTTGCTTGCTCTCCCTCCTCTTTCACCTATCTCT  660
             ||||||||||||||||||||||||||||||||||||||||||||||||||||||||||||
Sbjct  601   TGATGTCACCACTGCTTTTGCTTTGCTTTGCTTGCTCTCCCTCCTCTTTCACCTATCTCT  660

Query  661   CTTGTTTATTTGCTTCTTGTTCTTGTTTAGTGCTAGTACAtgtgttgttattgttgtgcc  720
             ||||||||||||||||||||||||||||||||||||||||||||||||||||||||||||
Sbjct  661   CTTGTTTATTTGCTTCTTGTTCTTGTTTAGTGCTAGTACATGTGTTGTTATTGTTGTGCC  720

Query  721   gttttgtcttttgggttattgtgttgttgttACTACTCGTTTTACTATAGGTTTTTAAGG  780
             ||||||||||||||||||||||||||||||||||||||||||||||||||||||||||||
Sbjct  721   GTTTTGTCTTTTGGGTTATTGTGTTGTTGTTACTACTCGTTTTACTATAGGTTTTTAAGG  780

Query  781   TTTATGAGCACGGCCACCACATTAGATGCACTGTCAAGTGGTGTGTGTGGGACCTTTCCT  840
             ||||||||||||||||||||||||||||||||||||||||||||||||||||||||||||
Sbjct  781   TTTATGAGCACGGCCACCACATTAGATGCACTGTCAAGTGGTGTGTGTGGGACCTTTCCT  840

Query  841   GCTAAAACAAGCTGATTTCAACTCTCTGAAACTTCCTGCATTTCATCTATTTTTATCTTT  900
             ||||||||||||||||||||||||||||||||||||||||||||||||||||||||||||
Sbjct  841   GCTAAAACAAGCTGATTTCAACTCTCTGAAACTTCCTGCATTTCATCTATTTTTATCTTT  900

Query  901   GATTGTGTTGGGAGTACTACACTAGTAGTGTTAATATTTTGACTGGTGCTTATGAGATTT  960
             ||||||||||||||||||||||||||||||||||||||||||||||||||||||||||||
Sbjct  901   GATTGTGTTGGGAGTACTACACTAGTAGTGTTAATATTTTGACTGGTGCTTATGAGATTT  960

Query  961   TTAAGTTGGTAGGTTGATGAGGAAAATACTCCTTTATATGGTTGAGTGATGTGACTTGCC  1020
             ||||||||||||||||||||||||||||||||||||||||||||||||||||||||||||
Sbjct  961   TTAAGTTGGTAGGTTGATGAGGAAAATACTCCTTTATATGGTTGAGTGATGTGACTTGCC  1020

Query  1021  TGTCTGCCTGCCTGCCTGCCGCTTTGCATAAGATTCCTCTGTGTTAGTAAGAGCCACTGT  1080
             ||||||||||||||||||||||||||||||||||||||||||||||||||||||||||||
Sbjct  1021  TGTCTGCCTGCCTGCCTGCCGCTTTGCATAAGATTCCTCTGTGTTAGTAAGAGCCACTGT  1080

Query  1081  TTATTTGTACTGGTGCTTACTCTACTTAGTTAATTAGCCATTAGCTATAAAATTCCGTTG  1140
             ||||||||||||||||||||||||||||||||||||||||||||||||||||||||||||
Sbjct  1081  TTATTTGTACTGGTGCTTACTCTACTTAGTTAATTAGCCATTAGCTATAAAATTCCGTTG  1140

Query  1141  ATGTTGCAAGCTTAGCAATGGCCACGGTAAGAATGGGAGAGAGAAGTTGGCTAAAGCTGT  1200
             ||||||||||||||||||||||||||||||||||||||||||||||||||||||||||||
Sbjct  1141  ATGTTGCAAGCTTAGCAATGGCCACGGTAAGAATGGGAGAGAGAAGTTGGCTAAAGCTGT  1200

Query  1201  TGCTTTGTAGTTTGTACTATATATGTGTCTTTGTGTTGCAAGATATGCAACTCCTACTAT  1260
             ||||||||||||||||||||||||||||||||||||||||||||||||||||||||||||
Sbjct  1201  TGCTTTGTAGTTTGTACTATATATGTGTCTTTGTGTTGCAAGATATGCAACTCCTACTAT  1260
```

```
Query  1261  GCTGTGACTTGAGCTCAAGGTTTTCAGTTATCTATAGATCCTTACTACTACTGAGCATAC  1320
             ||||||||||||||||||||||||||||||||||||||||||||||||||||||||||||
Sbjct  1261  GCTGTGACTTGAGCTCAAGGTTTTCAGTTATCTATAGATCCTTACTACTACTGAGCATAC  1320

Query  1321  TACCACTTCTGTATGGTAGCATATGGTAGCATAGTCCAAGTTCCAACGCCTCGCCAGTTG  1380
             ||||||||||||||||||||||||||||||||||||||||||||||||||||||||||||
Sbjct  1321  TACCACTTCTGTATGGTAGCATATGGTAGCATAGTCCAAGTTCCAACGCCTCGCCAGTTG  1380

Query  1381  TTCATAATCTATACTACCACTTCTGTGCATTTGTTACTTTTATTTAATAGTTTGTCTCAT  1440
             ||||||||||||||||||||||||||||||||||||||||||||||||||||||||||||
Sbjct  1381  TTCATAATCTATACTACCACTTCTGTGCATTTGTTACTTTTATTTAATAGTTTGTCTCAT  1440

Query  1441  TAGCTGACAAGCATATGCCTGTTTTGATATCTGCCCCTCTTGTAATAGTCTATGGATAGC  1500
             ||||||||||||||||||||||||||||||||||||||||||||||||||||||||||||
Sbjct  1441  TAGCTGACAAGCATATGCCTGTTTTGATATCTGCCCCTCTTGTAATAGTCTATGGATAGC  1500

Query  1501  TTGGACTGTTTGATGCTTTAATTTTTTACTAGCAACACTTAGGGCCCCTTTGAAATGGAG  1560
             ||||||||||||||||||||||||||||||||||||||||||||||||||||||||||||
Sbjct  1501  TTGGACTGTTTGATGCTTTAATTTTTTACTAGCAACACTTAGGGCCCCTTTGAAATGGAG  1560

Query  1561  GATTAGCAAAGGAATTTTGGAGGATTCATTTTCCTAAGGATTTTTTCCTATAGAGCCATT  1620
             ||||||||||||||||||||||||||||||||||||||||||||||||||||||||| ||
Sbjct  1561  GATTAGCAAAGGAATTTTGGAGGATTCATTTTCCTAAGGATTTTTTCCTATAGAGCCCTT  1620

Query  1621  TGATTCATAGAAAGAGGATAGGAAAACTTCCGTAGGATTGCATTCCTATGATCAATTCCA  1680
             ||||||||||||||||||||||||||||||||||||||||||||||||||||||||||||
Sbjct  1621  TGATTCATAGAAAGAGGATAGGAAAACTTCCGTAGGATTGCATTCCTATGATCAATTCCA  1680

Query  1681  TAGGAAAATAAGCAAGAGGTTAGACCTCTTGTGAAACTTTCCTTTGTTGAGTGTATTCTG  1740
             ||||||||||||||||||||||||||||||||||||||||||||||||||||||||  ||
Sbjct  1681  TAGGAAAATAAGCAAGAGGTTAGACCTCTTGTGAAACTTTCCTTTGTTGAGTGTATCTTG  1740

Query  1741  TGGTATAATCAAAGGGCTCTTCTCTCCATTTCCTGTGTTTTCAATTCCTGTAGGATTGGA  1800
             |||||||||||||||||||||||||||||||| |||||||||||||||||||||||||||
Sbjct  1741  TGGTATAATCAAAGGGCTCTTCTCTCCATTTCATGTGTTTTCAATTCCTGTAGGATTGGA  1800

Query  1801  AAAACATACAACTTCAATTCCTACGTTTTTCCTATTCCTATGTTTTTCCTATCCTGCGTT  1860
             ||||||||||||||||||||||||||||||||||||||||||||||||||||||||||||
Sbjct  1801  AAAACATACAACTTCAATTCCTACGTTTTTCCTATTCCTATGTTTTTCCTATCCTGCGTT  1860

Query  1861  TCAAAGGGGCCCTTAAGGATGAAGGGAAGTAAGAGAAACATACTAGAGAATATGTAGTAG  1920
             ||||||||||||||||||||||||||||||||||||||||||||||||||||||||||||
Sbjct  1861  TCAAAGGGGCCCTTAAGGATGAAGGGAAGTAAGAGAAACATACTAGAGAATATGTAGTAG  1920

Query  1921  TATTTCTACATTCCATATTTGTAGCACTAGCCCACAAATATCTTTGCCTTGTACTTACTT  1980
             ||||||||||||||||||||||||||||||||||||||||||||||||||||||||||||
Sbjct  1921  TATTTCTACATTCCATATTTGTAGCACTAGCCCACAAATATCTTTGCCTTGTACTTACTT  1980

Query  1981  CATACCAGTTCCCCCCTTTTCAGAGCAAACCAACAATTTCTGTTGCCTTATATATCTAGT  2040
             ||||||||||||||||||||||||||||||||||||||||||||||||||||||||||||
Sbjct  1981  CATACCAGTTCCCCCCTTTTCAGAGCAAACCAACAATTTCTGTTGCCTTATATATCTAGT  2040
```

```
Query  2041  GTCTTCGTACTAATATATCTGTTCCAAAATGTACCTGTCCAAATTCATAGCTAGAAATAG  2100
             ||||||||||||||||||||||||||||||||||||||||||||||||||||||||||||
Sbjct  2041  GTCTTCGTACTAATATATCTGTTCCAAAATGTACCTGTCCAAATTCATAGCTAGAAATAG  2100

Query  2101  CTTTATTTAGGACGGAAGTAATAACTGTTGTTAGAGACTTGGTTCAGACTTTTGGTTATG  2160
             ||||||||||||||||||||||||||||||||||||||||||||||||||||||||||||
Sbjct  2101  CTTTATTTAGGACGGAAGTAATAACTGTTGTTAGAGACTTGGTTCAGACTTTTGGTTATG  2160

Query  2161  TTGAGGCTACTATCATTTCCTTTACGGGCCAAATTACTACAAATGAGAATTCATAAAAAT  2220
             ||||||||||||||||||||||||||||||||||||||||||||||||||||||||||||
Sbjct  2161  TTGAGGCTACTATCATTTCCTTTACGGGCCAAATTACTACAAATGAGAATTCATAAAAAT  2220

Query  2221  GTCAAGATTTTATGATTGTTGTAGCTTTATTTAGGACGGAGGTAGTAATTGTTGTTAGAG  2280
             ||||||||||||||||||||||||||||||||||||||||||||||||||||||||||||
Sbjct  2221  GTCAAGATTTTATGATTGTTGTAGCTTTATTTAGGACGGAGGTAGTAATTGTTGTTAGAG  2280

Query  2281  ACTTGGTTCAGACTTTTGGTTACGTTGAAGCTACTATCATTTCCTTTATGGTCAAATTAC  2340
             ||||||||||||||||||||||||||||||||||||||||||||||||||||||||||||
Sbjct  2281  ACTTGGTTCAGACTTTTGGTTACGTTGAAGCTACTATCATTTCCTTTATGGTCAAATTAC  2340

Query  2341  TAACAATGAGTATTCATAAAAATGTCAAGATTTTATAATTGAGCTGTGCCAGTGCTAAGT  2400
             ||||||||||||||||||||||||||||||||||||||||||||||||||||||||||||
Sbjct  2341  TAACAATGAGTATTCATAAAAATGTCAAGATTTTATAATTGAGCTGTGCCAGTGCTAAGT  2400

Query  2401  GTGTCACTATCTGATGCCATAATGCATCATTATAAAAGCCAGATGGACCATTAGCTTTTA  2460
             ||||||||||||||||||||||||||||||||||||||||||||||||||||||||||||
Sbjct  2401  GTGTCACTATCTGATGCCATAATGCATCATTATAAAAGCCAGATGGACCATTAGCTTTTA  2460

Query  2461  TGTGTAGGACACCTGCCGTCCAATTAGATGGATAACCATCTAGTGTTTGTGTACTGTTAT  2520
             ||||||||||||||||||||||||||||||||||||||||||||||||||||||||||||
Sbjct  2461  TGTGTAGGACACCTGCCGTCCAATTAGATGGATAACCATCTAGTGTTTGTGTACTGTTAT  2520

Query  2521  TTTAAGCCCGACATCTCACAACTCCATGAATGATTACAGTCTTCCTTTCACATGGTGTCC  2580
             ||||||||||||||||||||||||||||||||||||||||||||||||||||||||||||
Sbjct  2521  TTTAAGCCCGACATCTCACAACTCCATGAATGATTACAGTCTTCCTTTCACATGGTGTCC  2580

Query  2581  TTTTGTTGTGTTAGGAATAGCATTTTTTATTTATGGGTGTAATTATGAAAGGCACTAGGA  2640
             ||||||||||||||||||||||||||||||||||||||||||||||||||||||||||||
Sbjct  2581  TTTTGTTGTGTTAGGAATAGCATTTTTTATTTATGGGTGTAATTATGAAAGGCACTAGGA  2640

Query  2641  GAGTTGCTGCTTTATCTTGATGGGATTTGTAGTAATACCATCTTTAGGATGACAAGAAAT  2700
             ||||||||||||||||||||||||||||||||||||||||||||||||||||||||||||
Sbjct  2641  GAGTTGCTGCTTTATCTTGATGGGATTTGTAGTAATACCATCTTTAGGATGACAAGAAAT  2700

Query  2701  CTTGTTCTGAGTTAGCATGGGCTGCCTTTTGACCTGAGCTACGGTTTGCTATGTTTGGCT  2760
             ||||||||||||||||||||||||||||||||||||||||||||||||||||||||||||
Sbjct  2701  CTTGTTCTGAGTTAGCATGGGCTGCCTTTTGACCTGAGCTACGGTTTGCTATGTTTGGCT  2760

Query  2761  TGCATCATGCAGATCTATTAGGATAATAAGCATATAAAAGTTGCTTGCATTGTGCATTGC  2820
             ||||||||||||||||||||||||||||||||||||||||||||||||||||||||||||
Sbjct  2761  TGCATCATGCAGATCTATTAGGATAATAAGCATATAAAAGTTGCTTGCATTGTGCATTGC  2820
```

```
Query  2821  TTGTTTTACCTTGATTCATGTAGGAGTAATTTGCTCGCCATGCCTCGTTTTGCTTTCTGA  2880
             ||||||||||||||||||||||||||||||||||||||||||||||||||||||||||||
Sbjct  2821  TTGTTTTACCTTGATTCATGTAGGAGTAATTTGCTCGCCATGCCTCGTTTTGCTTTCTGA  2880

Query  2881  GTCAACAGCCAAATTTAGATGATGTACCTTCTGTTGCTTCAAAAACTCAGTCACTGCACA  2940
             ||||||||||||||||||||||||||||||||||||||||||||||||||||||||||||
Sbjct  2881  GTCAACAGCCAAATTTAGATGATGTACCTTCTGTTGCTTCAAAAACTCAGTCACTGCACA  2940

Query  2941  GCAGCAGTGGATAGGATTCAGAATCAATCTATCCATGATTCTCTGTTCACATAATATGAC  3000
             ||||||||||||||||||||||||||||||||||||||||||||||||||||||||||||
Sbjct  2941  GCAGCAGTGGATAGGATTCAGAATCAATCTATCCATGATTCTCTGTTCACATAATATGAC  3000

Query  3001  AGGTTCCACCTGCTGCCTGAATTTGACCAAGGAAAACGCAGCTGCCGCAGACGCCTTGCA  3060
             ||||||||||||||||||||||||||||||||||||||||||||||||||||||||||||
Sbjct  3001  AGGTTCCACCTGCTGCCTGAATTTGACCAAGGAAAACGCAGCTGCCGCAGACGCCTTGCA  3060

Query  3061  GGTCATAATGAGCGCCGGAGGAGGCCGCAAACCCCTTTGGCATCACGCTACGGTCGACTA  3120
             ||||||||||||||||||||||||||||||||||||||||||||||||||||||||||||
Sbjct  3061  GGTCATAATGAGCGCCGGAGGAGGCCGCAAACCCCTTTGGCATCACGCTACGGTCGACTA  3120

Query  3121  GCTGCATCTGTTGGTGGTATCATCAGAGGCTCTTGTTTTCTTTGCATCTTGTGTGTTTGT  3180
             ||||||||||||||||||||||||||||||||||||||||||||||||||||||||||||
Sbjct  3121  GCTGCATCTGTTGGTGGTATCATCAGAGGCTCTTGTTTTCTTTGCATCTTGTGTGTTTGT  3180

Query  3181  TGGTAACTACTGGTTGCATTCGCTGATGTGTTGTTTGTTGCGATTCTTGATCCAGAAGAG  3240
             ||||||||||||||||||||||||||||||||||||||||||||||||||||||||||||
Sbjct  3181  TGGTAACTACTGGTTGCATTCGCTGATGTGTTGTTTGTTGCGATTCTTGATCCAGAAGAG  3240

Query  3241  CATCGCAGGTTCAGAAGCTTTACGTTGGATTTCTCCTACCCAAGGGTTCCAAGCAGCGTA  3300
             ||||||||||||||||||||||||||||||||||||||||||||||||||||||||||||
Sbjct  3241  CATCGCAGGTTCAGAAGCTTTACGTTGGATTTCTCCTACCCAAGGGTTCCAAGCAGCGTA  3300

Query  3301  AGGAATGCATGGCCAGCAATTCAACCAGGCGATCGGATCTCCGGTGGTATCCAGTGGCAC  3360
             ||||||||||||||||||||||||||||||||||||||||||||||||||||||||||||
Sbjct  3301  AGGAATGCATGGCCAGCAATTCAACCAGGCGATCGGATCTCCGGTGGTATCCAGTGGCAC  3360

Query  3361  AGGAACGTAGCTCCTCATGGTCACTCTAGTGCAGTGGCGGGATATGGTGCCAACACATAC  3420
             ||||||||||||||||||||||||||||||||||||||||||||||||||||||||||||
Sbjct  3361  AGGAACGTAGCTCCTCATGGTCACTCTAGTGCAGTGGCGGGATATGGTGCCAACACATAC  3420

Query  3421  AGCGGCCAAGGTAGCTCTTCTTCAGGGCCACCGGTGTTCGCTGGCCCAAATCTCCCTCCA  3480
             ||||||||||||||||||||||||||||||||||||||||||||||||||||||||||||
Sbjct  3421  AGCGGCCAAGGTAGCTCTTCTTCAGGGCCACCGGTGTTCGCTGGCCCAAATCTCCCTCCA  3480

Query  3481  GGTGGATGTCTCGCAGGGGTCGGTGCCGCCACCGACTCGAGCTGTGCTCTCTCTCTTCTG  3540
             ||||||||||||||||||||||||||||||||||||||||||||||||||||||||||||
Sbjct  3481  GGTGGATGTCTCGCAGGGGTCGGTGCCGCCACCGACTCGAGCTGTGCTCTCTCTCTTCTG  3540

Query  3541  TCAACCCAGCCATGGGATACTACTACCCAC  3570
             ||||||||||||||||||||||||||||||
Sbjct  3541  TCAACCCAGCCATGGGATACTACTACCCAC  3570
```

Table of SNPs and InDels in Os08t0509600-01

	cds	5' UTR	3'UTR	Introns
No. of variants:4	0	0	0	4
No. of SNPs: 4	0	0	0	4
No.of InDels: 0	0	0	0	0

Table S12. Effect of SNPs in the 2kb upstream region of the gene in IL 50-13

(C4: Os02t0831500-01 (Chr02:35754946..35761216) (+ strand))

**S. No.**	**Position**	**SNP change**	**SNP effect**
1	35753912	C-T	MODIFIER
2	35753931	T-C	MODIFIER
3	35753961	C-T	MODIFIER

Supplementary Table legends

Table S1. List of significantly (*p* <=0.05) up-regulated and down-regulated genes in flag leaf and panicle of test hybrid compared to control hybrid

Table S2. List of DEGs up- and down-regulated in common and exclusive in flag leaf and panicle of control hybrid Vs test hybrid

Table S3. DEGs and reported yield related genes considered for analysis to study the differences in the gene sequences between KMR3 and IL50-13. Category A refers to genes within *qyld2.1*, B refers to genes within *qyld8.2*, C refers to genes showing high fold expression and D refers to reported yield related genes.

Table S4. Differential expression of genes in flag leaf associated with grain yield and abiotic stress tolerance in test hybrid

Table S5. Differential expression of genes in panicle associated with grain yield and abiotic stress tolerance in test hybrid

Table S6. Enriched GO terms in flag leaf up-and down-regulated DEGs of test hybrid

Table S7. Enriched GO terms in panicle up-and down-regulated DEGs of test hybrid

Table S8. The GO terms common between flag leaf and panicle of test hybrid

Table S9. List of flag leaf up and down-regulated genes involved metabolisms of test hybrid.

Table S10. List of up and down-regulated genes involved metabolisms in panicle of test hybrid.

Table S11. Sequences of the 19 genes showing differences between KMR3 and IL50-13.

Table S12. Effect of SNPs in the 2kb upstream region of the gene in IL50-13
